# Wubie Fanchun Formula-inducible metabolites in primary ovarian insufficiency model mice that facilitate ovarian renovation

**DOI:** 10.1080/13880209.2026.2668132

**Published:** 2026-05-12

**Authors:** Yun Chen, Shanshan Chen, Bingbing Song, Xinfang Xia, Kaiqing Zhang, Li Chen, Lei Chi, Qiong Wang

**Affiliations:** ^a^ Affiliated Hospital of Nanjing University of Chinese Medicine, Nanjing, PR China; ^b^ Jiangsu Province Hospital of Chinese Medicine, Nanjing, Jiangsu, PR China; ^c^ No.1 Clinical Medical College, Nanjing University of Chinese Medicine, Nanjing, PR China

**Keywords:** Primary ovarian insufficiency, Wubie Fanchun Formula, PI3K/AKT/FOXO3a pathway, phosphoinositol 38:5, glutamine

## Abstract

**Context:**

Premature ovarian insufficiency (POI), defined as ovarian activity cessation before age 40, significantly impacts both fertility and long-term health, with currently inadequate treatment options available. Wubie Fanchun Formula (WBFC) has been clinically used for over 20 years, showing benefits in TCM symptoms and hormone levels in POI patients with liver-kidney deficiency. However, its active components and mechanistic basis are still largely unknown.

**Objective:**

To characterize the active ingredients and delineate the pharmacological basis of WBFC’s action in managing POI.

**Materials and methods:**

LC-TOF-MS was used to identify WBFC’s chemical constituents. Network pharmacology analysis pinpointed candidate mechanisms, which were tested in a 4-VCD-induced POI mouse model. Metabolic dysfunction was characterized *via* pyruvate tolerance tests, liver glycogen staining, and untargeted metabolomics. Key metabolites regulated by WBFC in model mice, phosphatidylinositol 38:5 (PI) and glutamine (GLN), were supplemented to validate their roles in POI pathology.

**Results:**

25 bioactive constituents in WBFC were found by LC-TOF-MS analysis. WBFC ameliorated ovarian dysfunction and activated the PI3K/AKT/FOXO3a pathway in parallel with simultaneously improving systemic metabolic parameters. Metabolomics identified PI and GLN among the top 30 most significantly altered metabolites, both showing marked depletion in POI mice and substantial restoration following WBFC treatment. Functional validation through exogenous supplementation demonstrated that combined PI and GLN administration effectively rescued ovarian dysfunction and specifically reactivated the ovarian PI3K-AKT pathway in 4-VCD induced POI mice.

**Discussion and conclusion:**

Amino acids, saponins, anthraquinones, and carbohydrates are the main chemical components of WBFC. WBFC ameliorated POI and activated PI3K/AKT/FOXO3a signaling through metabolic regulation, with PI and GLN identified as critical therapeutic mediators.

## Introduction

Primary ovarian insufficiency (POI), characterized by the diminished ovarian function in women under 40, afflicts nearly 3.5% of the female population globally (Li et al. 2023). It is a disease not only affects wellbeing and female fertility, but also associated with enhanced probability of osteoporosis and fracture, cardiovascular disease, dementia as well as all-cause mortality if untreated (Nash and Davies 2024). The pathogenesis of POI is intricate, encompassing genetic factors, autoimmune damage, metabolic abnormalities, infections, iatrogenic factors, and various environmental influence (Ishizuka 2021). Hormone therapy has been recommended for women with POI until the natural menopause age according to guidelines from the European Society of Human Reproduction and Embryology (ESHRE), the National Institute for Health and Care Excellence (NICE), and the International Menopause Society (IMS) (Nash and Davies 2024). Although it is generally safe, patients with POI usually hesitate to apply hormone therapy concerning the potential risks for breast cancer, stoke, or thromboembolic disease (Armeni et al. 2021). Many women use herbal supplements to treat POI, while some reports suggest that the effectiveness of this therapy is very limited (Nash and Davies 2024). Thus, inadequate therapeutic options are left for women diagnosed with POI. It is of utmost urgency to devise effective therapeutic strategies, thereby enhancing the quality of life for women.

Considering the complexity of the causes and the personalized treatment of POI, traditional Chinese medicine (TCM) prescriptions are considered as a promising strategy for managing POI. Evidence has been reported recently that Huyang Yangkun (HYYK) formula effectively improved menopause-related symptoms in POI patients in a multicenter, randomized, double-blind, placebo-controlled study (Peng et al. 2025). In another randomized, double-blind, placebo-controlled trial of POI women, Yangyin Shugan formula was found to improve menopausal symptoms, basal hormone levels, and ovarian functions (Cao et al. 2018). Accumulating studies have reported that Si-wu-tang (Zhou et al. 2021; Liu et al. 2023), Kuntai capsule (Gong et al. 2024), Zigui-Yichong-Fan (Xiu et al. 2023), Jiajian Guishen Formula (Su et al. 2021), Heshi Yu Lin formula (Huang et al. 2024) alleviate POI in mice model and Danggui Buxue Tang (Wang et al. 2021), Zuogui Pills (Peng et al. 2019), BushenHuoxue Formula (Miao et al. 2020), Jinfeng Pills (Hu et al. 2023) attenuate POI in rat’s model. The therapeutic mechanism of TCM formulas were explored, which mainly involve inhibiting apoptosis and oxidative stress (Jiang et al. 2022; Chen et al. 2024; Xu et al. 2024), improving granulosa cell proliferation and energy metabolism (Kurihara 1985), and regulating ovarian PI3K-AKT signaling (Liu et al. 2023; Su et al. 2023). These results indicate that with the expanding usage of TCM prescriptions in POI, it is crucial to utilize modern science to unravel the basis of TCM diagnosis and treatment.

Modern medicine has found that approximately 90% of spontaneous POI cases have no identified cause, like what TCM did, approaching the pattern differentiation of POI diagnosis. To treat POI identified as an insufficiency of kidney and liver pattern, Professor Guicheng Xia, a renowned TCM Master in China, used WBFC as basic formula. It is composed of seven traditional Chinese medicinal herbs: *Trionyx sinensis* Wiegmann. Trionychidae; *Fallopia multiflora* (Thunb.) Haraldson, Polygonaceae; *Lycium barbarum* L. Solanaceae; *Dipsacus asper* Wall. ex Henry, Caprifoliaceae; *Atractylodes macrocephala* Koidz. Asteraceae; *Poria cocos* (Schw.) Wolf. Polyporaceae and *Canis familiaris* L. Canidae. With over two decades of clinical application, WBFC has demonstrated notable therapeutic efficacy. It can markedly improve clinical symptoms in POI patients, reduce serum levels of follicle-stimulating hormone (FSH) and luteinizing hormone (LH), increase estradiol (E2) levels, and regulate menstrual cycles to promote ovulation (Yu et al. 2024). However, the specific mechanisms through which WBFC exerts its therapeutic effects remain inadequately understood.

Recently, it was found that sleep deprivation could lead to POI in adolescent mice by changing gut microbiota and then affecting systemic metabolomics (Yan et al. 2024). Moreover, POI patients exhibited significant metabolic profile alterations in the plasma (Yu et al. 2023; Zhou et al. 2023), serum (Jiang et al. 2021), follicular fluid (Wang et al. 2024) and feces (Jiang et al. 2021). Metabolic disorder of the liver and kidney was also found in POI model mice and could be reversed by electro-acupuncture (Chen et al. 2022). Zishen Yutai pills attenuated POI in mice by regulating arachidonic acid metabolism and the AKT pathway (Dang et al. 2024). These findings highlight that metabolic dysfunctions are closely related to the pathogenesis of POI. As per of the principles of formulating WBFC and multiple metabolite categories such as androgenic steroids, fatty acid metabolism, amino acid metabolism causally linked to POI (Chen et al. 2024), we plan to use metabolomics methods to reveal the molecular events driving the anti-POI effect of WBFC formula.

This study presents a comprehensive characterization of WBFC by using LC-TOF-MS analysis, identifying amino acids, saponins, anthraquinones, and carbohydrates as its predominant bioactive constituents. Through comprehensive preclinical investigation, we demonstrate the significant therapeutic potential of WBFC in both ameliorating POI and modulating the PI3K/AKT/FOXO3a signaling pathway. Notably, our metabolite supplementation experiments in 4-VCD-induced POI mice model yielded pivotal insights: co-administration of two key WBFC-regulated metabolites, PI and GLN, synergistically improved ovarian function. This therapeutic effect was mediated through specific activation of the ovarian PI3K-AKT signaling cascade, providing direct evidence for the formula’s mode of action.

## Material and methods

### Drugs and reagents

4-VCD was purchased from Sigma-Aldrich (catalog number: 94956). The WBFC oral liquid (10 mL/vial) contained the following pharmacopeial ingredients: *Trionyx sinensis* Wiegmann. Trionychidae; *Fallopia multiflora* (Thunb.) Haraldson, Polygonaceae; *Lycium barbarum* L. Solanaceae; *Dipsacus asper* Wall. ex Henry, Caprifoliaceae; *Atractylodes macrocephala* Koidz. Asteraceae; *Poria cocos* (Schw.) Wolf. Polyporaceae and *Canis familiaris* L. Canidae. The formulation was authenticated and supplied by Jiangsu Provincial Hospital of Traditional Chinese Medicine (Medical Preparation Approval No. Z04001287, Jiangsu Provincial Medical Products Administration). Based on the yield of WBFC and its clinical dosage, the murine dose was determined according to species-specific dose conversion principles. The details are as follows: The daily clinical dosage of WBFC for adults is 1.65 g/ml, administered as 20 ml per day for a 70 kg individual, which corresponds to approximately 47.1 mg/kg. Following the pharmacological experimental methodology edited by Professor Shuyun Xu, the equivalent dose conversion factor between humans and mice is 9.1. Therefore, the equivalent dose for mice was calculated as 4.3 g/kg, which was designated as the high-dose group in this study. Using a two-fold dilution, half of this dose (2.15 g/kg) was assigned as the low-dose group. Accordingly, the administered doses for the WBFC-L and WBFC-H groups were 2.15 g/kg and 4.3 g/kg, respectively. PI Solution preparation: PI (Sigma, 840042 P) was dissolved in 5 mM HEPES buffer (pH 7.4) supplemented with 1 mM EDTA, followed by continuous stirring at ambient temperature (Shimizu et al. 2010). GLN solution preparation: glutamine powder (Sigma, G-3126) was dissolved in physiological saline to prepare GLN solution at the required concentration. The solution was stored at 4 °C and freshly taken before each use (Luo et al. 2019).

### Equipment and instruments

High-performance liquid chromatography system (Agilent, USA). High-resolution mass spectrometer (AB Sciex, USA). Electrophoresis power supply, electrophoresis chamber, and membrane transfer tank (Bio-rad, USA). Tanon imaging system (Tanon, China). Microplate reader (Bio Tek, USA). Upright fluorescence microscope (Nikon, Japan). Tissue dehydrator and embedding machine (Junjie, Wuhan). Other experimental reagents and consumables are listed in Supplemental Table 1. Antibody information is provided in Supplemental Table 2.

### Liquid chromatography-time of flight mass spectrometry (LC-TOF/MS) analysis of WBFC

The chemical constituents of WBFC Oral Liquid were characterized using an Agilent 1290 Infinity HPLC system coupled with an AB Sciex TripleTOF^™^ 5600+ high-resolution mass spectrometer. The cartridges were activated with 3 mL methanol and equilibrated with 3 mL ultrapure water. After vortex-mixing (30 s), 600 μL of the oral liquid was loaded onto the cartridge, washed with 2 mL water to remove impurities, and eluted with 1 mL methanol. The eluate was vortex-mixed (30 s), centrifuged (12,000 × g, 4 °C, 5 min), and 5 μL of the supernatant was injected for analysis. Chromatographic separation was achieved on an Agilent Poroshell 120 SB-C18 pre-column (3.0 mm × 5.0 mm, 2.7 μm) with mobile phase A (2 mmol/L ammonium formate and 0.1% formic acid in water) and mobile phase B (2 mmol/L ammonium formate and 0.1% formic acid in a 1:1 methanol-acetonitrile mixture). A gradient elution program was applied: 5% B (0–0.5 min), 5–100% B (0.5–12 min), 100% B (12–16 min), returning to 5% B (16–16.1 min), and held until 22 min, at a flow rate of 300 μL/min with column temperature maintained at 35 °C and injection chamber at 8 °C (Abdulhafiz et al. 2022). Mass spectrometric analysis was performed using an electrospray ionization (ESI) source in both positive and negative modes. The TOF-MS parameters that were employed are outlined below: m/z range 100–1000, accumulation time 0.250015 s per scan, ion source temperature 550 °C, nebulizing gas (GS1) and auxiliary gas (GS2) pressures at 60 psi, curtain gas at 35 psi, declustering potential (DP) 80 V, collision energy (CE) 10 eV, and spray voltages of 5500 V (positive) or 4500 V (negative). Data-dependent acquisition (DDA) with IDA employed the following criteria: an intensity threshold of >500 cps, an isotope exclusion window of 4 Da, a mass tolerance of 0.05 Da, and a maximum of 8 precursor ions per cycle for MS/MS. Product ion scans were conducted over m/z 50–1000 with an accumulation time of 0.100006 s and CE of 35 ± 15 eV. System calibration was performed automatically using the CDS calibration delivery system, and data acquisition was controlled by Analyst TF 1.6 software. For data processing, PeakView^™^ Software v1.2 (AB Sciex) was utilized to perform mass defect filtering (MDF), formula prediction, extracted ion chromatogram (XIC) analysis, and IDA exploration (Jia et al. 2024).

### Investigation of the mechanism of WBFC against POI **via** network pharmacology

#### Screening of active ingredients and action targets of WBFC

The canonical SMILES numbers for 25 compounds identified by TOF were obtained from the PubChem database. These SMILES were then entered into Swiss Target Prediction to obtain potential targets for the main active chemical composition. Then, the TCMSP database and PharmMapper database were used to obtain the targets of Poria cocos polysaccharides.

#### Prediction targets associated with POI

We used ‘Premature Ovarian Insufficiency’ as a search term in a series of databases (OMIM, GeneGards, DisGeNET, and DrugBank) and identified a range of disease targets related to POI. After that, we identified the intersection between POI disease targets and the predicted targets of the composition of WBFC.

#### Protein–protein interaction (PPI) network construction

To investigate the potential association between WBFC targets and POI, we identified candidate therapeutic targets of WBFC for POI by selecting overlapping targets between WBFC compounds and POI-related proteins. The overlapping proteins were then uploaded to STRING (version 11.5) to construct a PPI network; with a confidence score threshold set to 0.9. To analyze the topological properties of the PPI network, we utilized the MCODE and cytoHubba plugins in Cytoscape (version 3.9.1) to identify core protein modules. The MCC algorithm was applied, using a node score cutoff of 0.2 and a k-core value of 2, to extract densely interconnected subnetworks.

#### GO analysis and KEGG pathway enrichment analysis

The objective of the analysis was to elucidate the signaling pathways involved in WBFC action against POI by examining the common target genes of drugs and diseases. The intersecting targets were evaluated for enriched gene ontology (GO) functions and Kyoto Encyclopedia of Genes and Genomes (KEGG) pathways using the clusterProfiler package in R. Gene function was annotated *via* three modules: biological process (BP), molecular function (MF), and cellular component (CC), using GO analysis. Significantly enriched GO terms and KEGG pathways in the differentially expressed genes were those meeting the criterion of P value less than 0.05 and FDR (False Discovery Rate) less than 0.05.

#### Construction of herb-component-target-pathway network graphs

To better understand how WBFC works against POI, we imported data on the herbs, active components, targets, and signaling pathways into Cytoscape 3.2.1. This allowed us to visualize the relationships and pathways targeted by WBFC.

### Animals

Female C57BL/6 mice, 6–8 weeks old, 18–22 g, were purchased from Si Pei Fu (Suzhou) Biotechnology Co., Ltd (SCXK (SU) 2022-0006), housed in the SPF-grade mouse facility of the Basic Pharmacology Laboratory at Jiangsu Province Hospital of Chinese Medicine (usage license: SYXK (SU) 2022-0070), and were maintained under specific pathogen-free conditions controlled for temperature (22 ± 2 °C) and humidity (40–70%) with 12 h light–dark cycle and free access to food and water. This study utilized 40 mice to evaluate the therapeutic efficacy of WBFC and 30 mice to conduct the key metabolites validation. The sample size was determined based on pilot study results and the statistical requirements of individual experiments. The study protocol was established prior to the commencement of the research and was approved by the Experimental Animal Ethics Committee of the Affiliated Hospital of Nanjing University of Chinese Medicine (Jiangsu Province Hospital of Chinese Medicine), with approval number 2023-DW-047-03.

### Construction of POI mouse model

The present study utilized a mouse model of POI induced by 4-VCD. This compound selectively targets and depletes primordial and primary ovarian follicles, leading to the destruction of the follicular reserve and subsequent development of POI (Cao et al. 2020). The MOD group received an intraperitoneal injection of 160 mg/kg/day 4-VCD dissolved in sesame oil for 15 days, while the control group received an intraperitoneal injection of an equivalent volume of physiological saline (Cao et al. 2020). Starting from the 7th day of modeling, vaginal smears were performed daily at 9:00 AM. When the estrous cycle of most model mice became irregular, it indicated successful modeling of POI, and no exclusion of animals occurred in experimental group.

### Animal grouping and treatment

After a 7-day acclimation period, all mice were uniquely numbered and then randomly assigned to experimental groups using R language: a control (CON) group and a model (MOD) group. The POI model was established as previously described. Successfully modeled mice were further allocated into two independent experimental cohorts:

### WBFC therapeutic efficacy study

CON (*n* = 10): Daily oral gavage (i.g.) of saline for 28 days. MOD (*n* = 10): POI model + daily i.g. saline. WBFC-Low (WB-L, *n* = 10): POI model + WBFC (2.15 g/kg/day, i.g.). WBFC-High (WB-H, *n* = 10): POI model + WBFC (4.3 g/kg/day, i.g.).

### Key metabolites validation study

CON (*n* = 6): Daily intraperitoneal (i.p.) saline. MOD (*n* = 6): POI model + daily i.p. saline. PI (*n* = 6): POI model + phosphoinositol (2.4 mg/kg/two days, intravenous [i.v.]. GLN (*n* = 6): POI model + glutamine (0.75 g/kg/day, i.p.). PI+GLN (*n* = 6): POI model + combined PI (2.4 mg/kg/day, i.v.) and GLN (0.75 g/kg/day, i.p.). (Shimizu et al. 2010; Luo et al. 2019). All treatments were administered consecutively for 28 days. Shanshan Chen and Bingbing Song was aware of the group allocation throughout the entire experiment.

On the day subsequent to the cessation of treatment, Mice were anesthetized by inhalation of 3–5% isoflurane (vol/vol) delivered through a precision vaporizer using medical-grade oxygen at a flow rate of 1–2 L/min. A state of deep anesthesia is achieved when the animal’s respiratory rate has decreased by approximately 50% and the righting reflex is lost. Gently grasp the mouse by the scruff to restrain it, ensuring the eyes protrude, and collect approximately 0.5 mL of blood *via* retro-orbital bleeding. Each mouse is subjected to this procedure only once, and the animals were immediately euthanized by cervical dislocation. These procedures are carried out in the Basic Pharmacology Laboratory of the Affiliated Hospital of Nanjing University of Chinese Medicine. All studies involving laboratory animals followed the ARRIVE guidelines, and no adverse events were observed.

### Estrous cycle monitoring

Throughout the experiment, daily observations of vaginal smears were conducted to monitor the estrous cycle of the mice, while their weights were recorded every two days. The estrous cycle was observed at 9:00 AM each day. The normal estrous cycle in mice is categorized into four stages: proestrus, estrus, metestrus, and diestrus.

### Oral glucose tolerance test (OGTT) and intraperitoneal pyruvate tolerance test (PTT)

OGTT: Mice were fasted for 6 h before the OGTT experiment. Mice were orally administered glucose at a dose of 3 g/kg. Blood samples were collected from the tail tip at 0 min and 15, 30, 90, and 120 min after gavage to measure glucose levels, and the area under the curve (AUC) was calculated (Wang et al. 2014).

PTT: Mice were fasted for 13 h. Sodium pyruvate solution (250 mg/ml) was intraperitoneally injected at a dose of 2 g/kg. Blood samples were collected from the tail tip at 0 min (before sodium pyruvate injection) and 15, 30, 90, and 120 min after injection to measure glucose levels, and the area under the curve (AUC) was calculated (Kinote et al. 2012).

### Histological analysis and follicle counts

Mouse ovaries from 3 animals per group were randomly selected and fixed in 4% paraformaldehyde for a duration of 48 h, after which they were embedded in paraffin, allowing for precise sectioning into 4-μm-thick slices. The sections were stained with hematoxylin and eosin (HE). Follicles were counted in every tenth section, classified based on distinct morphological characteristics to determine their stages, including primordial, primary, secondary, antral, and atretic follicles, as described previously (Myers et al., 2004). Subsequently, the percentage distribution of each follicle type within each ovary was further analyzed. For histology evaluation, the presented images are of whole fields of view.

### Serum hormone detection

The blood samples were placed at room temperature for 2 h, and then centrifuged at 3000 rpm, 4 °C for 15 min. The serum samples were stored at −80 °C refrigerator. The concentrations of FSH, E2 and anti-müllerian hormone (AMH) in serum were detected using enzyme-linked immunosorbent assay kit. Experimental process was carried out according to the manufacturer’s guidelines.

### PAS staining of liver tissues

Paraffin-embedded liver tissues were sectioned, deparaffinized, and hydrated. Sections were stained with periodic acid-Schiff (PAS) reagent B for 10–15 min, rinsed with water, and incubated in PAS reagent A under light-protected conditions for 25–30 min, followed by 5 min of running water washing. Subsequently, sections were counterstained with PAS reagent C for 30 s, differentiated in acidic solution, rinsed, and blued with ammonia water. After dehydration, sections were mounted with neutral resin and visualized under a light microscope. PAS staining was assessed *via* thresholding in ImageJ, the results shown as stained area percentages (Kur et al. 2021). For PAS straining, the presented images are of representative fields of view. The pathological results represent the average of 3 whole fields of view from three different mice.

### Metabolomics analysis

Sixty milligrams of liver tissue pulverized and added 200 µL of methanol/acetonitrile/water (2:2:1, v/v/v). The tissue was homogenized by shaking and then mixed with 800 µL of methanol/acetonitrile/water (2:2:1, v/v/v). The mixture was sonicated in an ice bath for 1 h, followed by incubation at −20 °C for 2 h. The sample was then centrifuged at 16,000 g for 20 min at 4 °C. The supernatant was transferred to a new EP tube and dried under vacuum in a high-speed centrifuge. After drying, 100 µL of methanol-water solution (1:1, v/v) was added, and the sample was centrifuged again at 20,000 g for 20 min at 4 °C.

The UHPLC-MS/MS analysis was conducted using the SHIMADZU-LC30 UHPLC system and QE Plus mass spectrometer (Thermo Scientific). Samples were introduced into an ACQUITY UPLC^®^ HSS T3 column (2.1 mm × 100 mm, 1.8 µm, USA) maintained at a temperature of 40 °C (0.3 mL/min). The mobile phase consisted of 0.1% formic acid in water (A) and acetonitrile (B). The gradient program was as follows: 0–2 min, 0% A; 2–6 min, 48% B; 6–10 min, 100% B; 10–12 min, 100% B; 12–12.1 min, 0% B; 12.1–15 min, 0% B. Then, the QE Plus mass spectrometer was configured with a sheath gas flow rate set at 30 psi, spray voltage of 3.8 kV (+) and 3.2 kV (−), and capillary temperature of 320 °C (±). The acquired raw data were processed through MSDIAL software for peak detection and alignment. Statistical evaluations were conducted utilizing R software (version 4.0.3).

The metabolites were explored using the KEGG (https://www.kegg.jp) database. Differential metabolites were screened based on the following criteria: VIP > 1, p-value < 0.05, and fold change (FC) ≥ 2 or FC ≤ 0.5.

### Western blotting (WB)

Protein is extracted from ovarian tissue, and an appropriate amount of protein lysis buffer was added based on the tissue weight. The protein samples were separated using SDS-PAGE, and after electrophoresis, the proteins were transferred onto a PVDF membrane. Membranes were blocked with 5% skim milk in TBST for 2 h at room temperature to prevent nonspecific binding, followed by overnight incubation at 4 °C with the following primary antibodies: PI3K (1:1000, Proteintech, 20584-1-AP), p-PI3K (1:1000, Cell Signaling Technology, 4228), AKT (1:2000, Cell Signaling Technology, 4691), p-AKT (1:2000, Cell Signaling Technology, 4060), FOXO3a (1:200, Proteintech, 66428-1-Ig), and p-FOXO3a (1:200, Proteintech, 28755-1-AP). The next day, membranes were incubated with horseradish peroxidase (HRP)-conjugated secondary antibodies (1:5000 dilution) for 2 h at room temperature. Protein bands were visualized using ECL plus reagent and imaged with a chemiluminescence detection system. Three mice (*n* = 3) were randomly selected from each group for WB analysis experiments.

### Immunofluorescence staining

Paraffin-embedded ovarian tissue sections were baked at 60 °C for 60 min, followed by deparaffinization. Heat-induced antigen retrieval was performed using a sodium citrate antigen retrieval solution (C1032, Solarbio, Beijing, China). The sections were then blocked with 10% normal donkey serum at room temperature for 30 min. Subsequently, they were incubated overnight at 4 °C with the following specific primary antibodies: PI3K (1:200, 20584-1-AP, proteintech), p-PI3K (1:200, T40116, Abmart), AKT (1:200, 4691, Cell Signaling Technology), p-AKT (1:200, 4060, Cell Signaling Technology), and p-FOXO3a (1:200, 28755-1-AP, proteintech). After primary antibody incubation, sections were incubated with species-appropriate secondary antibodies, and nuclei were counterstained with DAPI. The slides were then protected from light for 30 min before imaging with a confocal microscope. For immunofluorescence experiments the statistical results represent the average of three whole fields of view from three different animals.

### Statistical analysis

Statistical analyses and graphical representations were performed using GraphPad Prism 8 software. All results are expressed as mean ± SEM. Two-tailed Student’s t-test was used to compare the statistical difference between the two groups, while one-way ANOVA analysis was used among multiple comparisons. P values of less than 0.05 were statistically significant.

## Results

### Chemical constituents of WBFC oral liquids

**Figure 1.**
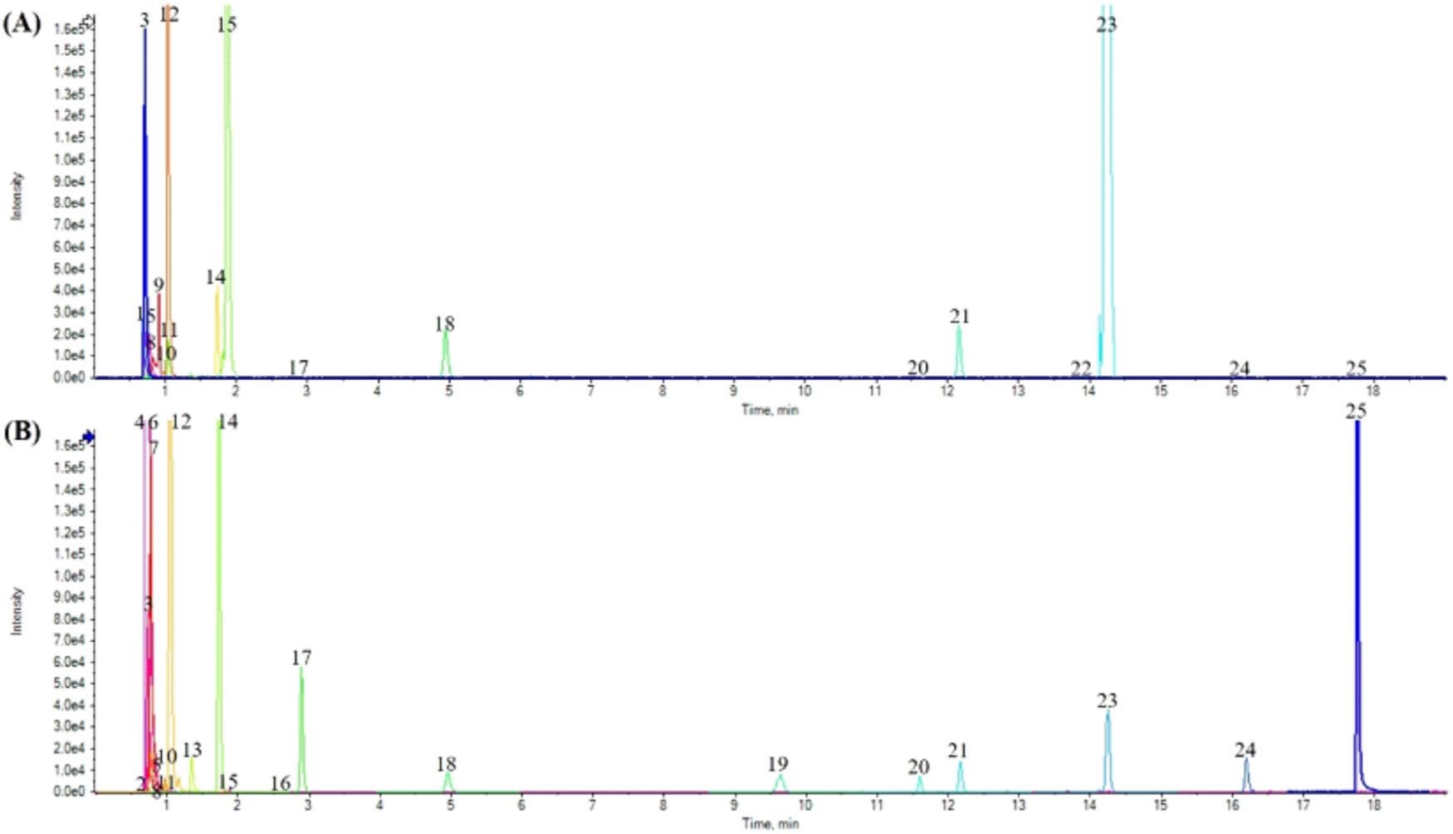
Extracted ion chromatogram of the main chemical components of WBFC under positive ion mode (A) and negative ion mode (B).

To identify the chemical constituents of WBFC oral liquid, the samples were analyzed by UPLC-TOF/MS. A total of twenty-five components in the WBFC were identified by extracting ion chromatography (EIC) and the ion flow chromatography of the superimposed extraction is illustrated in Figure 1. Table 1 provides detailed information on the retention times, molecular formulas, peak intensities, and names of the identified components in WBFC. The analysis demonstrated that the main chemical constituents of WBFC include amino acids, saponins, anthraquinones, and carbohydrates.

**Table 1.** LC-TOF/MS profiling data of 25 characterized compounds in WBFC.

No.	Found RT (min)	Found at mass (Da)	Formular	Adduct	Extraction mass	Error	Intensity	Identification	Source
1	0.69	175.11871	C_6_H_14_N_4_O_2_	[M + H]^+^	175.11895	−1.4	35403	L-Arginine	*Atractylodis macrocephalae* Rhizoma
2	0.71	132.03056	C_4_H_7_NO_4_	[M-H]^-^	132.03023	2.5	2269	Aspartic acid	*Lycii* Fructus
3	0.72	203.05274	C_6_H_12_O_6_	[M + Na]^+^	203.05261	0.7	160387	D-Mannose	*Trionycis* Carapax
	0.73	179.05686		[M-H]^-^	179.05611	4.2	83022		
4	0.72	341.109	C_12_H_22_O_11_	[M-H]^-^	341.10894	0.2	621069	D-(+)-Turanose	Poria
5	0.74	161.04526	C_6_H_10_O_5_	[M-H]^-^	161.04555	−1.8	11263	β-Pachyman	Poria
	0.74	163.05995		[M + H]^+^	163.0601	−0.9	22563		
6	0.77	128.03632	C_5_H_7_NO_3_	[M-H]^-^	128.03532	7.8	177158	L-Pyroglutamic acid	*Lycii* Fructus
7	0.77	191.02045	C_6_H_8_O_7_	[M-H]^-^	191.01973	3.8	147162	Citric acid	*Atractylodis macrocephalae* Rhizoma/ *Lycii* Fructus
8	0.78	182.0811	C_9_H_11_NO_3_	[M + H]^+^	182.08117	−0.4	20189	Tyrosine	*Trionycis* Carapax
	0.79	243.06095		[M + NO3]^-^	243.06116	−0.9	5082		
9	0.91	136.06197	C_5_H_5_N_5_	[M + H]^+^	136.06177	1.5	38706	Adenine	Poria
10	0.92	132.10204	C_6_H_13_NO_2_	[M + H]^+^	132.10191	1	7445	l-Isoleucine	*Trionycis* Carapax
	0.97	130.08847		[M-H]^-^	130.08735	8.6	6703		
11	0.97	166.08607	C_9_H_11_NO_2_	[M + H]^+^	166.08626	−1.1	15661	Phenylalanine	*Trionycis* Carapax
	0.98	164.07176		[M-H]^-^	164.0717	0.4	5138		
12	1.04	375.12933	C_16_H_24_O_10_	[M-H]^-^	375.12967	−0.9	623260	Loganic acid	*Dipsaci* Radix
	1.04	394.171		[M + NH4]^+^	394.1708	0.5	298693		
13	1.35	175.06168	C_7_H_12_O_5_	[M-H]^-^	175.0612	2.7	15751	2-Isopropylmalic acid	*Lycii* Fructus
14	1.73	435.15028	C_17_H_26_O_10_	[M + COOH]^-^	435.1497	1.3	301475	Loganin	*Dipsaci* Radix
	1.74	391.15968		[M + H]^+^	391.15987	−0.5	42669		
15	1.88	357.11941	C_16_H_22_O_9_	[M-H]^-^	357.11911	0.8	4183	Sweroside	*Dipsaci* Radix
	1.88	359.13412		[M + H]^+^	359.13366	1.3	548670		
16	2.62	119.0511	C_8_H_8_O	[M-H]^-^	119.05024	7.2	4050	Phenylacetaldehyde	*Lycii* Fructus
17	2.89	407.13364	C_20_H_22_O_9_	[M + H]^+^	407.13366	0	14793	2,3,5,4′-Tetrahydroxy stilbene-2-O-β-D-glucoside	*Polygoni Multiflori* Radix
	2.9	405.11897		[M-H]^-^	405.11911	−0.3	57999		
18	4.94	447.09249	C_21_H_18_O_11_	[M + H]^+^	447.09219	0.7	22174	Apigenin-7-O-glucuronide	*Atractylodis macrocephalae* Rhizoma
	4.96	445.07725		[M-H]^-^	445.07764	−0.9	9297		
19	9.64	431.09807	C_21_H_20_O_10_	[M-H]^-^	431.09837	−0.7	7892	Emodin 1-O-beta-D-glucoside	*Polygoni Multiflori* Radix
20	11.6	853.27649	C_38_H_48_O_19_	[M + COOH]^-^	853.27609	0.5	7311	Epimedin B	*Atractylodis macrocephalae* Rhizoma
	11.6	809.28597		[M + H]^+^	809.28626	−0.3	10985		
21	12.16	677.24448	C_33_H_40_O_15_	[M + H]^+^	677.244	0.7	24309	Icariin	*Atractylodis macrocephalae* Rhizoma
	12.17	721.23433		[M + COOH]^-^	721.23383	0.7	14023		
22	13.94	118.08671	C_5_H_11_NO_2_	[M + H]^+^	118.08626	3.9	5569	2-(Methylamino)isobutyric acid	*Atractylodis macrocephalae* Rhizoma
23	14.24	927.49631	C_47_H_76_O_18_	[M-H]^-^	927.49589	0.5	38219	Asperosaponin VI	*Dipsaci* Radix
	14.25	946.537		[M + NH4]^+^	946.537	0	1698481		
24	16.2	821.39664	C_42_H_62_O_16_	[M-H]^-^	821.39651	0.2	15792	Glycyrrhizin	*Atractylodis macrocephalae* Rhizoma
	16.2	823.40989		[M + H]^+^	823.41106	−1.4	5341		
25	17.76	269.04587	C_15_H_10_O_5_	[M-H]^-^	269.04555	1.2	340982	Emodin	*Polygoni Multiflori* Radix

### Network pharmacology analysis for predicting the potential mechanisms of WBFC in POI treatment

**Figure 2.**
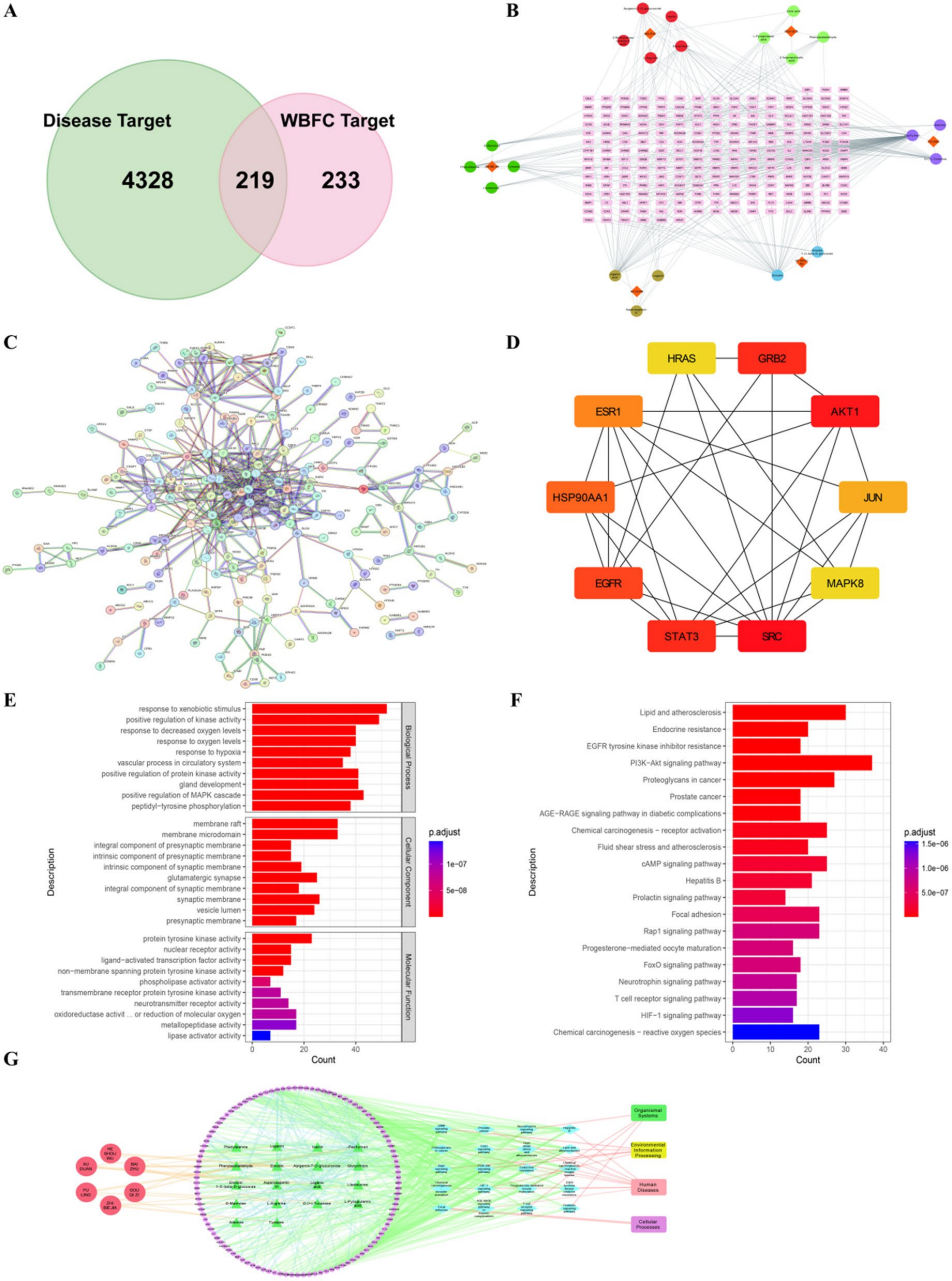
Network pharmacology analysis for predicting the potential mechanisms of WBFC in the treatment of POI. (A) Venn diagram of overlapping targets between WBFC and POI. (B) Component-target network of WBFC. Diamonds represent the traditional Chinese medicine components of WBFC, circles represent active ingredients, and squares represent predicted targets. (C) PPI (protein–protein interaction) network of potential targets for WBFC in the treatment of POI. (D) Top 10 hub genes screened based on degree value from the PPI network. (E) GO enrichment results of WBFC action targets. (F) KEGG pathway enrichment results of WBFC action targets. (G) Network diagram of herbs in WBFC-compounds-targets. The red circles represent herbs in WBFC, the green triangles represent compounds, and the pink circles represent drug targets. The blue diamonds represent signaling pathways, and the rectangular nodes represent annotation information for signaling pathways in the network. The edges indicate the relationships between these nodes.

A total of 452 drug targets were obtained from the Swiss and PharmMapper databases (Supplemental Table 3). Using the OMIM, GeneCards, DisGeNET, and DrugBank databases, 4547 potential disease targets for POI were identified (Supplemental Table 4). By integrating the disease targets with the potential targets of WBFC, a Venn diagram was created, ultimately identifying 219 potential targets for WBFC in the treatment of POI, based on which a Component–Targets interaction network was subsequently established. (Figure 2(A,B)). Additionally, the minimum required interaction score was set to 0.9, and unconnected nodes in the network were hidden, a PPI network analysis was constructed based on the 219 overlapping targets using the String platform (Figure 2(C)). Key gene targets were identified using CytoHubba, and the top 10 hub genes, including SRC, AKT1, GRB2, STAT3, EGFR, HSP90AA1, ESR1, JUN, MAPK8, and HRAS, were selected based on degree values and considered as key targets for WBFC in the treatment of POI (Figure 2(D)). A total of 219 common targets were used as the data basis for GO and KEGG enrichment analyses. According to the enrichment results for biological processes, the targets of WBFC in the treatment of POI were mainly involved in responses to xenobiotic stimulus and positive regulation of kinase activity. Cellular-component enrichment indicated that these targets act primarily at sites such as the membrane raft, while molecular-function analysis showed a close association with protein tyrosine kinase activity (Figure 2(E)).

KEGG pathway enrichment analysis revealed that numerous targets were significantly enriched in the PI3K-AKT signaling pathway, epidermal growth factor receptor tyrosine kinase inhibitor resistance, AGE-RAGE signaling pathway in diabetic complications, and FOXO signaling pathway (Figure 2(F)). Using the p-value as the screening criterion, the top 20 significantly enriched KEGG pathways were selected as the dataset, a WBFC-Compounds-Targets interaction network was accordingly constructed (Figure 2(G)). Remarkably, the PI3K-AKT pathway exhibited the highest degree of enrichment. Additionally, the PPI network also showed that AKT1 was among the top ten key genes involved in WBFC against POI. FOXO plays a crucial role in the selective activation of primordial follicles, while FOXO3 functions as a PI3K-dependent molecular switch that controls the initiation of oocyte growth (John et al. 2008). Based on the above results and relative literature, PI3K/AKT/FOXO signaling would be the key targets involved in the effect of WBFC for POI management.

### WBFC improved ovarian tissue morphology and ovarian function in 4-VCD induced POI mice

**Figure 3.**
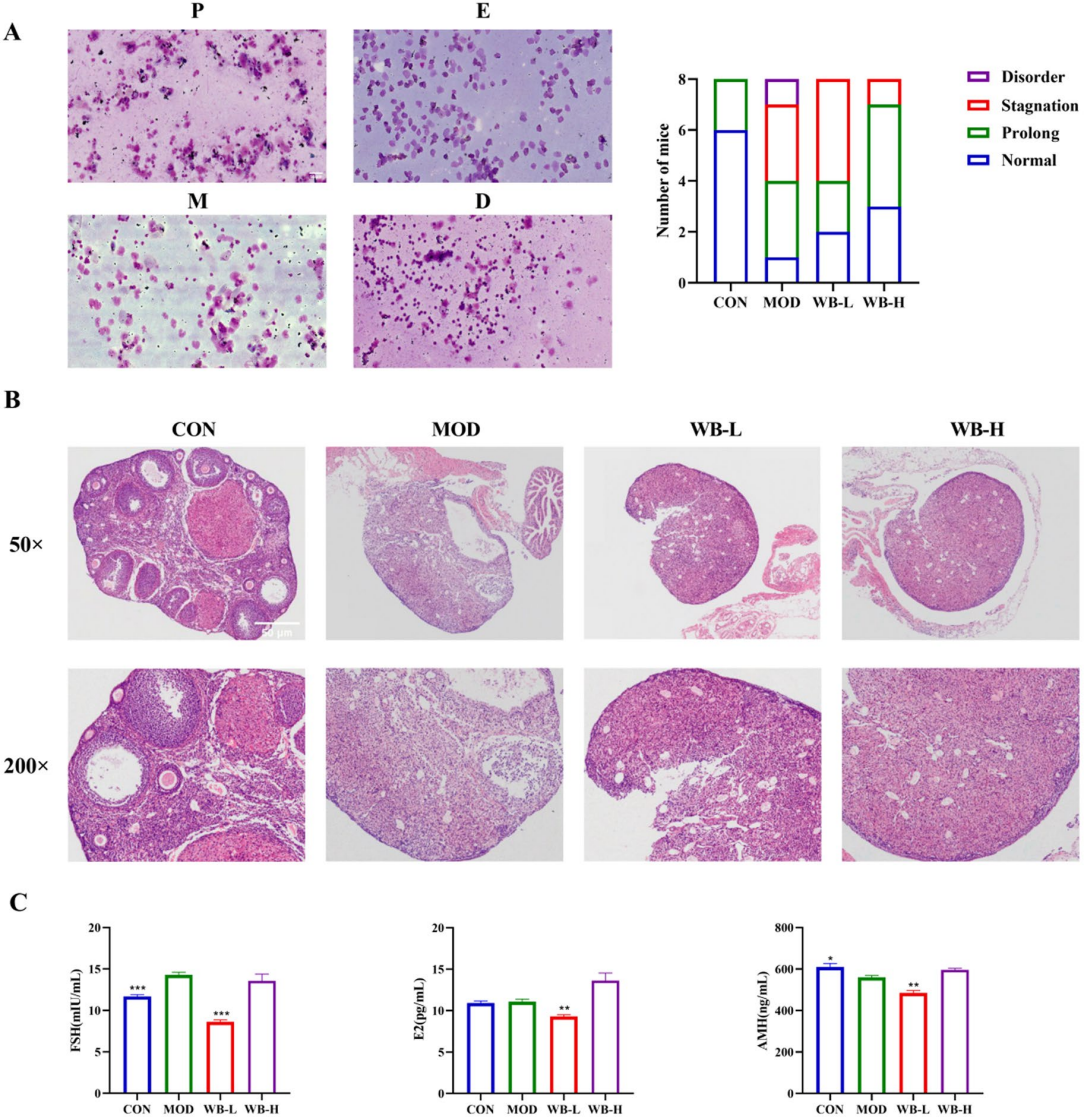
Effects of WBFC on the histomorphology and ovarian function in 4-VCD-induced POI mice. (A) Representative estrous cycle stages in mice and the number of mice in each cycle stage. Proestrus (P), estrus (E), metestrus (M), and diestrus (D). Scale bar represents 50 μm. (B) Ovarian tissues of mice stained with HE and observed under 50×, and 200× magnification (*n* = 3). (C) Changes in hormone levels of FSH, E2, and AMH in mice (*n* = 6). Compared with the model group, *p < 0.05, **p < 0.01, ***p < 0.001.

As shown in Figure 3(A), the estrous cycles of mice were normal with the sequence of stages being proestrus, estrus, metestrus, and diestrus in control group, while predominantly characterized by disruption or prolongation in 4-VCD mice. The 4-VCD mice progressively reestablished more regular cyclicity after WBFC treatment. HE staining revealed that the control mice were in normal morphology by characterizing with follicles and corpus luteum of different sizes at different stages of development. 4-VCD caused destruction of primordial and primary follicles in mice, which is consistent with previous report (Kappeler and Hoyer 2012). WBFC could substantially increase the number of primordial follicles (Figure 3(B)). Serum levels of FSH, E2, and AMH were measured to investigate the efficacy of WBFC. Figure 3(C) demonstrated that POI mice exhibited a significant increase in FSH levels and a marked decrease in AMH levels compared to control mice. The WBFC-treated mice showed a reduction in FSH levels, with the WB-L group demonstrating a statistically significant decrease (*p* < 0.001) when compared to the MOD group. Collectively, WBFC ameliorated estrous cycle irregularities, ovarian histopathological damage, and endocrine dysfunction in 4-VCD-induced POI mice.

### WBFC activated PI3K/AKT/FOXO3 signaling pathway in ovaries of POI model mice

**Figure 4.**
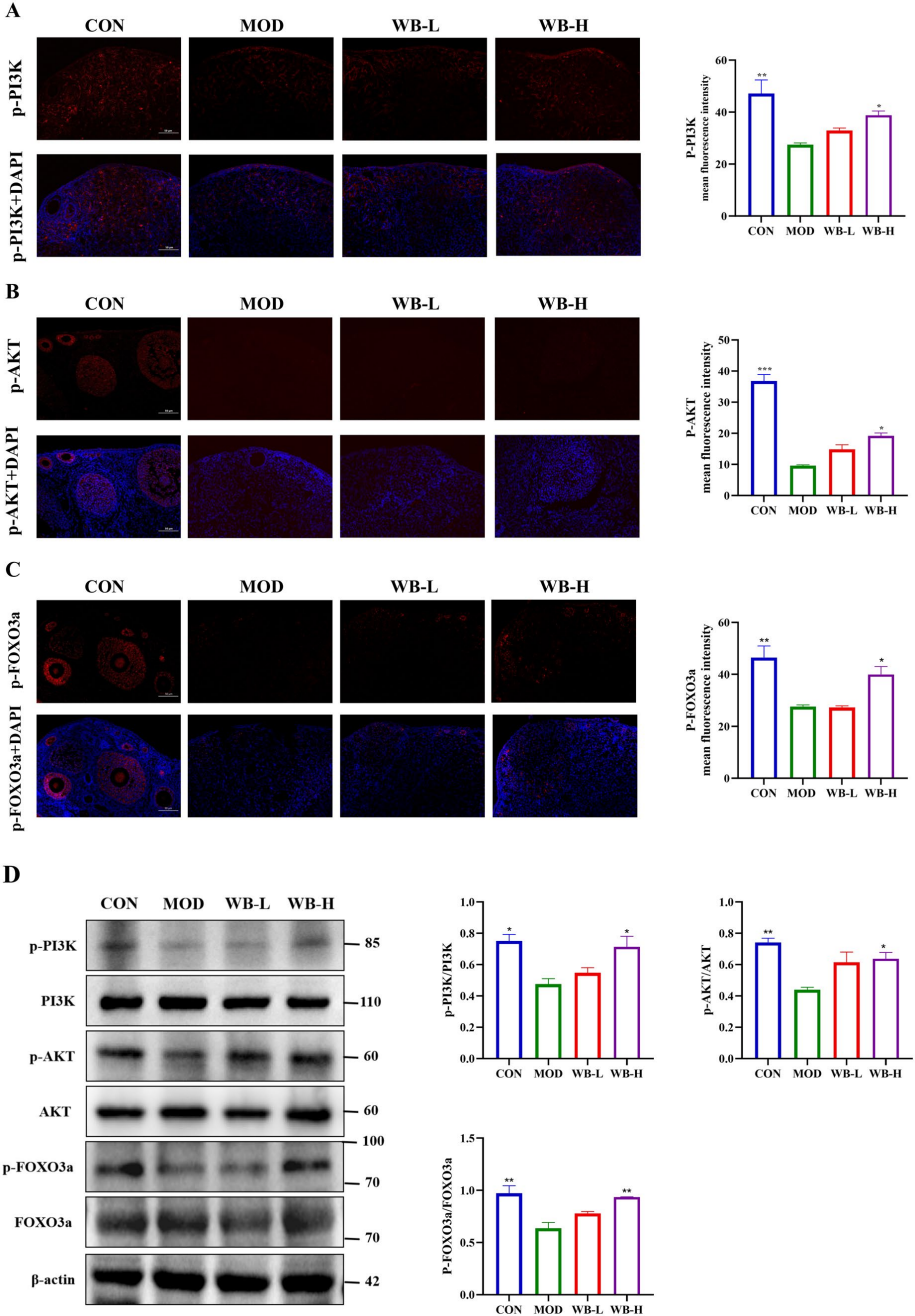
WBFC regulates ovarian function and activates PI3K/AKT/FOXO3a signaling in POI mice. Immunofluorescence and quantification of (A) p-PI3K, (B) p-AKT, and (C) p-FOXO3a in ovaries. (D) Western blot analysis of protein expression levels and semi-quantitative results of p-PI3K/PI3K, p-AKT/AKT, and p-FOXO3a/FOXO3a in ovarian tissues of POI mice treated with WBFC (*n* = 3). Compared with the model group, *p < 0.05, **p < 0.01, ***p < 0.001. Scale bar represents 50 μm.

The representative protein markers of PI3K/AKT/FOXO3 pathway were assessed using immunofluorescence and western blot methods. Compared to the control mice, the phosphorylation levels of PI3K and AKT were significantly decreased in 4-VCD induced mice. WBFC supplementation dose-dependently upregulated the levels of phosphorylated PI3K and AKT (Figure 4(A,B,D)). Phosphorylated AKT localized predominantly in oocytes, with WBFC enhancing its expression in primordial/primary follicles, suggesting its potential role in promoting follicular development through AKT activation. To elucidate the downstream effects of WBFC on FOXO3a, western blot analysis demonstrated a significantly reduced p-FOXO3a/FOXO3a ratio in POI mice compared to controls, which was reversed by WBFC treatment in a dose-dependent manner (Figure 4(D)). Immunofluorescence results also showed that, compared to the CON group, the expression of p-FOXO3a was markedly decreased in the MOD group, while the expression levels of p-FOXO3a were significantly elevated in both the WB-L and WB-H groups (Figure 4(C)). These results demonstrated that WBFC protected against 4-VCD induced mice and modulated PI3K/AKT/FOXO3 signaling pathway.

### Metabolic recovery in POI mice by WBFC intervention

### Regulating glucose metabolism and improving glycogen accumulation in POI mice by WBFC intervention

**Figure 5.**
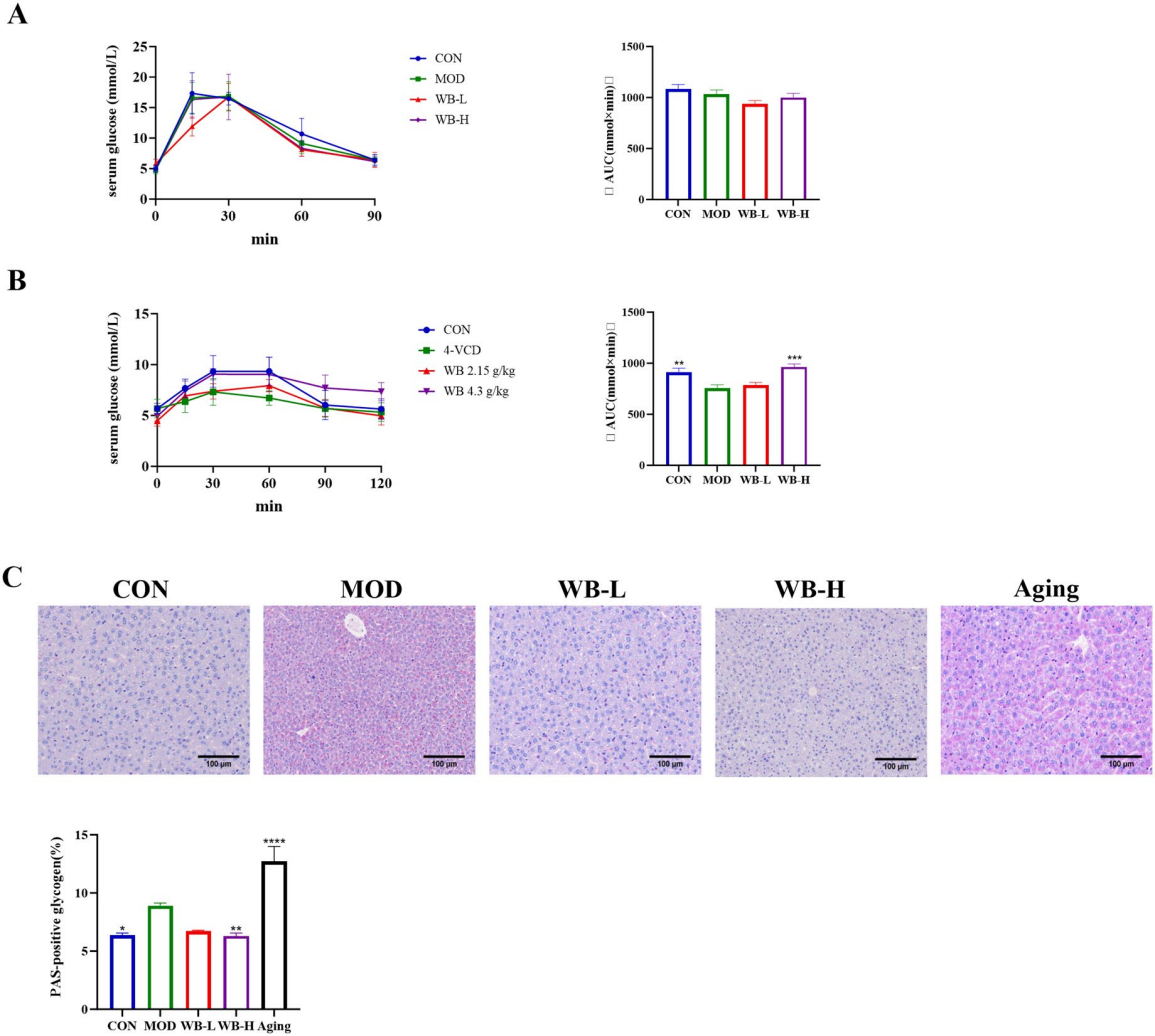
WBFC regulates glucose metabolism in POI mice. (A) OGTT curve. (B) PTT curve. (C) PAS staining. Compared with the model group, *p < 0.05, **p < 0.01, ***p < 0.001, ****p < 0.0001.

As illustrated in Figure 5(A), the OGTT results indicated no statistically significant differences in blood glucose levels between the control and POI mice. In contrast, the PTT curve in the model group showed a significant decrease compared to the control group, which was dose-dependently ameliorated by WBFC administration (Figure 5(B)). These findings suggest that WBFC effectively restored impaired hepatic glucose metabolism in 4-VCD-induced POI mice. PAS staining of liver tissues revealed abnormal hepatic glycogen metabolism in both 4-VCD-induced POI mice and naturally aging mice compared to controls. Notably, the MOD group exhibited markedly enhanced PAS staining (*p* < 0.01), reflecting excessive glycogen accumulation under POI conditions. WBFC treatment significantly attenuated hepatic glycogen deposition, bringing it closer to physiological levels (Figure 5(C)). Previous studies have established that impaired glycogen synthesis contributes substantially to insulin resistance (Turnbull et al. 2013). Collectively, these preliminary findings demonstrate that POI disrupts glucose metabolism and exacerbates insulin resistance, whereas WBFC intervention mitigates these pathological alterations.

#### Liver metabolome revealed a dramatic reduction of glutamine and inositol lipid metabolites in 4-VCD induced POI mice, which could be recovered by the supplement of WBFC

**Figure 6.**
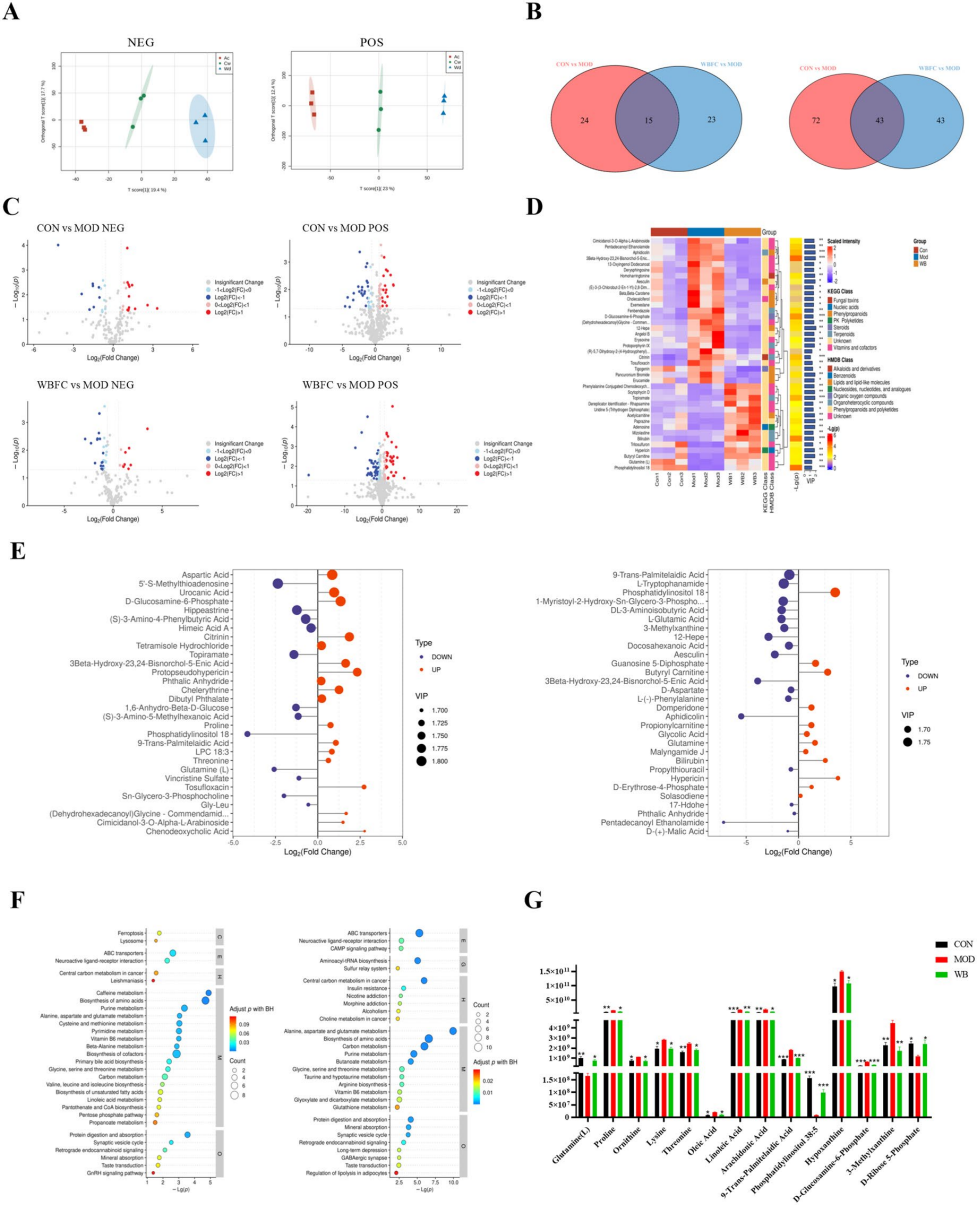
Untargeted metabolomics analysis of changes in metabolites in each group after intervention with WBFC. (A) Schematic diagram of principal component analysis (PCA) results in positive and negative ion modes for each group. (B) Venn analysis of differential metabolites between comparison groups. (C) Volcano plot of differential metabolites. (D) Hierarchical clustering results of differential metabolites between comparison groups. (E) Bar chart of differential metabolites between comparison groups. Blue and red dots represent downregulated and upregulated differential metabolites, respectively. The size of the dots indicates the VIP value, the larger the dot, the higher the VIP value, indicating greater importance of the variable. (F) KEGG pathway enrichment bubble chart (top 30) of differential metabolites in each group. (G) Screening of key differential metabolites between groups. Compared with the model group, *p < 0.05, **p < 0.01, ***p < 0.001.

**Figure 7.**
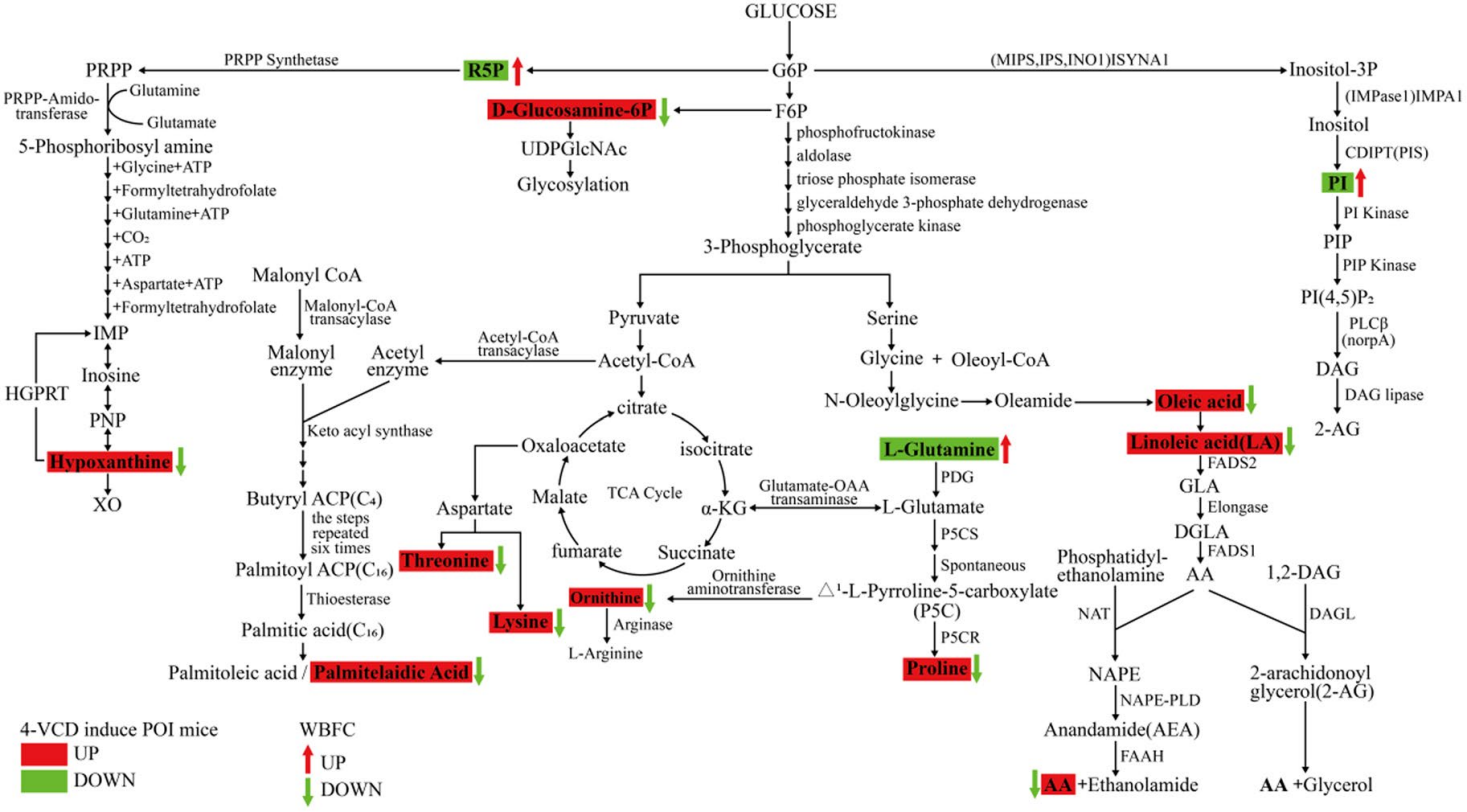
Prediction of altered major metabolic pathways in POI mice and the interventional effects of WBFC.

Accumulating evidence suggest that POI patients are predisposed to disorders of lipid and glucose metabolism (Podfigurna et al. 2018; Yu et al. 2023; Zhou et al. 2023; Chen et al. 2025). According to TCM Theory, liver and kidney dysfunction are considered pathogenesis for POI. It has been reported that the metabolic profiles of liver and kidney in cisplatin-induced POI mice were changed and closely associated with ovarian function (Chen et al. 2022). As such, our study utilized untargeted metabolomics to systematically investigate the shifts of hepatic metabolism in POI mice induced by 4-VCD and the influence of WBFC on it in the POI model mice. The OPLS-DA plot revealed a clear demarcation between the CON group, MOD group, and WBFC group under both positive and negative ion modes, indicating significant differences in their untargeted hepatic metabolomic profiles. Candidate metabolites with significant changes (*p* < 0.05) were identified as potential biomarkers through variance analysis (Figure 6(A,B)). The selected differential metabolites were visualized using volcano plots. In the negative ion mode, the MOD group exhibited increased levels of 21 metabolites and decreased levels of 18 metabolites compared with the control group. The WBFC group exhibited increased 9 metabolites and 26 metabolites compared with the MOD group (Figure 6(C,D)). Similarly, in the positive ion mode, 45 metabolites were upregulated and 41 were significantly downregulated in the MOD group compared to the control group. Moreover, 55 metabolites were upregulated and 65 were significantly downregulated in the WBFC group compared to the MOD group. It should be noted that among the top 30 differential metabolites, ranked by VIP value, four overlapped metabolites (phosphoinositol 38:5, glutamine, trans-palmitelaidic acid, and 3beta-hydroxy-23,24-bisnorchol-5-enic acid) were identified. As shown in Figure 6(E), phosphoinositol 38:5 and glutamine were decreased markedly in POI mice compared with control mice, while WBFC treatment was found markedly increased compared with MOD group. Trans-palmitelaidic acid and 3beta-hydroxy-23,24-bisnorchol-5-enic acid were increased significantly in POI mice in comparison with the CON group while decreased significantly after WBFC treatment. KEGG pathway enrichment analysis of the differential metabolites demonstrated the differentially expressed metabolites mainly occurred in the alanine, aspartate, and glutamate metabolism pathway, the biosynthesis of amino acid pathway, the carbon metabolism pathway, and the purine metabolism pathway (Figure 6(F)). Statistical analysis of the differential metabolites according to VIP values was conducted among CON, MOD and WBFC groups, and results indicated that 14 differentially expressed metabolites were identified, all of which are related to glucose and lipid metabolism. As shown in Figure 6(G), in POI mice the biosynthesis of four amino acids including proline, lysine, threonine, and ornithine significantly increased. The content of hypoxanthine significantly increased and D-Ribose 5-phosphate decreased as participating in the biosynthesis of purine. Of note, both of these two synthetic pathways require the mobilization of GLN, resulting in a sharply decrease in GLN content in the POI model mice. Fatty acids, including palmitelaidic acid, oleic acid, linoleic acid, and arachidonic acid , significantly increased, with linoleic acid and arachidonic acid having higher relative contents and palmitelaidic acid having a highest VIP value. Remarkably, the content of PI synthesis significantly decreased, with the largest fold change between the MOD group and CON group. The use of WBFC were able to restore the levels of all metabolites above and repair the metabolic abnormalities (Figure 7).

#### PI and GLN supplement improved ovarian function and activated PI3K/AKT signaling pathway in 4-VCD induced POI mice

**Figure 8.**
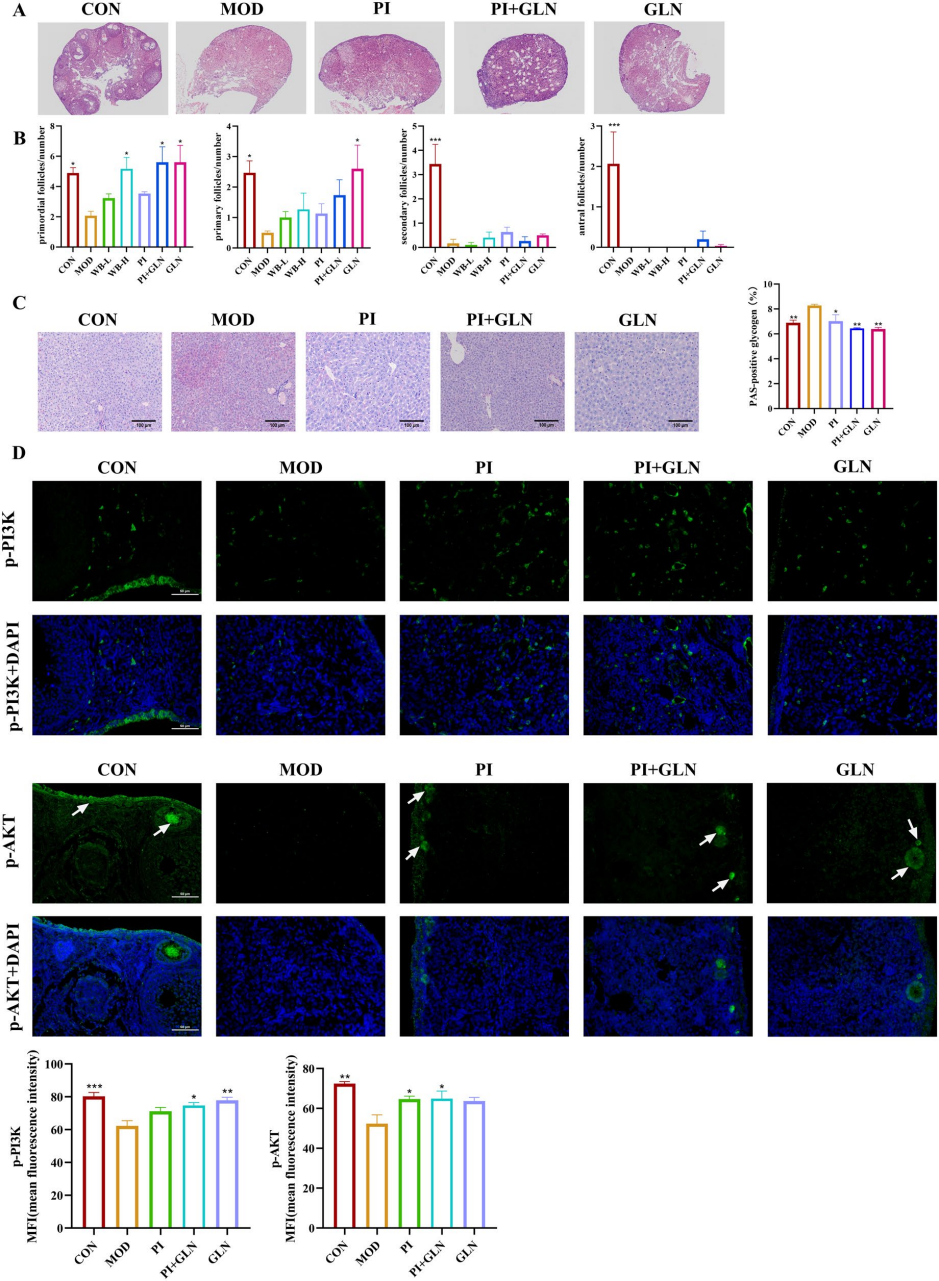
PI and GLN improve ovarian tissue morphology and ovarian function in 4-VCD induced POI mice. (A) HE-stained histopathological sections of ovarian tissue (400×) with a scale bar of 50 μm. (B) Statistics on the number of primordial follicles, primary follicles, secondary follicles, and sinus follicles of mice in each group. Compared with the model group, *p < 0.05, **p < 0.01, ***p < 0.001. (C) PAS staining results of mouse liver tissue in each group. (D) Immunofluorescence analysis of the effect of on protein expression in ovarian tissue of POI mice: P-PI3K, P-AKT. Mean values are presented with ± SEM, *n* = 3. Compared with the model group, *p < 0.05, **p < 0.01, ***p < 0.001.

The putatively identified ‘key metabolites’ (including PI and GLN) have been confirmed with MS/MS data (Supplemental Figure 1). In the CON group, the ovarian tissue structure was intact, healthy follicles at all levels were visible, and mature follicles were well developed. 4-VCD-induced POI mice had few healthy follicles at all levels (Figure 8(A)). Compared to the MOD group, the ovarian cortical integrity in the PI group, PI+GLN group, and GLN group showed varying degrees of recovery, with the PI+GLN group demonstrating the most significant improvement. Following the administration of PI and GLN, the number of healthy follicles also increased compared to the MOD group (Figure 8(B)). PAS staining was significantly intensified in the hepatic tissue of mice in the 4-VCD-induced POI model group in comparison with the model group, liver glycogen staining was reduced in the PI group, the GLN group and the group co-administered with both, suggesting that PI and GLN can ameliorate the metabolic abnormalities associated with POI (Figure 8(C)). As shown in the Figure 8(D), compared with the MOD group, the expression levels of p-PI3K and p-AKT were differentially up-regulated in the PI, GLN, and PI+GLN groups, and p-AKT expression was similarly concentrated in the oocytes of Primordial Follicles (PMFs) and Primary Follicle (PFs), suggesting that the activation of p-AKT promotes follicular activation, and that the PI and GLN may protect the follicle survival and development by regulating the PI3K/AKT pathway. These findings suggest that combined use of PI and GLN, two WBFC-inducible metabolites in POI promote the development of follicles in POI mice and modulated the PI3K/AKT pathway.

## Discussion

The pronounced etiological heterogeneity of POI (Ke et al. 2023) is reflected in the divergent molecular manifestations observed across different animal models. Notably, the PI3K/AKT/FOXO3a signaling pathway demonstrates paradoxical regulation patterns in distinct POI models. Chemotherapy-induced POI models show upregulation of phosphorylated PI3K, AKT, and FOXO3a proteins in ovarian tissue (Zheng et al. 2022; Su et al. 2023). Conversely, in 4-VCD induced models, including our experimental findings and independent literature reports (Cakir et al. 2024; Zhang et al. 2024) these phosphorylated signaling molecules exhibit significant downregulation. This apparent contradiction in pathway activation states likely reflects the dual mechanistic origins of follicular depletion – both excessive primordial follicle activation and impaired follicular recruitment have been implicated in POI pathogenesis. Our study utilized the 4-VCD induced murine model to assess the therapeutic efficacy of the WBFC formula in the context of POI. The investigation was motivated by clinical observations showing diminished primordial follicle activation potential in POI patients exhibiting kidney-liver deficiency patterns in TCM diagnostics. The 4-VCD-induced POI model was selected based on its well-documented ability to selectively deplete small pre-antral follicles (primordial and primary stages) (Abolaji et al. 2016), thereby closely recapitulating the pathogenic mechanisms and histopathological alterations observed in human POI. Moreover, we performed fingerprint profiling and quantitative analysis of the index components for WBFC used in this study (Supplemental Figure 2 and Table 5).

Network pharmacological analysis identified the PI3K/AKT/FOXO signaling pathway as the primary therapeutic target of WBFC formula in treating POI. This pathway plays a well-established, critical role in follicular activation, development, and ovarian function maintenance. During early folliculogenesis, before FSH receptor expression, primordial follicle growth depends on KIT ligand (KITL)-KIT receptor interactions between oocytes and pre-granulosa cells. The PI3K pathway serves as a crucial downstream mechanism of KIT-KITL signaling. Following KITL stimulation, PI3K pathway activation in primordial follicles triggers sequential phosphorylation of Akt (protein kinase B) and FOXO3a. This signaling cascade primarily converges at 3-phosphoinositide-dependent kinase 1 (PDK1), which phosphorylates AKT at Thr308 to activate it (Reddy et al. 2010). Notably, insufficient PI3K substrates may impair AKT phosphorylation. Once activated, phosphorylated AKT induces FOXO3a phosphorylation in granulosa cells, thereby suppressing FOXO3a-mediated transcription of pro-apoptotic genes. This process reduces granulosa cell apoptosis and maintains follicular integrity. FOXO transcription factors, particularly FOXO3, function as PI3K-dependent molecular switches that regulate primordial follicle activation and oocyte growth initiation (John et al. 2008). Our experimental results align with previous findings (Zhang et al. 2024), showing significantly reduced levels of phosphorylated PI3K, AKT, and FOXO3a in 4-VCD-induced POI mice. Importantly, WBFC treatment dose-dependently restored the phosphorylation levels of these key pathway components, suggesting its therapeutic effect might involve PI3K/AKT/FOXO3 pathway activation.

POI exhibits a phenomenon characterized by the coexistence of reproductive dysfunction and covert metabolic alterations. As early as 2018, a clinical study involving 176 adult women (98 POI patients and 78 healthy controls) found no significant difference in the insulin resistance index between the two groups. However, during the oral glucose tolerance test, POI patients showed significantly increased insulin secretion at 0 min and 60 min (Kunicki et al. 2018). In 2022, a meta-analysis incorporating 21 studies, with 1,573 POI women and 1,762 control women, revealed that POI patients had significantly higher waist circumference, total cholesterol, and fasting blood glucose levels. Additionally, POI patients exhibited slightly higher insulin levels (Podfigurna et al. 2018). In animal models of chemotherapy-induced POI, mice displayed abnormalities in lipid metabolism and amino acid metabolism (Ling et al. 2019). In the mouse model induced by 4-VCD combined with a high-fat diet, researchers observed that the experimental group developed impaired glucose tolerance as early as week 12, which was 4 weeks earlier than the control group on the same diet (Kappeler and Hoyer 2012). A 2023 study published in Nature Communications demonstrated that follicle-stimulating hormone signaling in the pituitary gland regulates hepatic lipid metabolism in mice (Qiao et al. 2023). This study employed untargeted metabolomics analysis of liver tissue to investigate hepatic metabolic alterations in POI model mice and assess the regulatory effects of WBFC intervention. The differential metabolites from the control group/model group and the model group/WBFC formula group were ranked based on their VIP values. Among the top 30 differential metabolites, PI and GLN were identified as overlapping compounds and decreased significantly in 4-VCD induced POI mice. WBFC Formula could restore the aforementioned metabolic impairments. Therefore, we identified PI and GLN as key metabolites regulated by WBFC and separately validated their therapeutic effects and mechanisms in the treatment of POI.

As a key component of cell membranes and a direct substrate of PI3K, phosphatidylinositol is crucial for maintaining ovarian function, and moderate activation of PI3K contributes to the long-term survival of primordial follicles. Additionally, PI3K plays an important role in regulating intracellular vesicle trafficking, particularly in mediating lipid transport. When the PI3K pathway is inhibited, lipid transport efficiency decreases, which may restrict steroid hormone synthesis and further exacerbate the ovarian microenvironment, accelerating the pathological progression of POI (Luo et al. 2019). GLN, the most abundant amino acid in the body, is widely involved in various critical physiological processes, including protein synthesis, energy metabolism, immune regulation, and antioxidant responses. Energy metabolism is essential for the normal development and maturation of oocytes, influencing nutrient uptake, energy production, biosynthesis, and redox balance (Bao et al. 2024). GLN helps stabilize the granulosa cell layer, maintaining follicular structural integrity by inhibiting granulosa cell apoptosis. It also provides nutritional support for oocyte development and promotes follicular maturation and ovulation (Bao et al. 2024). As a key metabolite in the TCA cycle, GLN supplementation supplies energy for oocyte growth and development. Meanwhile, AKT-induced phosphorylation of FOXO3a might suppress apoptosis, a process that also requires energy, and GLN can provide the necessary energy to support the ovary in initiating anti-apoptotic mechanisms. Our findings revealed that the combination of PI and GLN exerted pronounced effects on primordial follicle development and AKT activation compared to 4-VCD model mice, suggesting that PI and GLN might serve as key metabolic mediators underlying the therapeutic benefits of WBFC in POI treatment. The selection of intraperitoneal injection for GLN and intravenous injection for PI was based on the following rationale: although intraperitoneal administration of glutamine has not been specifically reported in studies of POI, it has been demonstrated in the context of polycystic ovary syndrome (PCOS) that intraperitoneal injection of glutamine can significantly and effectively alleviate inflammation and oxidative stress associated with PCOS (Wu et al., 2020). For PI, intravenous injection was chosen due to its high bioavailability and rapid attainment of effective blood concentration, which is supported by precedents in similar animal experiments (Shimizu et al. 2010). Furthermore, previous studies evaluating the efficacy of a TrkB agonist antibody (Qin, 2022) or mesenchymal stem cells (Shin, 2021; Sun, 2013) in POI animal models also utilized intravenous injection as the administration route.

LC-TOF-MS analysis of WBFC oral liquid revealed that its amino acid components – including L-arginine, aspartic acid, pyroglutamic acid, isoleucine, phenylalanine, and tyrosine – are primarily derived from Biejia (Carapax Trionycis), the monarch drug of the formulation. L-Arginine and aspartic acid are closely associated with PI synthesis. L-Arginine promotes G-6-P generation and enhances insulin secretion (Cho et al. 2020). Aspartic acid contributes a nitrogen atom during inositol biosynthesis, serving as a precursor for purine alkaloids. Moreover, these amino acids exhibit direct metabolic relationships with glutamine. L-Arginine can be converted to other proteinogenic amino acids, including glutamic acid. Hepatic L-aspartic acid synthesis predominantly occurs *via* mitochondrial aspartate aminotransferase, utilizing oxaloacetate and glutamate generated through glutamine deamidation. L-Pyroglutamic acid, a non-essential metabolite, is derived from glutamine and glutamic acid. Isoleucine plays a critical role in branched-chain amino acid (BCAA) catabolism, facilitating glutamine and alanine synthesis (Gelberman et al. 1975). Phenylalanine, an essential amino acid that can be converted to tyrosine, is ultimately degraded into fumarate through a process that depends on glutamine-supported oxidative TCA cycle activity (Sullivan et al. 2013). While qualitative profiling has identified these components, their absolute concentrations remain unquantified. Subsequent studies should employ targeted amino acid metabolomics to precisely measure these metabolites in both WBFC oral liquid and relevant biological matrices, enabling pharmacokinetic and functional characterization.

Phosphoinositides are key molecular mediators of the anabolic functions of insulin and thus central regulators of metabolism (Bridges and Saltiel 2015). Oral glutamine supplementation can increase circulating insulin levels in lean, obese, and type 2 diabetic subjects (Greenfield et al. 2009). In a study of 90 patients with POI, Knauff et al. (2008), identified subtle but significant alterations in blood lipid profiles, including elevated triglyceride (TG) levels and borderline low HDL levels – a pattern frequently associated with insulin resistance. POI patients demonstrate significantly higher 1-hour postprandial insulin levels, reflecting impaired peripheral insulin sensitivity or developing insulin resistance (Kunicki et al. 2018). Ovarian insufficiency disrupts insulin secretion in response to glucose *via* hypothalamic de novo ceramide synthesis activation, as reported (Meneyrol et al. 2021). Hepatic glycogen metabolism is precisely regulated by insulin, which maintains glycogen homeostasis by suppressing glycogenolysis while promoting glycogenesis. Intriguingly, our study revealed substantial glycogen accumulation in the liver tissues of both 4-VCD induced POI mice and naturally aged mice. Notably, abnormal hepatic glycogen accumulation is prevalent in galactosemia patients, and female patients with this disorder often develop POI as a comorbidity (Hagen-Lillevik et al. 2021). Emerging evidence highlights the role of metabolic dysregulation, particularly insulin resistance, in mediating inter-organ crosstalk (Tsutsumi et al. 2023). Liver-derived exosomes have been shown to orchestrate inter-organ communication and enhance systemic glucose homeostasis by modulating peripheral tissues *via* endocrine signaling (Miotto et al. 2024). Furthermore, multiple studies have established a hepato-ovarian axis linking polycystic ovary syndrome (PCOS) and nonalcoholic fatty liver disease (NAFLD) (Targher et al. 2016; Liu et al. 2023; Lan et al. 2026). However, direct evidence linking POI to causal alterations in hepatic lipid or glycogen metabolism was lacking, despite the known role of nutrient sensing dysregulation in aging. Our findings demonstrate that WBFC restores the liver metabolome, thereby rescuing POI in mice. This work provides novel insights into the role of inter-organ communication in nutrient metabolism during POI, offering a potential mechanistic link between hepatic metabolic dysfunction and ovarian insufficiency.

We recognize the following limitation in our current work: while we have utilized network pharmacology and enrichment outputs to construct a mechanistic framework, this in silico evidence alone is insufficient for definitive conclusions. Such analyses serve well for hypothesis generation but require experimental validation. Consequently, establishing causality will be essential in subsequent research through approaches including pathway perturbation (inhibition/knockdown) with functional rescue, comprehensive dose-response evaluations, and experiments demonstrating phenotype abolition upon pathway blockade.

## Conclusions

**Figure 9.**
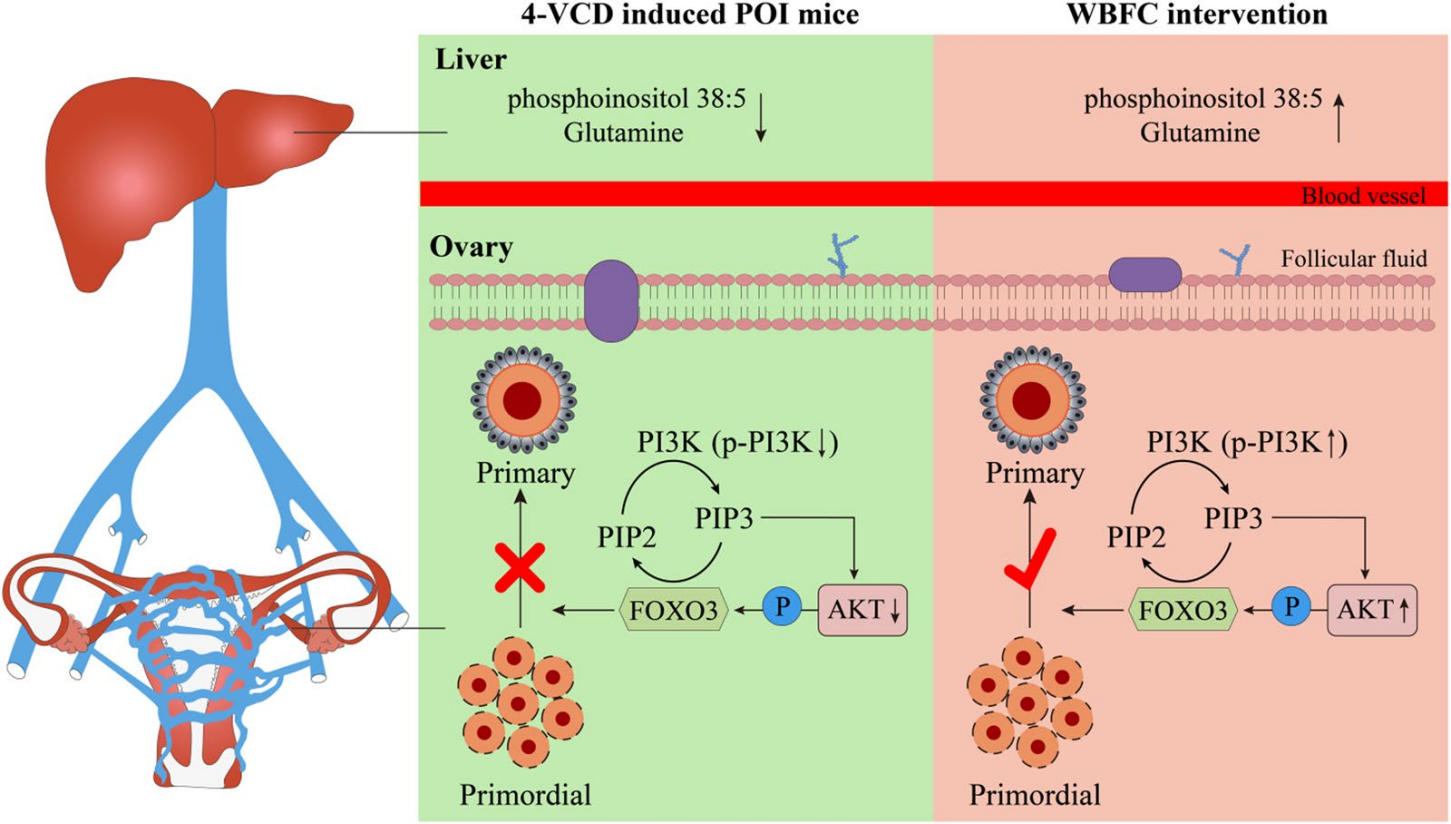
Schematic representation of the proposed mechanism of WBFC formula-inducible metabolites in POI model mice that facilitate ovarian renovation.

The primary bioactive constituents of WBFC Formula include amino acids, saponins, anthraquinones, and carbohydrates. Mechanistic studies demonstrate that WBFC effectively ameliorates ovarian dysfunction and activated the PI3K/AKT/FOXO3a signaling pathway in POI mice. Furthermore, WBFC intervention significantly modulates metabolic homeostasis in POI mice, with PI and GLN emerging as potential key mediators of its therapeutic efficacy against POI (Figure 9).

## Supplementary Material

Supplemental Figure 1.pdf

Supplemental Data.docx

## Data Availability

All data used during the current study are available from the corresponding author upon reasonable request.

## References

[CIT0001] Abdulhafiz F et al. 2022. LC-TOF-MS/MS and GC-MS based phytochemical profiling and evaluation of wound healing activity of *Oroxylum indicum* (L.) Kurz (Beka). Front Pharmacol. 13:1050453. 10.3389/fphar.2022.1050453 36483735 PMC9723245

[CIT0002] Abolaji AO et al. 2016. Evidence of oxidative damage and reproductive dysfunction accompanying 4-vinylcyclohexene diepoxide exposure in female Wistar rats. Reprod Toxicol. 66:10–19. 10.1016/j.reprotox.2016.09.009 27647594

[CIT0003] Armeni E, Paschou SA, Goulis DG, Lambrinoudaki I. 2021. Hormone therapy regimens for managing the menopause and premature ovarian insufficiency. Best Pract Res Clin Endocrinol Metab. 35(6):101561. 10.1016/j.beem.2021.101561 34274232

[CIT0004] Bao S, Yin T, Liu S. 2024. Ovarian aging: energy metabolism of oocytes. J Ovarian Res. 17(1):118. 10.1186/s13048-024-01427-y 38822408 PMC11141068

[CIT0005] Bridges D, Saltiel AR. 2015. Phosphoinositides: key modulators of energy metabolism. Biochim Biophys Acta. 1851(6):857–866. 10.1016/j.bbalip.2014.11.008 25463477 PMC4975561

[CIT0006] Cakir C et al. 2024. Dehydroepiandrosterone modulates the PTEN/PI3K/AKT signaling pathway to alleviate 4-vinylcyclohexene diepoxide-induced premature ovarian insufficiency in rats. Exp Anim. 73(3):319–335. 10.1538/expanim.23-0179 38494723 PMC11254495

[CIT0007] Cao LB et al. 2020. Systemic changes in a mouse model of VCD-induced premature ovarian failure. Life Sci. 262:118543. 10.1016/j.lfs.2020.118543 33038381

[CIT0008] Cao XJ et al. 2018. A randomized, double-blind, placebo-controlled trial of Chinese herbal medicine capsules for the treatment of premature ovarian insufficiency. Menopause. 25(8):918–926. 10.1097/gme.0000000000001099 29533368

[CIT0009] Chen H et al. 2024. Danggui Shaoyao San protects cyclophosphamide-induced premature ovarian failure by inhibiting apoptosis and oxidative stress through the regulation of the SIRT1/p53 signaling pathway. J Ethnopharmacol. 323:117718. 10.1016/j.jep.2024.117718 38181933

[CIT0010] Chen M et al. 2022. Electro-acupuncture regulates metabolic disorders of the liver and kidney in premature ovarian failure mice. Front Endocrinol (Lausanne). 13:882214. 10.3389/fendo.2022.882214 35957829 PMC9359440

[CIT0011] Chen S et al. 2024. A metabolome-wide Mendelian randomization study prioritizes causal circulating metabolites for reproductive disorders including primary ovarian insufficiency, polycystic ovary syndrome, and abnormal spermatozoa. J Ovarian Res. 17(1):166. 10.1186/s13048-024-01486-1 39143642 PMC11325614

[CIT0012] Chen Y, Chen J, Wu J, Qu X, Zhang Z. 2025. Metabolome-wide Mendelian randomization assessing the causal relationship between blood metabolites and primary ovarian insufficiency. Clin Nutr ESPEN. 65:331–338. 10.1016/j.clnesp.2024.11.013 39615787

[CIT0013] Cho J et al. 2020. L-Arginine prevents cereblon-mediated ubiquitination of glucokinase and stimulates glucose-6-phosphate production in pancreatic β-cells. Commun Biol. 3(1):497. 10.1038/s42003-020-01226-3 32901087 PMC7479149

[CIT0014] Dang L et al. 2024. Zishen Yutai pills restore fertility in premature ovarian failure through regulating arachidonic acid metabolism and the ATK pathway. J Ethnopharmacol. 324:117782. 10.1016/j.jep.2024.117782 38272104

[CIT0015] Gelberman RH, Posch JL, Jurist JM. 1975. High-pressure injection injuries of the hand. J Bone Joint Surg Am. 57(7):935–937. 10.2106/00004623-197557070-00010 1184643

[CIT0016] Gong L et al. 2024. Kuntai capsule attenuates premature ovarian insufficiency by activating the FOXO3/SIRT5 signaling pathway in mice: a comprehensive study using UHPLC-LTQ-Orbitrap and integrated pharmacology. J Ethnopharmacol. 322:117625. 10.1016/j.jep.2023.117625 38145859

[CIT0017] Greenfield JR et al. 2009. Oral glutamine increases circulating glucagon-like peptide 1, glucagon, and insulin concentrations in lean, obese, and type 2 diabetic subjects. Am J Clin Nutr. 89(1):106–113. 10.3945/ajcn.2008.26362 19056578 PMC4340573

[CIT0018] Hagen-Lillevik S et al. 2021. Pathophysiology and management of classic galactosemic primary ovarian insufficiency. Reprod Fertil. 2(3):R67–r84. 10.1530/raf-21-0014 35118398 PMC8788619

[CIT0019] Hu YY et al. 2023. Jinfeng pills ameliorate premature ovarian insufficiency induced by cyclophosphamide in rats and correlate to modulating IL-17A/IL-6 axis and MEK/ERK signals. J Ethnopharmacol. 307:116242. 10.1016/j.jep.2023.116242 36775079

[CIT0020] Huang Y, Zhang Q, Shen D, Bao X. 2024. Mechanisms of He Shi Yu Lin formula in treating premature ovarian insufficiency: insights from network pharmacology and animal experiments. J Ovarian Res. 17(1):254. 10.1186/s13048-024-01575-1 39731132 PMC11674109

[CIT0021] Ishizuka B. 2021. Current understanding of the etiology, symptomatology, and treatment options in premature ovarian insufficiency (POI). Front Endocrinol (Lausanne). 12:626924. 10.3389/fendo.2021.626924 33716979 PMC7949002

[CIT0022] Jia K et al. 2024. Exploring the mechanism of Si-Ni-San against depression by UPLC-Q-TOF-MS/MS integrated with network pharmacology: experimental research. Ann Med Surg (Lond). 86(1):172–189. 10.1097/ms9.0000000000001464 38222693 PMC10783272

[CIT0023] Jiang L et al. 2021. Hormone replacement therapy reverses gut microbiome and serum metabolome alterations in premature ovarian insufficiency. Front Endocrinol (Lausanne). 12:794496. 10.3389/fendo.2021.794496 35002971 PMC8733385

[CIT0024] Jiang X-L et al. 2022. Jian-Pi-Yi-Shen decoction inhibits mitochondria-dependent granulosa cell apoptosis in a rat model of POF. Aging (Albany NY). 14(20):8321–8345. 10.18632/aging.204320 36309912 PMC9648799

[CIT0025] John GB, Gallardo TD, Shirley LJ, Castrillon DH. 2008. Foxo3 is a PI3K-dependent molecular switch controlling the initiation of oocyte growth. Dev Biol. 321(1):197–204. 10.1016/j.ydbio.2008.06.017 18601916 PMC2548299

[CIT0026] Kappeler CJ, Hoyer PB. 2012. 4-vinylcyclohexene diepoxide: a model chemical for ovotoxicity. Syst Biol Reprod Med. 58(1):57–62. 10.3109/19396368.2011.648820 22239082 PMC3307534

[CIT0027] Ke H et al. 2023. Landscape of pathogenic mutations in premature ovarian insufficiency. Nat Med. 29(2):483–492. 10.1038/s41591-022-02194-3 36732629 PMC9941050

[CIT0028] Kinote A et al. 2012. Fructose-induced hypothalamic AMPK activation stimulates hepatic PEPCK and gluconeogenesis due to increased corticosterone levels. Endocrinology. 153(8):3633–3645. 10.1210/en.2012-1341 22585831

[CIT0029] Knauff EA et al. 2008. Lipid profile of women with premature ovarian failure. Menopause. 15(5):919–923. 10.1097/gme.0b013e31816b4509 18551082

[CIT0030] Kunicki M, Rudnicka E, Skórska J, Calik-Ksepka AI, Smolarczyk R. 2018. Insulin resistance indexes in women with premature ovarian insufficiency - a pilot study. Ginekol Pol. 89(7):364–369. 10.5603/GP.a2018.0062 30091445

[CIT0031] Kur P et al. 2021. The postnatal offspring of finasteride-treated male rats shows hyperglycaemia, elevated hepatic glycogen storage and altered GLUT2, IR, and AR expression in the liver. Int J Mol Sci. 22(3):1242. 10.3390/ijms22031242 33513940 PMC7865973

[CIT0032] Kurihara T. 1985. Response characteristics of slow-adapting units innervating the molar buccal gingiva and alveolar mucosa of the cat mandible to repetitive mechanical stimulation. Shikwa Gakuho. 85(7):979–996. 3867147

[CIT0033] Lan Y, Jin B, Fan Y, Huang Y, Zhou J. 2026. The circadian rhythm regulates the hepato-ovarian axis linking polycystic ovary syndrome and non-alcoholic fatty liver disease. Biochem Genet. 64(1):262–285. 10.1007/s10528-024-11010-1 39826031

[CIT0034] Li M et al. 2023. The global prevalence of premature ovarian insufficiency: a systematic review and meta-analysis. Climacteric. 26(2):95–102. 10.1080/13697137.2022.2153033 36519275

[CIT0035] Ling L et al. 2019. Human amnion-derived mesenchymal stem cell (hAD-MSC) transplantation improves ovarian function in rats with premature ovarian insufficiency (POI) at least partly through a paracrine mechanism. Stem Cell Res Ther. 10(1):46. 10.1186/s13287-019-1136-x 30683144 PMC6347748

[CIT0036] Liu D et al. 2023. The hepato-ovarian axis: genetic evidence for a causal association between non-alcoholic fatty liver disease and polycystic ovary syndrome. BMC Med. 21(1):62. 10.1186/s12916-023-02775-0 36800955 PMC9940436

[CIT0037] Liu X et al. 2023. Network and experimental pharmacology on mechanism of Si-Wu-tang improving ovarian function in a mouse model of premature ovarian failure induced by cyclophosphamide. J Ethnopharmacol. 301:115842. 10.1016/j.jep.2022.115842 36265674

[CIT0038] Luo L-L et al. 2019. L-glutamine protects mouse brain from ischemic injury via up-regulating heat shock protein 70. CNS Neurosci Ther. 25(9):1030–1041. 10.1111/cns.13184 31218845 PMC6698979

[CIT0039] Meneyrol K et al. 2021. Ovarian insufficiency impairs glucose-stimulated insulin secretion through activation of hypothalamic de novo ceramide synthesis. Metabolism. 123:154846. 10.1016/j.metabol.2021.154846 34371064

[CIT0040] Miao M et al. 2020. Tandem mass tag-based proteomic analysis reveals the treatment mechanism of Bushen Huoxue Formula on psychological stress-induced premature ovarian insufficiency. J Ethnopharmacol. 258:112870. 10.1016/j.jep.2020.112870 32311483

[CIT0041] Miotto PM et al. 2024. Liver-derived extracellular vesicles improve whole-body glycaemic control via inter-organ communication. Nat Metab. 6(2):254–272. 10.1038/s42255-023-00971-z 38263317

[CIT8015327] Myers M, Britt KL, Wreford NGM, Ebling FJP, Kerr JB. 2004. Methods for quantifying follicular numbers within the mouse ovary. Reproduction. 127(5):569–580. 10.1530/rep.1.00095. 15129012

[CIT0042] Nash Z, Davies M. 2024. Premature ovarian insufficiency. BMJ. 384:e077469. 10.1136/bmj-2023-077469 38508679

[CIT0043] Peng H et al. 2019. Zuogui Pills inhibit mitochondria-dependent apoptosis of follicles in a rat model of premature ovarian failure. J Ethnopharmacol. 238:111855. 10.1016/j.jep.2019.111855 30953821

[CIT0044] Peng Y et al. 2025. Efficacy and safety of HYYK formula for residual follicle revival in premature ovarian insufficiency: a multicenter, randomized, double-blind, placebo-controlled trial protocol. BMC Complement Med Ther. 25(1):46. 10.1186/s12906-025-04803-3 39934764 PMC11817039

[CIT0045] Podfigurna A, Stellmach A, Szeliga A, Czyzyk A, Meczekalski B. 2018. Metabolic profile of patients with premature ovarian insufficiency. J Clin Med. 7(10):374. 10.3390/jcm7100374 30347864 PMC6210159

[CIT0046] Qiao S et al. 2023. Intra-pituitary follicle-stimulating hormone signaling regulates hepatic lipid metabolism in mice. Nat Commun. 14(1):1098. 10.1038/s41467-023-36681-z 36841874 PMC9968338

[CIT471054] Qin X et al. 2022. TrkB agonist antibody ameliorates fertility deficits in aged and cyclophosphamide-induced premature ovarian failure model mice. Nat Commun. 13(1):914 10.1038/s41467-022-28611-2. 35177657 PMC8854395

[CIT0047] Reddy P, Zheng W, Liu K. 2010. Mechanisms maintaining the dormancy and survival of mammalian primordial follicles. Trends Endocrinol Metab. 21(2):96–103. 10.1016/j.tem.2009.10.001 19913438

[CIT0048] Shimizu K et al. 2010. Anti-obesity effect of phosphatidylinositol on diet-induced obesity in mice. J Agric Food Chem. 58(21):11218–11225. 10.1021/jf102075j 20931972

[CIT59003191] Shin E-Y et al. 2021. Prevention of chemotherapy-induced premature ovarian insufficiency in mice by scaffold-based local delivery of human embryonic stem cell-derived mesenchymal progenitor cells. Stem Cell Res Ther. 12(1):431 10.1186/s13287-021-02479-3. 34332643 PMC8325282

[CIT0049] Su C et al. 2023. Dingkun Pill modulate ovarian function in chemotherapy-induced premature ovarian insufficiency mice by regulating PTEN/PI3K/AKT/FOXO3a signaling pathway. J Ethnopharmacol. 315:116703. 10.1016/j.jep.2023.116703 37257704

[CIT0050] Su X et al. 2021. Effect of Jiajian Guishen Formula on the senescence-associated heterochromatic foci in mouse ovaria after induction of premature ovarian aging by the endocrine-disrupting agent 4-vinylcyclohexene diepoxide. J Ethnopharmacol. 269:113720. 10.1016/j.jep.2020.113720 33358858

[CIT0051] Sullivan LB et al. 2013. The proto-oncometabolite fumarate binds glutathione to amplify ROS-dependent signaling. Mol Cell. 51(2):236–248. 10.1016/j.molcel.2013.05.003 23747014 PMC3775267

[CIT7024779] Sun M et al. 2013. Adipose-derived stem cells improved mouse ovary function after chemotherapy-induced ovary failure. Stem Cell Res Ther. 4(4):80 10.1186/scrt231. 23838374 PMC3854877

[CIT0052] Targher G, Rossini M, Lonardo A. 2016. Evidence that non-alcoholic fatty liver disease and polycystic ovary syndrome are associated by necessity rather than chance: a novel hepato-ovarian axis? Endocrine. 51(2):211–221. 10.1007/s12020-015-0640-8 26024975

[CIT0053] Tsutsumi T et al. 2023. The inter-organ crosstalk reveals an inevitable link between MAFLD and extrahepatic diseases. Nutrients. 15(5):1123. 10.3390/nu15051123 36904122 PMC10005526

[CIT0054] Turnbull J et al. 2013. Deficiency of a glycogen synthase-associated protein, Epm2aip1, causes decreased glycogen synthesis and hepatic insulin resistance. J Biol Chem. 288(48):34627–34637. 10.1074/jbc.M113.483198 24142699 PMC3843075

[CIT0055] Wang C et al. 2014. Hepatic overexpression of ATP synthase β subunit activates PI3K/Akt pathway to ameliorate hyperglycemia of diabetic mice. Diabetes. 63(3):947–959. 10.2337/db13-1096 24296716

[CIT0056] Wang L, Liu J, Nie G, Li Y, Yang H. 2021. Danggui Buxue tang rescues Folliculogenesis and ovarian cell apoptosis in rats with premature ovarian insufficiency. Evid Based Complement Alternat Med. 2021:6614302. 10.1155/2021/6614302 34035823 PMC8118728

[CIT9980686] Wu G, Hu X, Ding J, Yang J. 2020. The effect of glutamine on Dehydroepiandrosterone-induced polycystic ovary syndrome rats. J Ovarian Res. 13(1):57 10.1186/s13048-020-00650-7. 32386521 PMC7211337

[CIT0057] Wang W et al. 2024. The microbial communities and metabolic profiles of follicular fluid in patients with premature ovarian insufficiency. Front Endocrinol (Lausanne). 15:1447397. 10.3389/fendo.2024.1447397 39839476 PMC11746125

[CIT0058] Xiu Z et al. 2023. Zigui-Yichong-Fang protects against cyclophosphamide-induced premature ovarian insufficiency via the SIRT1/Foxo3a pathway. J Ethnopharmacol. 314:116608. 10.1016/j.jep.2023.116608 37150421

[CIT0059] Xu X et al. 2024. Bu-Shen-Ning-Xin decoction ameliorates premature ovarian insufficiency by suppressing oxidative stress through rno_circRNA_012284/rno_miR-760-3p/HBEGF pathway. Phytomedicine. 133:155920. 10.1016/j.phymed.2024.155920 39126922

[CIT0060] Yan J et al. 2024. Sleep deprivation causes gut dysbiosis impacting on systemic metabolomics leading to premature ovarian insufficiency in adolescent mice. Theranostics. 14(9):3760–3776. 10.7150/thno.95197 38948060 PMC11209713

[CIT0061] Yu Q, Zhu Y, Zuo W. 2024. Effect of Wubie Fanchun oral liquid on ovarian unction in patients with premature ovarian insufficiency based on ropensity score matching method. Guiding J Trad Chin Med Pharmacol. 30(07):101–105. 10.13862/j.cn43-1446/r.2024.07.020

[CIT0062] Yu Z et al. 2023. Integrated metabolomics and transcriptomics to reveal biomarkers and mitochondrial metabolic dysregulation of premature ovarian insufficiency. Front Endocrinol (Lausanne). 14:1280248. 10.3389/fendo.2023.1280248 38179298 PMC10764474

[CIT0063] Zhang J et al. 2024. PDCD4 deficiency improved 4-vinylcyclohexene dioxide-induced mouse premature ovarian insufficiency. Reprod Biomed Online. 48(4):103685. 10.1016/j.rbmo.2023.103685 38324980

[CIT0064] Zheng S, Ma M, Chen Y, Li M. 2022. Effects of quercetin on ovarian function and regulation of the ovarian PI3K/Akt/FoxO3a signalling pathway and oxidative stress in a rat model of cyclophosphamide-induced premature ovarian failure. Basic Clin Pharmacol Toxicol. 130(2):240–253. 10.1111/bcpt.13696 34841658

[CIT0065] Zhou F et al. 2021. Si-Wu-Tang facilitates ovarian function through improving ovarian microenvironment and angiogenesis in a mouse model of premature ovarian failure. J Ethnopharmacol. 280:114431. 10.1016/j.jep.2021.114431 34293457

[CIT0066] Zhou X-Y et al. 2023. Plasma metabolomic characterization of premature ovarian insufficiency. J Ovarian Res. 16(1):2. 10.1186/s13048-022-01085-y 36600288 PMC9814329

