# Supplementary material for: Wubie Fanchun Formula-inducible metabolites in primary ovarian insufficiency model mice that facilitate ovarian renovation
Source: Pharm Biol. 2026 May 12;64(1):725–48. doi: 10.1080/13880209.2026.2668132 (PMC13169455; doi:10.1080/13880209.2026.2668132)

Supplemental Figure 1

key metabolites conformation

phosphoinositol----Mice samples

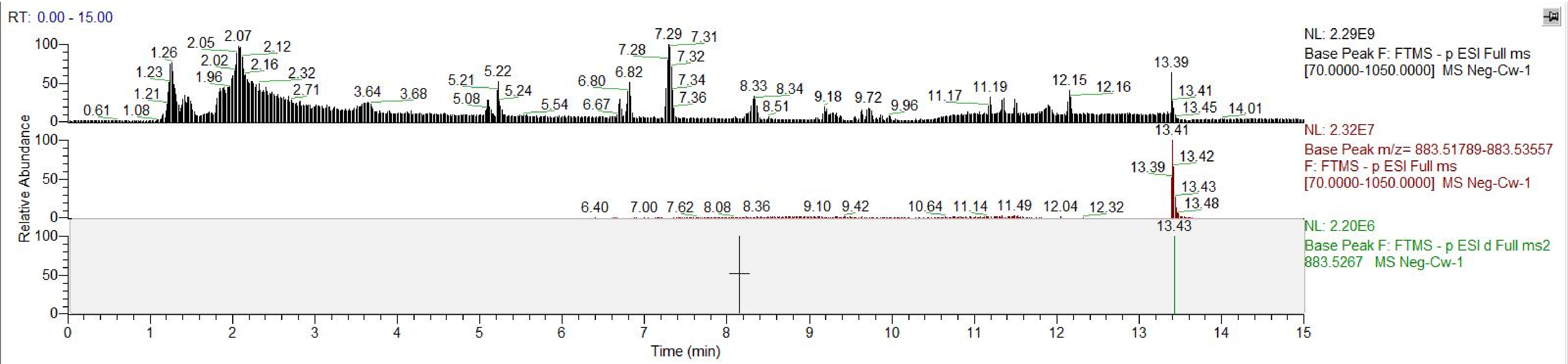

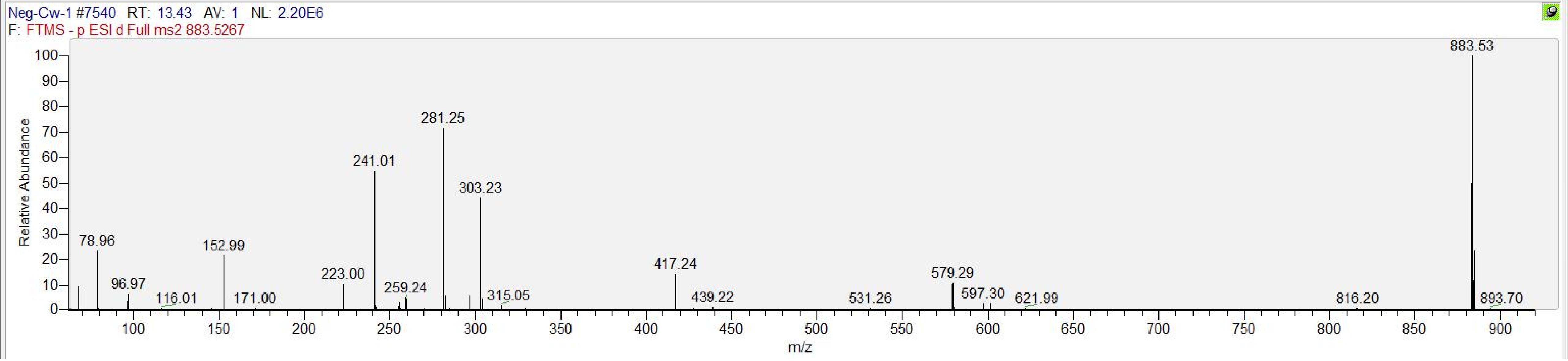

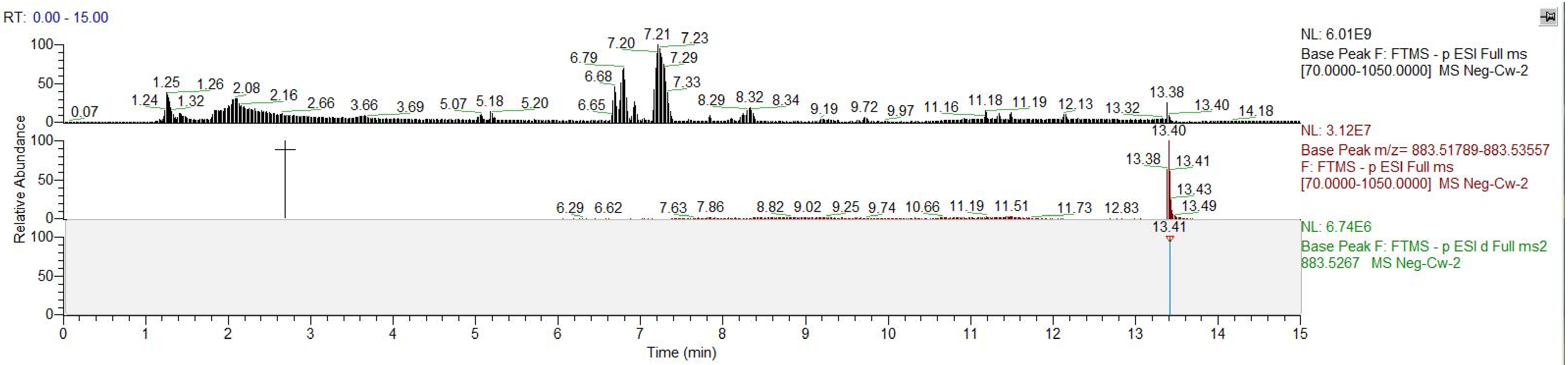
RT: 0.00 - 15.00
NL: 6.01E9
Base Peak F: FTMS - p ESI Full ms
[70.0000-1050.0000] MS Neg-Cw-2
NL: 3.12E7
Base Peak m/z= 883.51789-883.53557
F: FTMS - p ESI Full ms
[70.0000-1050.0000] MS Neg-Cw-2
NL: 6.74E6
Base Peak F: FTMS - p ESI d Full ms2
883.5267 MS Neg-Cw-2
Relative Abundance
Time (min)

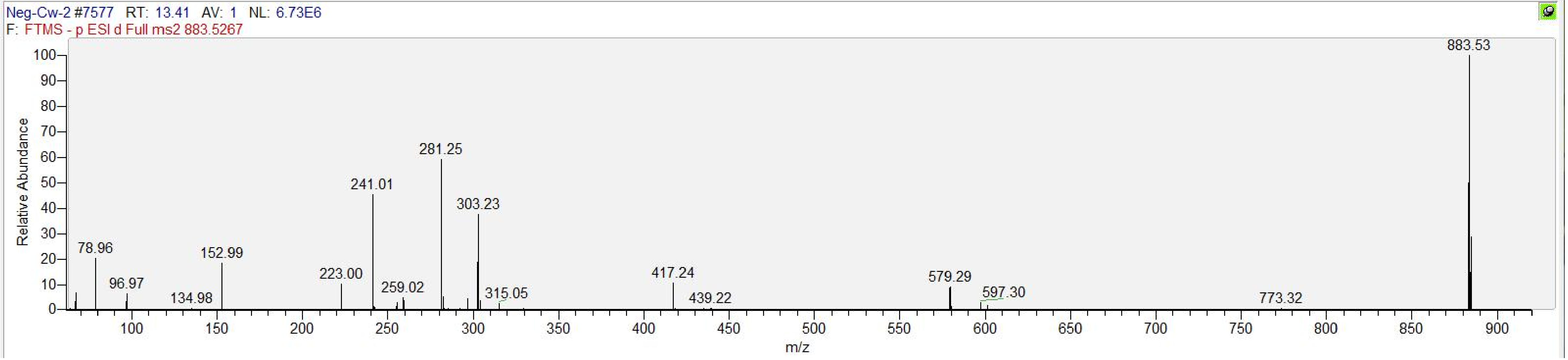
Neg-Cw-2 #7577 RT: 13.41 AV: 1 NL: 6.73E6
F: FTMS - p ESI d Full ms2 883.5267
883.53
281.25
241.01
303.23
78.96
152.99
223.00
96.97
259.02
134.98
315.05
417.24
439.22
579.29
597.30
773.32
Relative Abundance
m/z

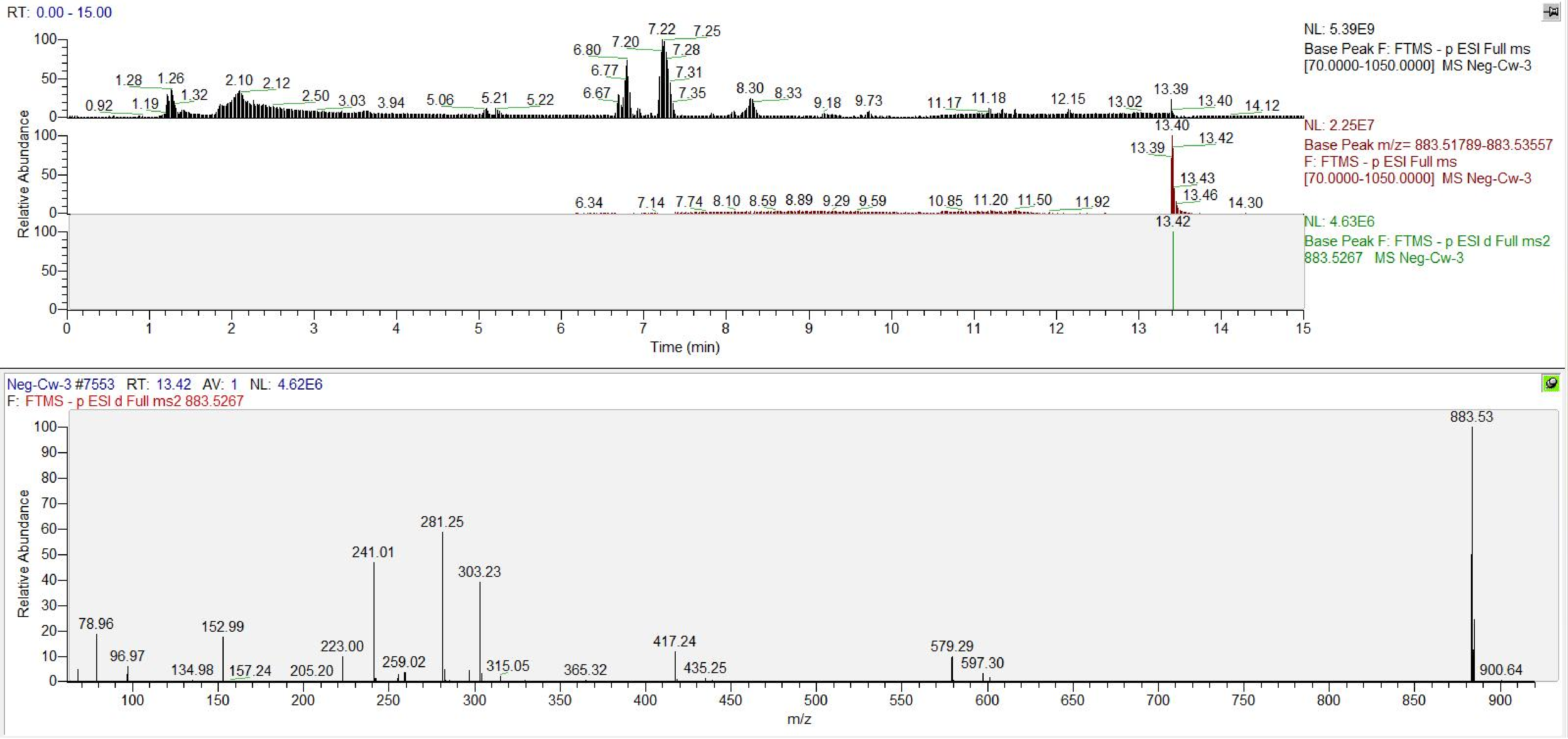

RT: 0.00 - 15.00
NL: 5.39E9
Base Peak F: FTMS - p ESI Full ms [70.0000-1050.0000] MS Neg-Cw-3
NL: 2.25E7
Base Peak m/z= 883.51789-883.53557 F: FTMS - p ESI Full ms [70.0000-1050.0000] MS Neg-Cw-3
NL: 4.63E6
Base Peak F: FTMS - p ESI d Full ms2 883.5267 MS Neg-Cw-3
Relative Abundance
Time (min)
Neg-Cw-3 #7553 RT: 13.42 AV: 1 NL: 4.62E6
F: FTMS - p ESI d Full ms2 883.5267
883.53
281.25
241.01
303.23
78.96
152.99
223.00
96.97
134.98
157.24
205.20
259.02
315.05
365.32
417.24
435.25
579.29
597.30
900.64
m/z

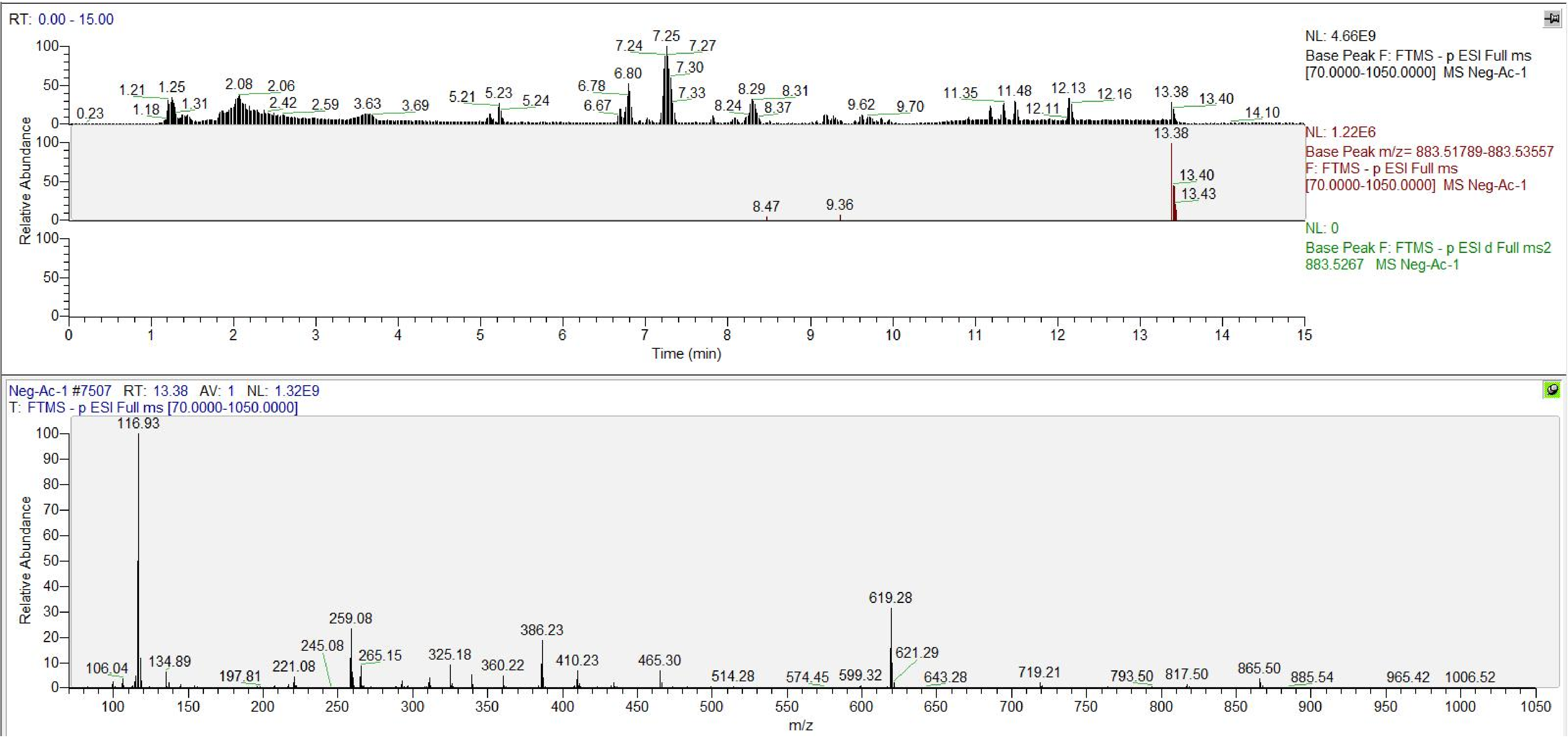
RT: 0.00 - 15.00
NL: 4.66E9
Base Peak F: FTMS - p ESI Full ms
[70.0000-1050.0000] MS Neg-Ac-1
NL: 1.22E6
Base Peak m/z= 883.51789-883.53557
F: FTMS - p ESI Full ms
[70.0000-1050.0000] MS Neg-Ac-1
NL: 0
Base Peak F: FTMS - p ESI d Full ms2
883.5267 MS Neg-Ac-1
Relative Abundance
Time (min)
Neg-Ac-1 #7507 RT: 13.38 AV: 1 NL: 1.32E9
T: FTMS - p ESI Full ms [70.0000-1050.0000]
Relative Abundance
m/z

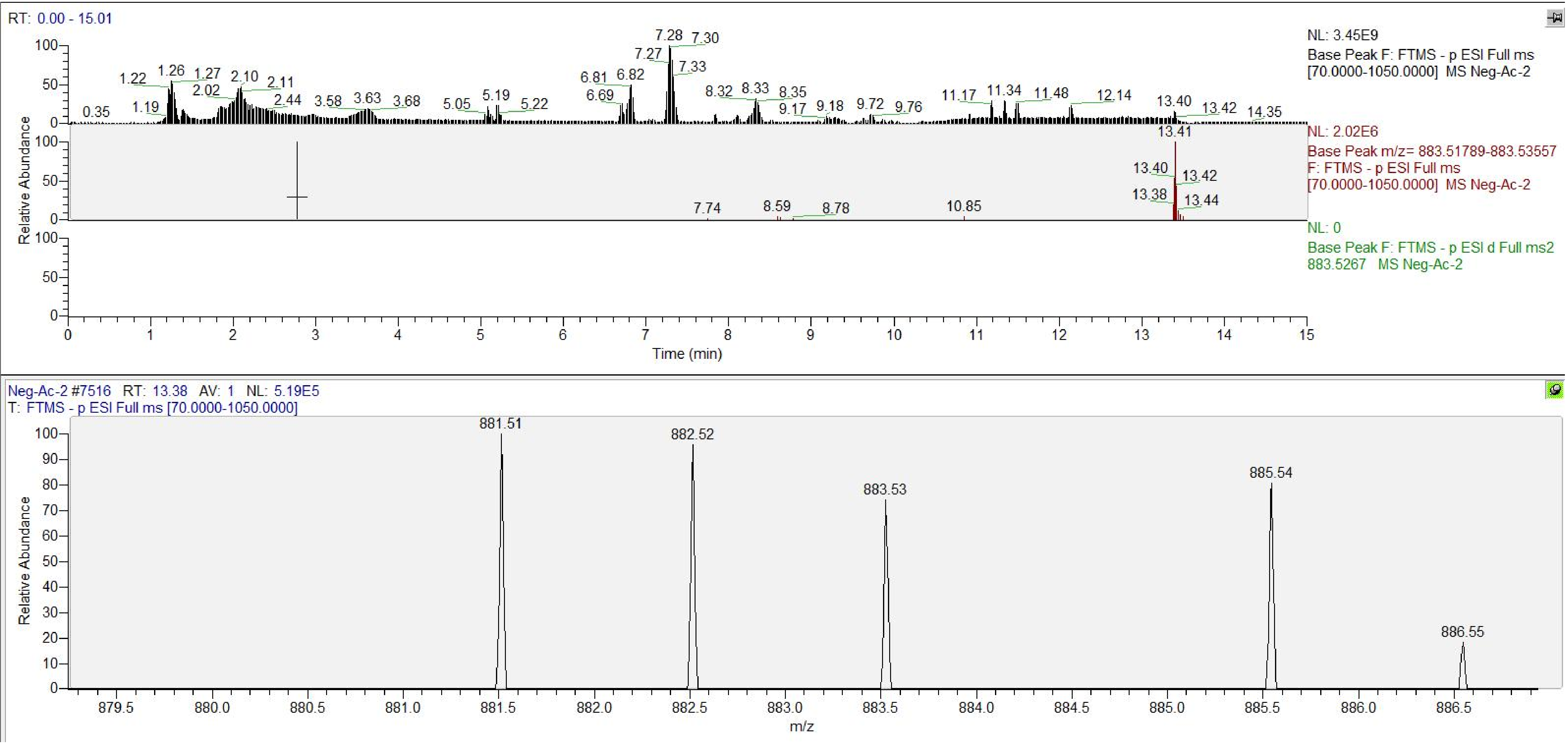
RT: 0.00 - 15.01
NL: 3.45E9
Base Peak F: FTMS - p ESI Full ms [70.0000-1050.0000] MS Neg-Ac-2
NL: 2.02E6
Base Peak m/z= 883.51789-883.53557 F: FTMS - p ESI Full ms [70.0000-1050.0000] MS Neg-Ac-2
NL: 0
Base Peak F: FTMS - p ESI d Full ms2 883.5267 MS Neg-Ac-2
Relative Abundance
Time (min)
Neg-Ac-2 #7516 RT: 13.38 AV: 1 NL: 5.19E5
T: FTMS - p ESI Full ms [70.0000-1050.0000]
881.51
882.52
883.53
885.54
886.55
Relative Abundance
m/z

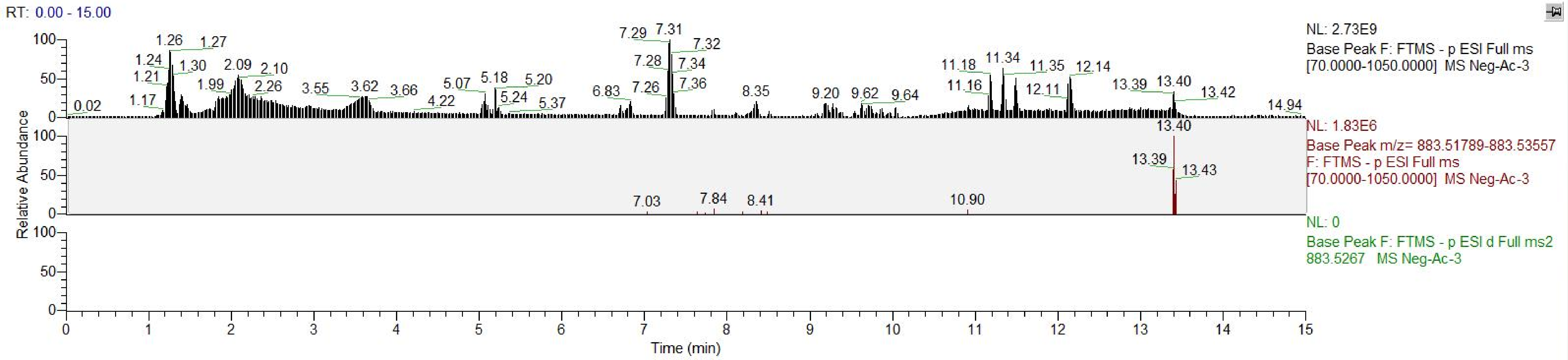
RT: 0.00 - 15.00
NL: 2.73E9
Base Peak F: FTMS - p ESI Full ms [70.0000-1050.0000] MS Neg-Ac-3
NL: 1.83E6
Base Peak m/z= 883.51789-883.53557 F: FTMS - p ESI Full ms [70.0000-1050.0000] MS Neg-Ac-3
NL: 0
Base Peak F: FTMS - p ESI d Full ms2 883.5267 MS Neg-Ac-3
Relative Abundance
Time (min)

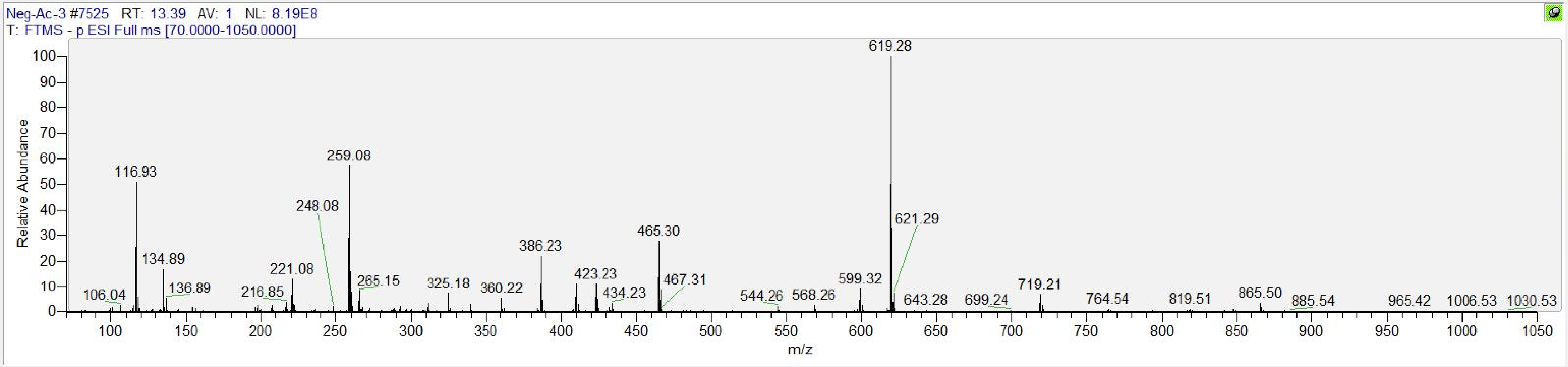
Neg-Ac-3 #7525 RT: 13.39 AV: 1 NL: 8.19E8
T: FTMS - p ESI Full ms [70.0000-1050.0000]
619.28
259.08
116.93
248.08
621.29
465.30
386.23
Relative Abundance
m/z

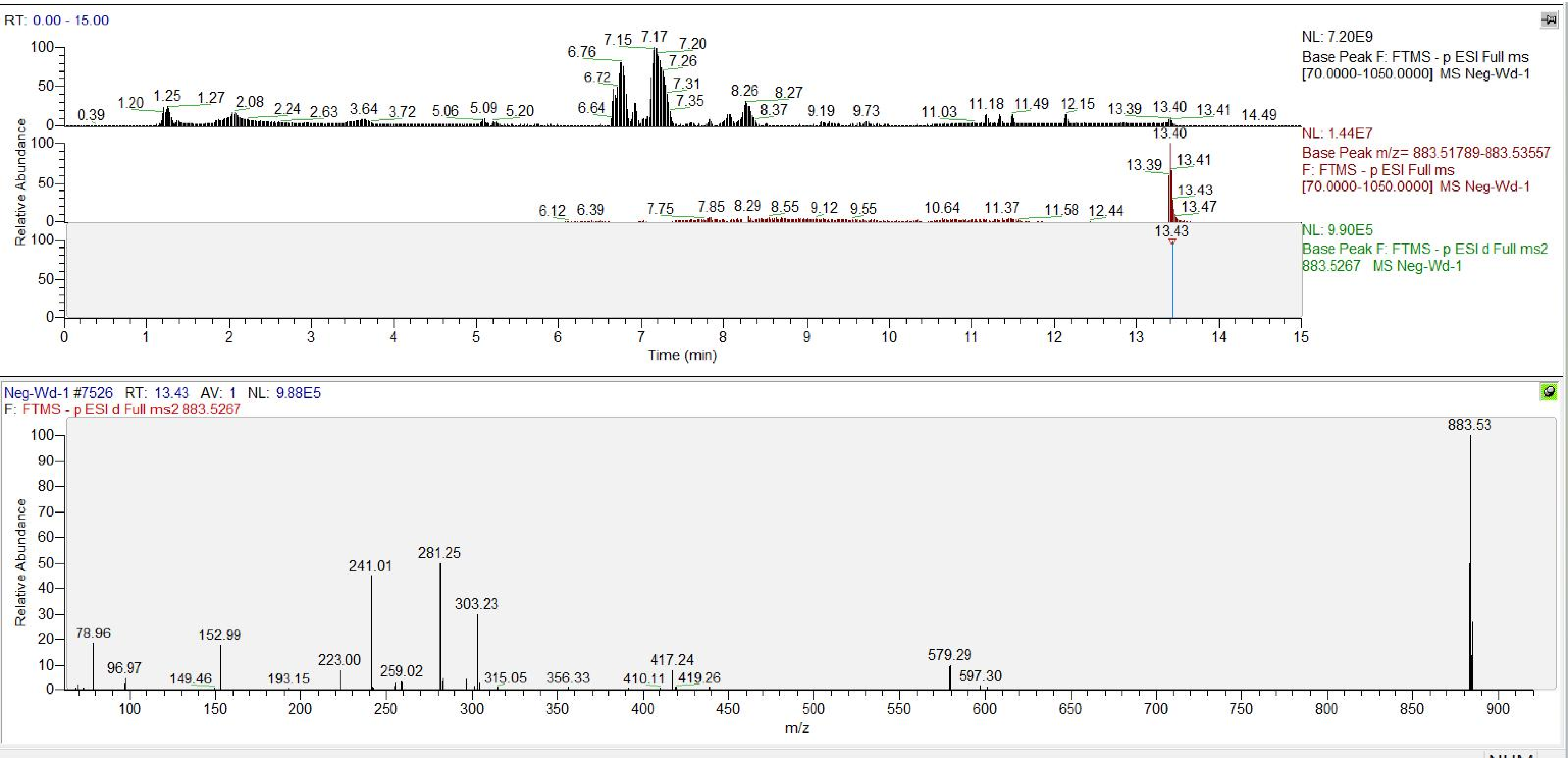

RT: 0.00 - 15.00
NL: 7.20E9
Base Peak F: FTMS - p ESI Full ms [70.0000-1050.0000] MS Neg-Wd-1
NL: 1.44E7
Base Peak m/z= 883.51789-883.53557 F: FTMS - p ESI Full ms [70.0000-1050.0000] MS Neg-Wd-1
NL: 9.90E5
Base Peak F: FTMS - p ESI d Full ms2 883.5267 MS Neg-Wd-1
Relative Abundance
Time (min)
Neg-Wd-1 #7526 RT: 13.43 AV: 1 NL: 9.88E5
F: FTMS - p ESI d Full ms2 883.5267
883.53
281.25
241.01
303.23
78.96
152.99
223.00
579.29
417.24
m/z

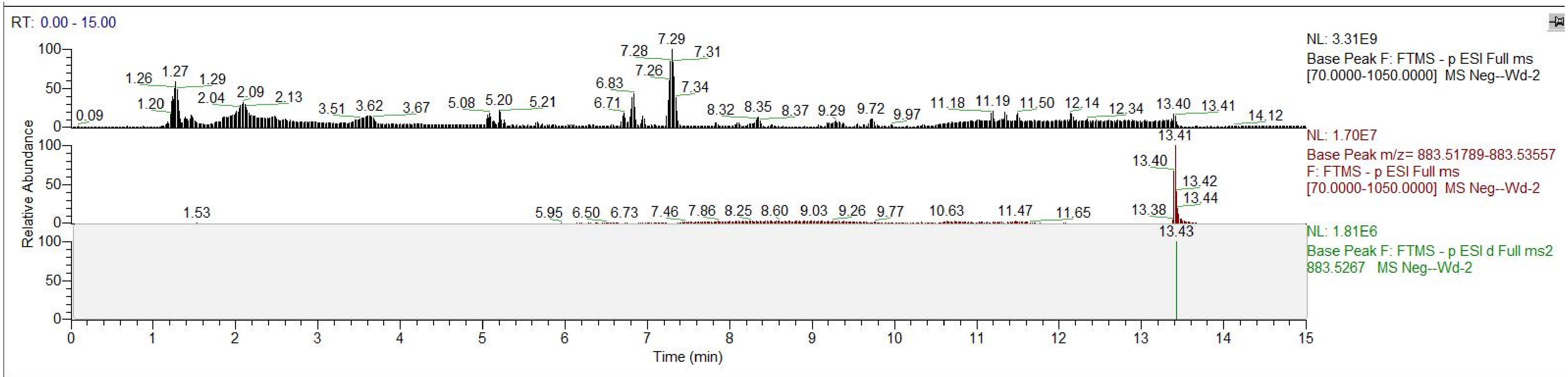

RT: 0.00 - 15.00
NL: 3.31E9
Base Peak F: FTMS - p ESI Full ms [70.0000-1050.0000] MS Neg--Wd-2
NL: 1.70E7
Base Peak m/z= 883.51789-883.53557 F: FTMS - p ESI Full ms [70.0000-1050.0000] MS Neg--Wd-2
NL: 1.81E6
Base Peak F: FTMS - p ESI d Full ms2 883.5267 MS Neg--Wd-2
Relative Abundance
Time (min)

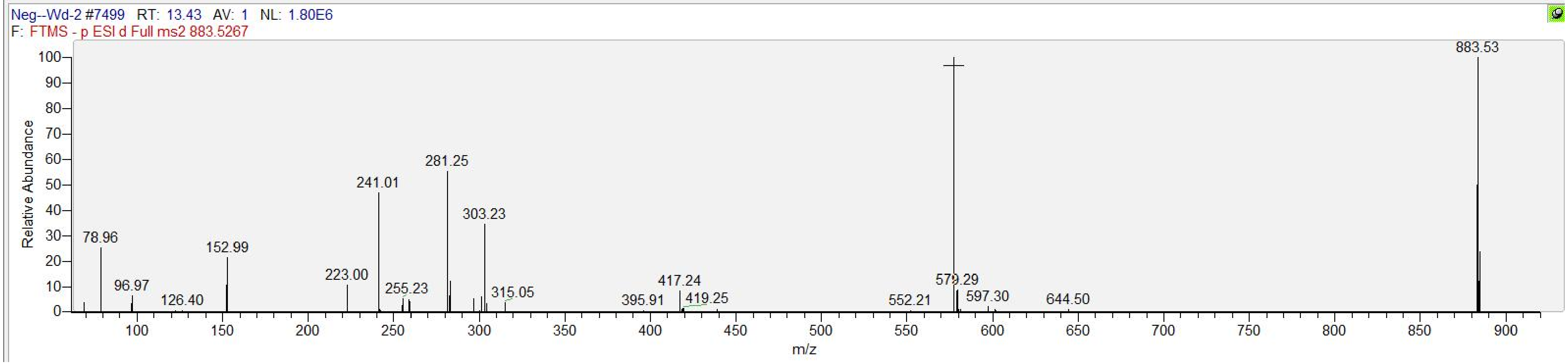

Neg--Wd-2 #7499 RT: 13.43 AV: 1 NL: 1.80E6
F: FTMS - p ESI d Full ms2 883.5267
883.53
281.25
241.01
303.23
78.96
152.99
223.00
255.23
96.97
126.40
315.05
417.24
419.25
395.91
579.29
597.30
552.21
644.50
Relative Abundance
m/z

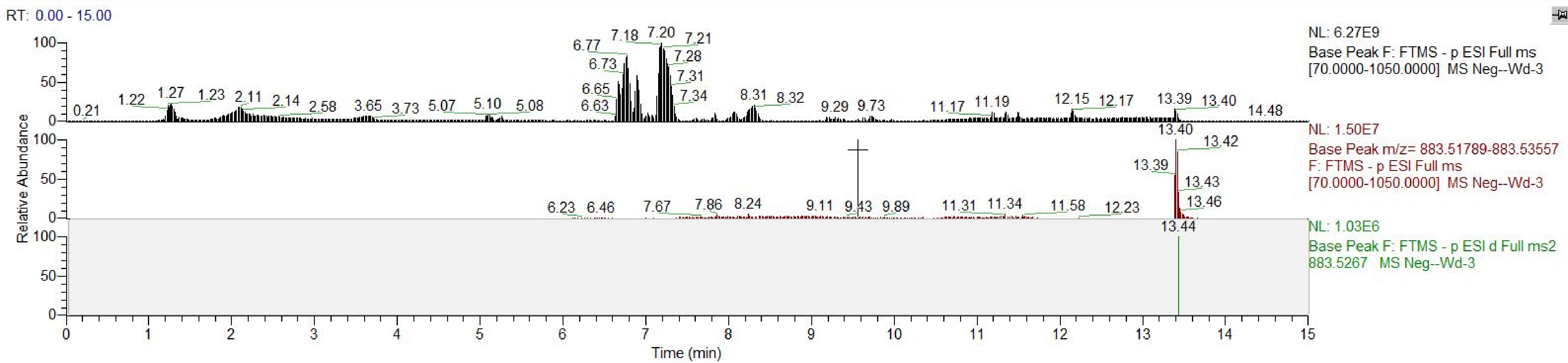

RT: 0.00 - 15.00
NL: 6.27E9
Base Peak F: FTMS - p ESI Full ms [70.0000-1050.0000] MS Neg--Wd-3
NL: 1.50E7
Base Peak m/z= 883.51789-883.53557 F: FTMS - p ESI Full ms [70.0000-1050.0000] MS Neg--Wd-3
NL: 1.03E6
Base Peak F: FTMS - p ESI d Full ms2 883.5267 MS Neg--Wd-3
Relative Abundance
Time (min)

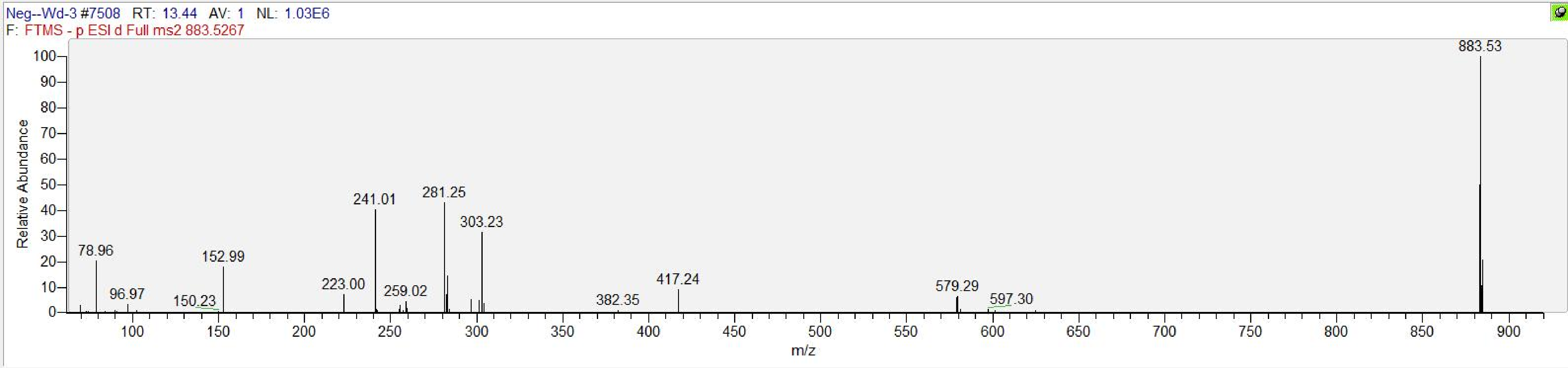

Neg--Wd-3 #7508 RT: 13.44 AV: 1 NL: 1.03E6
F: FTMS - p ESI d Full ms2 883.5267
883.53
241.01
281.25
303.23
78.96
152.99
96.97
150.23
223.00
259.02
382.35
417.24
579.29
597.30
Relative Abundance
m/z

phosphoinositol---- standard substance

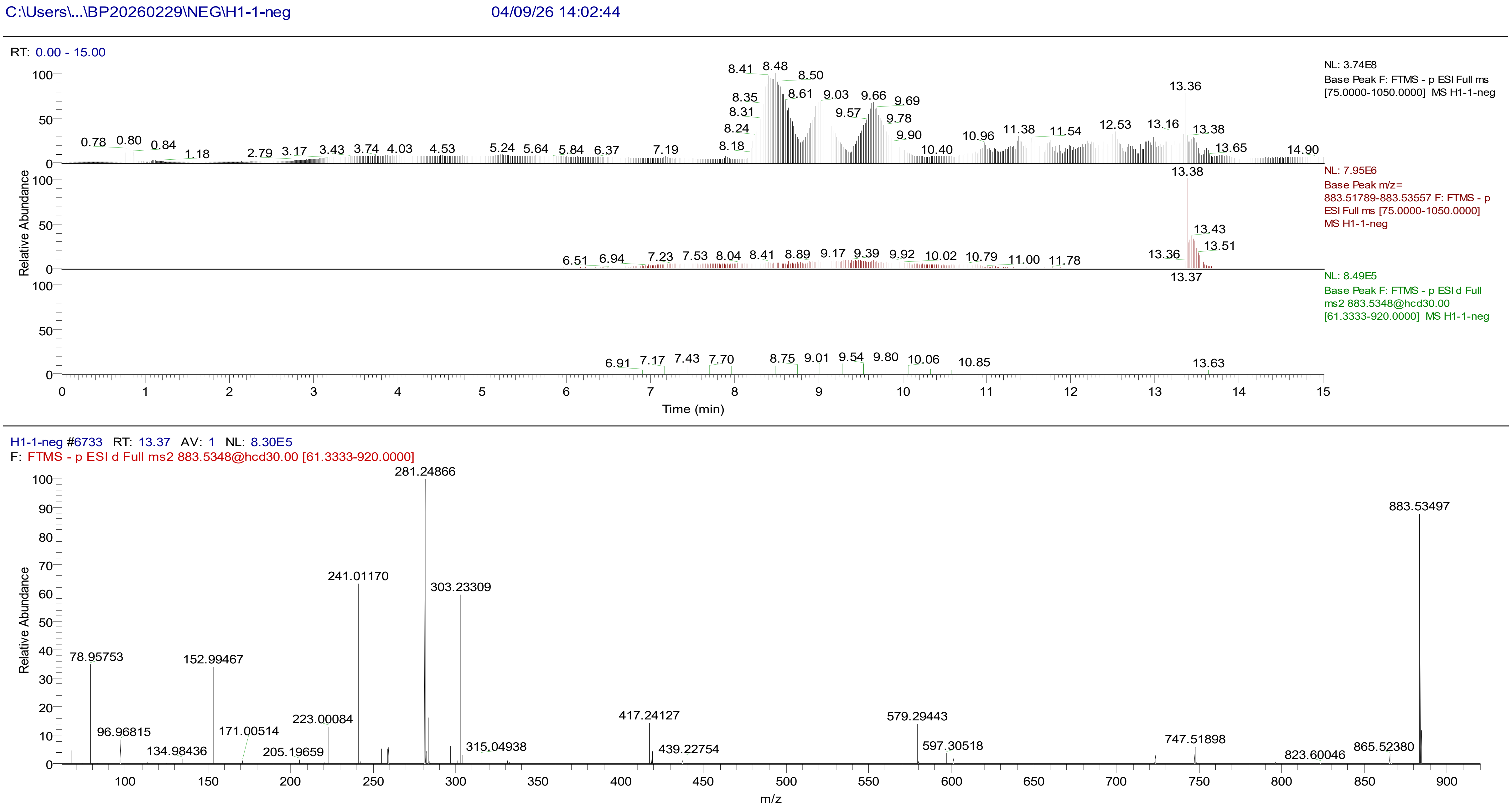

Glutamine----Mice samples:

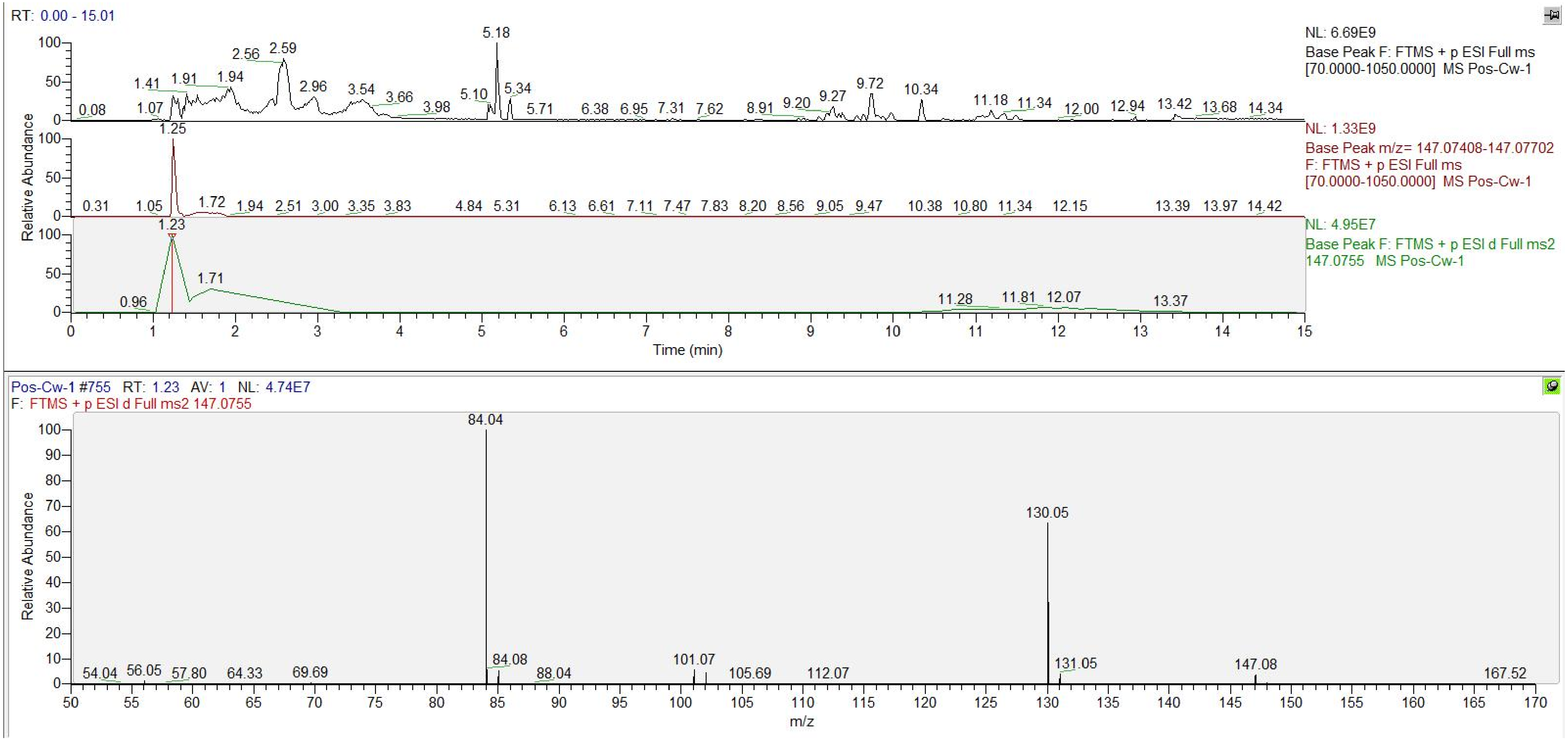

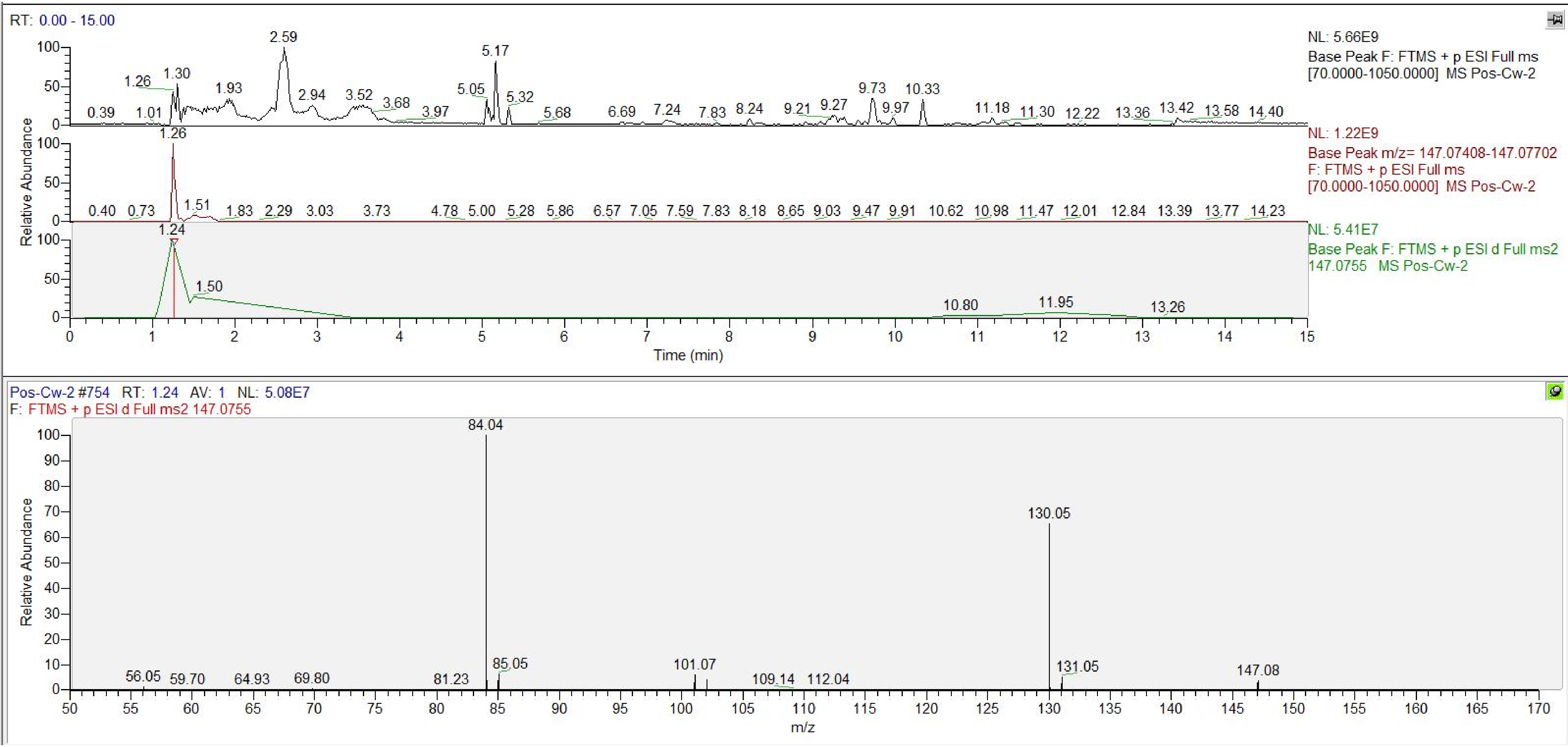

RT: 0.00 - 15.00
NL: 5.66E9
Base Peak F: FTMS + p ESI Full ms [70.0000-1050.0000] MS Pos-Cw-2
NL: 1.22E9
Base Peak m/z= 147.07408-147.07702 F: FTMS + p ESI Full ms [70.0000-1050.0000] MS Pos-Cw-2
NL: 5.41E7
Base Peak F: FTMS + p ESI d Full ms2 147.0755 MS Pos-Cw-2
Relative Abundance
Time (min)
Pos-Cw-2 #754 RT: 1.24 AV: 1 NL: 5.08E7
F: FTMS + p ESI d Full ms2 147.0755
84.04
130.05
85.05
101.07
131.05
147.08
56.05
59.70
64.93
69.80
81.23
109.14
112.04
m/z

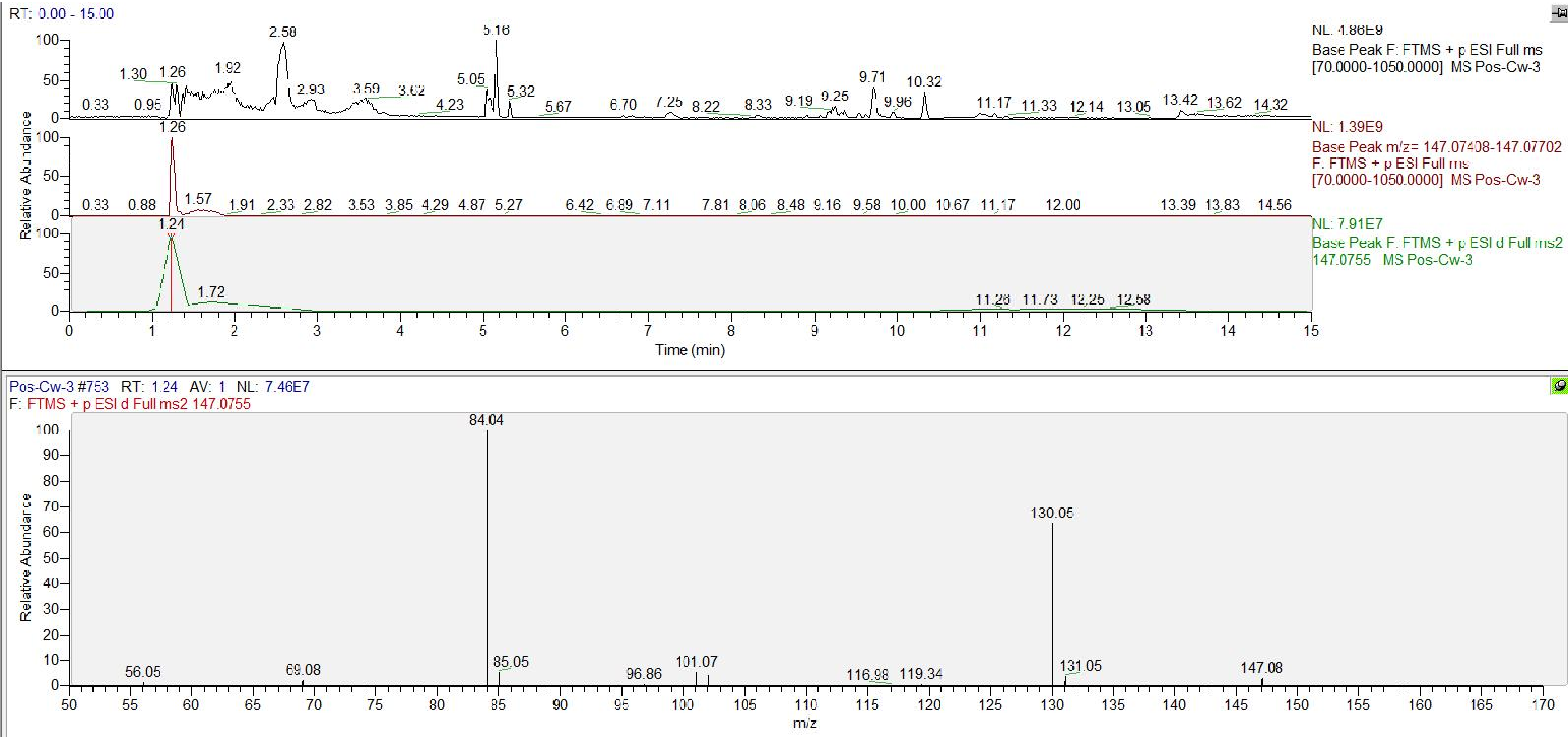

RT: 0.00 - 15.00
NL: 4.86E9
Base Peak F: FTMS + p ESI Full ms [70.0000-1050.0000] MS Pos-Cw-3
NL: 1.39E9
Base Peak m/z= 147.07408-147.07702 F: FTMS + p ESI Full ms [70.0000-1050.0000] MS Pos-Cw-3
NL: 7.91E7
Base Peak F: FTMS + p ESI d Full ms2 147.0755 MS Pos-Cw-3
Relative Abundance
Time (min)
Pos-Cw-3 #753 RT: 1.24 AV: 1 NL: 7.46E7
F: FTMS + p ESI d Full ms2 147.0755
84.04
130.05
56.05
69.08
85.05
96.86
101.07
116.98
119.34
131.05
147.08
Relative Abundance
m/z

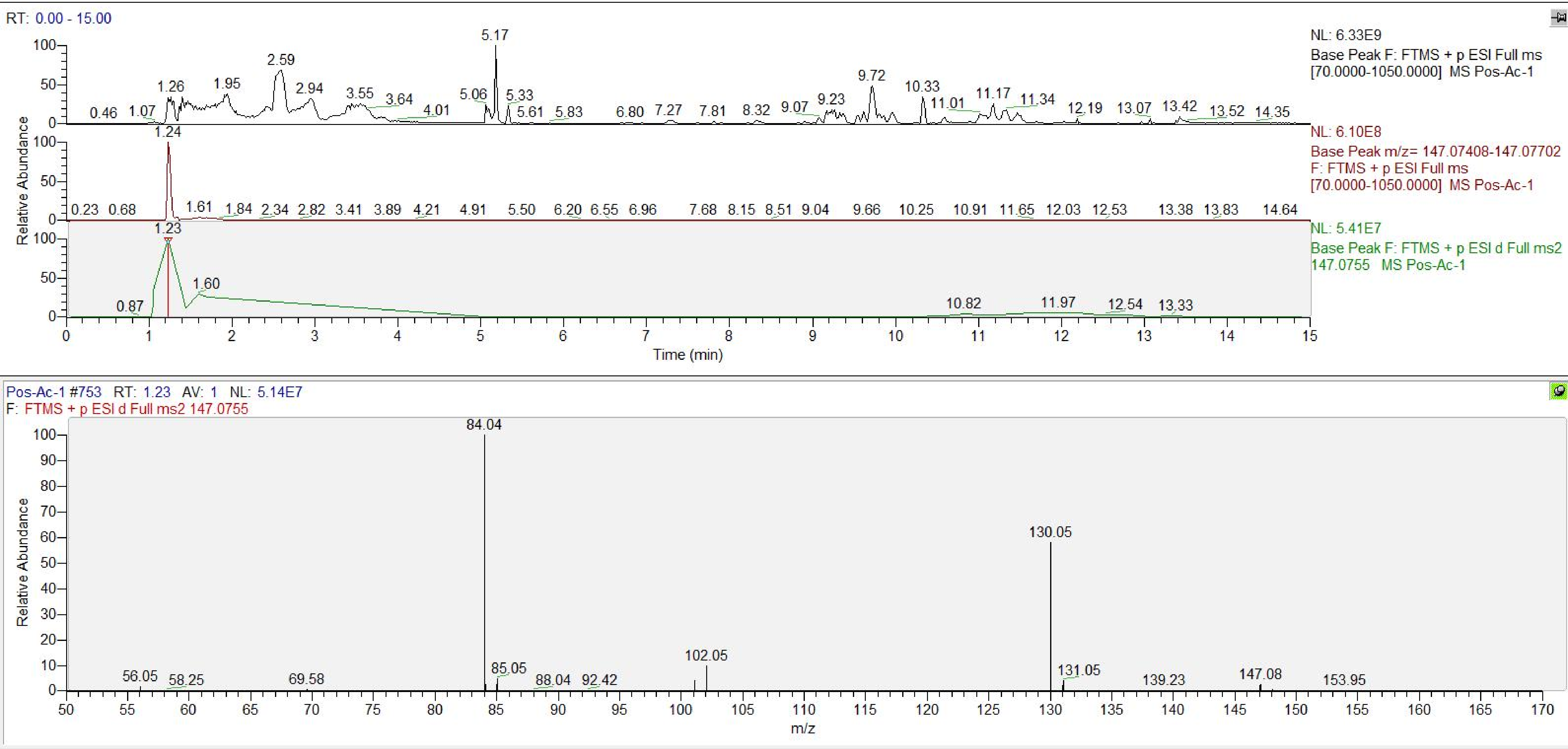

RT: 0.00 - 15.00
NL: 6.33E9
Base Peak F: FTMS + p ESI Full ms [70.0000-1050.0000] MS Pos-Ac-1
NL: 6.10E8
Base Peak m/z= 147.07408-147.07702 F: FTMS + p ESI Full ms [70.0000-1050.0000] MS Pos-Ac-1
NL: 5.41E7
Base Peak F: FTMS + p ESI d Full ms2 147.0755 MS Pos-Ac-1
Relative Abundance
Time (min)
Pos-Ac-1 #753 RT: 1.23 AV: 1 NL: 5.14E7
F: FTMS + p ESI d Full ms2 147.0755
84.04
130.05
102.05
147.08
m/z

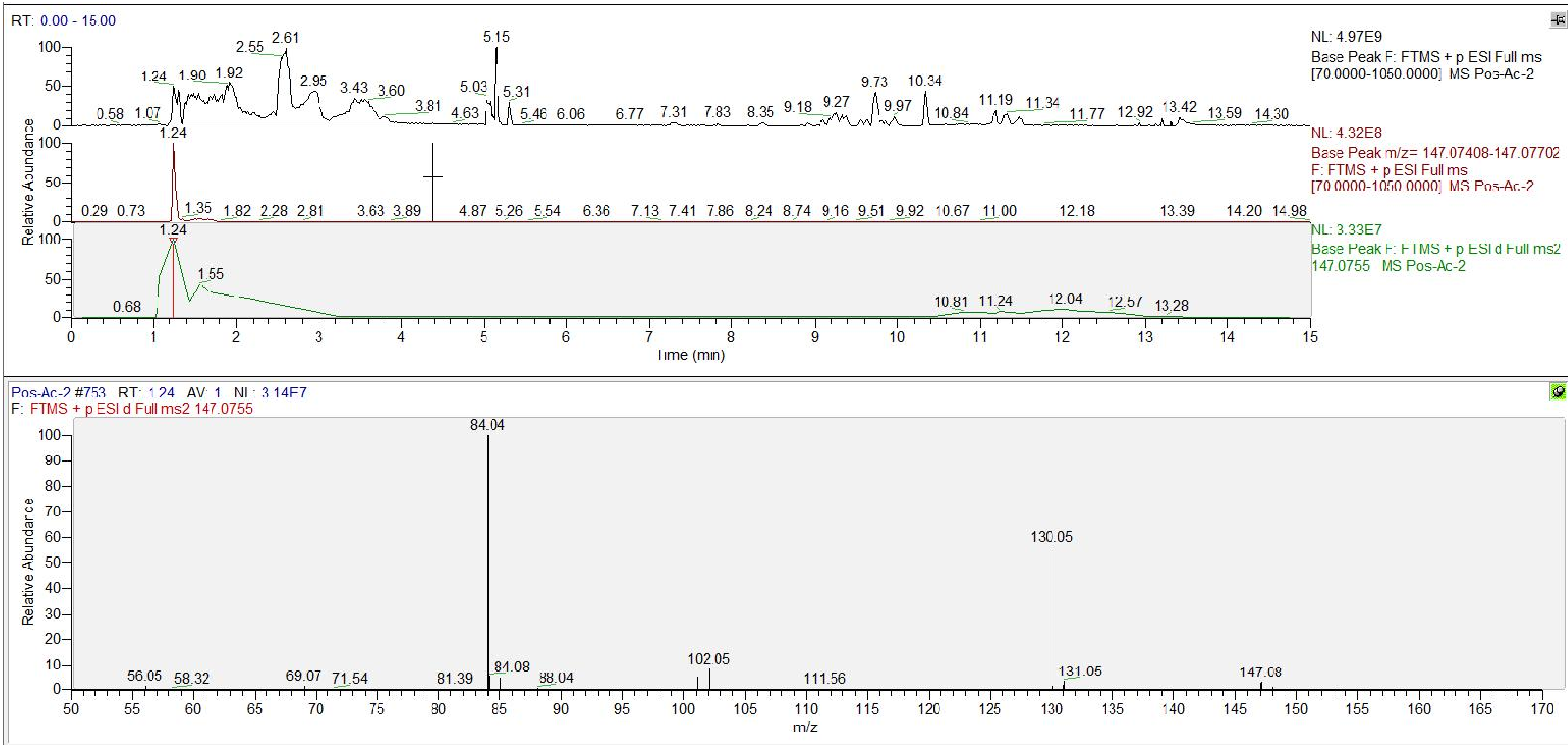

RT: 0.00 - 15.00
NL: 4.97E9
Base Peak F: FTMS + p ESI Full ms
[70.0000-1050.0000] MS Pos-Ac-2
NL: 4.32E8
Base Peak m/z= 147.07408-147.07702
F: FTMS + p ESI Full ms
[70.0000-1050.0000] MS Pos-Ac-2
NL: 3.33E7
Base Peak F: FTMS + p ESI d Full ms2
147.0755 MS Pos-Ac-2
Relative Abundance
Time (min)
Pos-Ac-2 #753 RT: 1.24 AV: 1 NL: 3.14E7
F: FTMS + p ESI d Full ms2 147.0755
84.04
130.05
102.05
147.08
131.05
84.08
88.04
111.56
81.39
69.07
71.54
56.05
58.32
Relative Abundance
m/z

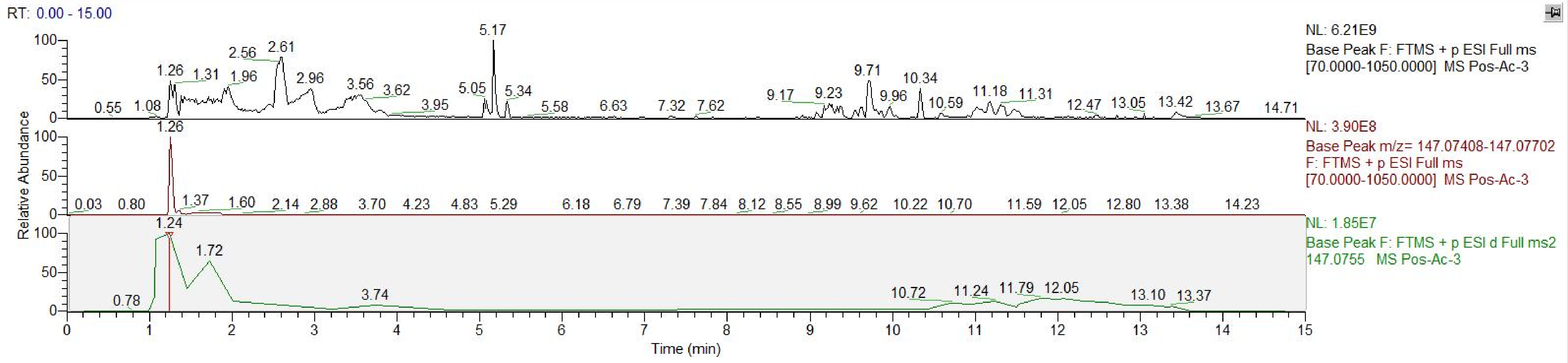
RT: 0.00 - 15.00
NL: 6.21E9
Base Peak F: FTMS + p ESI Full ms
[70.0000-1050.0000] MS Pos-Ac-3
NL: 3.90E8
Base Peak m/z= 147.07408-147.07702
F: FTMS + p ESI Full ms
[70.0000-1050.0000] MS Pos-Ac-3
NL: 1.85E7
Base Peak F: FTMS + p ESI d Full ms2
147.0755 MS Pos-Ac-3
Relative Abundance
Time (min)

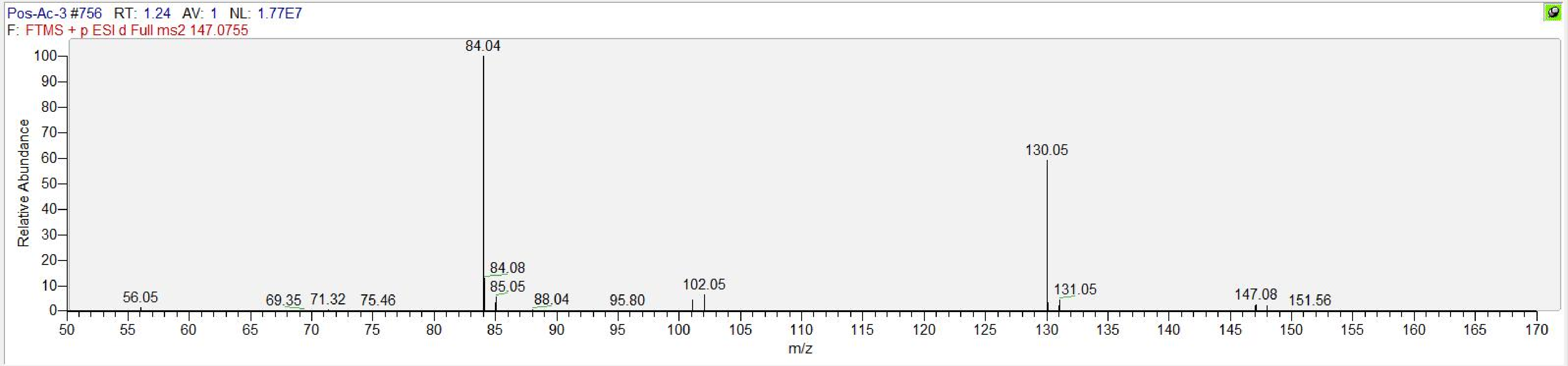
Pos-Ac-3 #756 RT: 1.24 AV: 1 NL: 1.77E7
F: FTMS + p ESI d Full ms2 147.0755
84.04
130.05
84.08
85.05
102.05
131.05
147.08
Relative Abundance
m/z

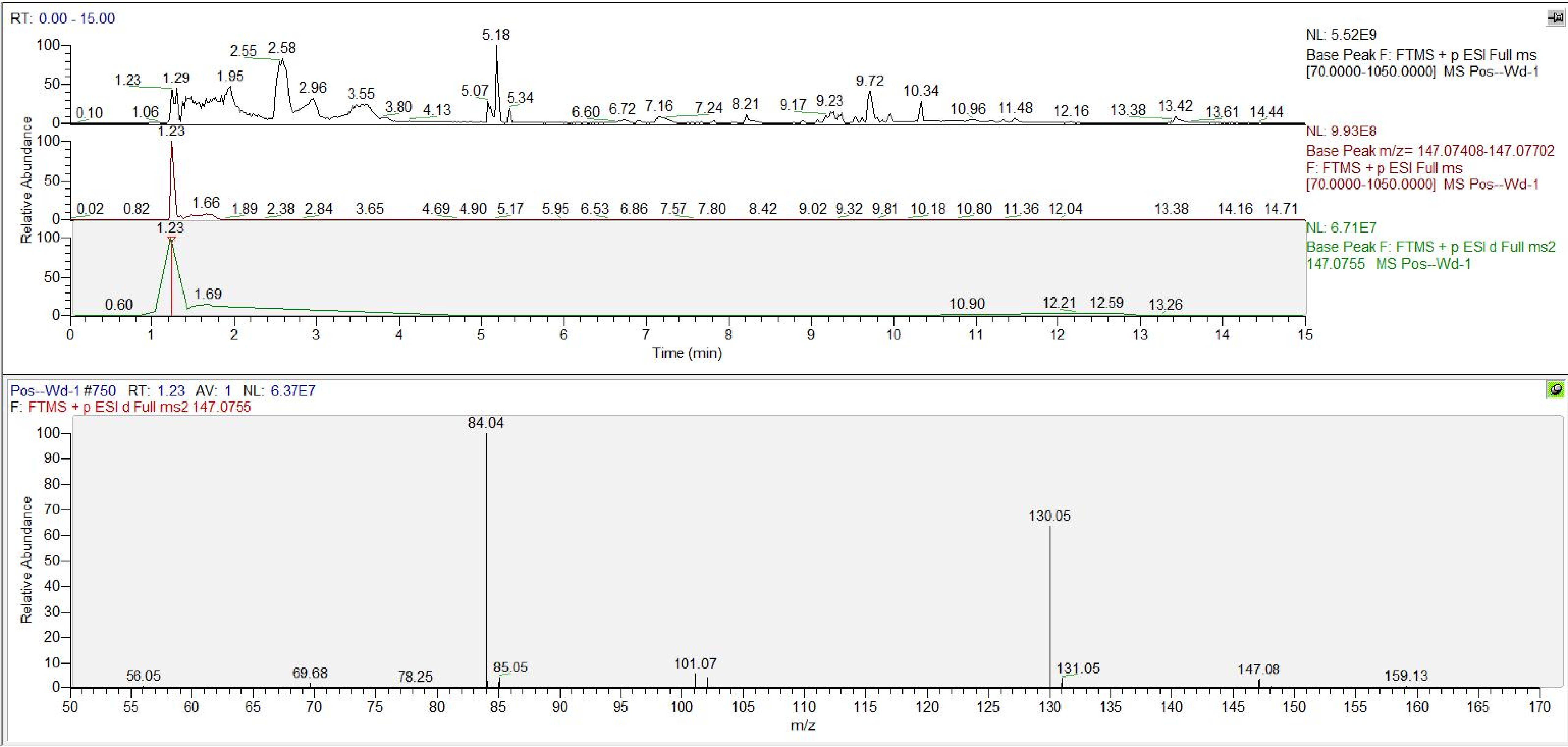
RT: 0.00 - 15.00
NL: 5.52E9
Base Peak F: FTMS + p ESI Full ms [70.0000-1050.0000] MS Pos--Wd-1
NL: 9.93E8
Base Peak m/z= 147.07408-147.07702 F: FTMS + p ESI Full ms [70.0000-1050.0000] MS Pos--Wd-1
NL: 6.71E7
Base Peak F: FTMS + p ESI d Full ms2 147.0755 MS Pos--Wd-1
Relative Abundance
Time (min)
Pos--Wd-1 #750 RT: 1.23 AV: 1 NL: 6.37E7
F: FTMS + p ESI d Full ms2 147.0755
84.04
130.05
101.07
147.08
m/z

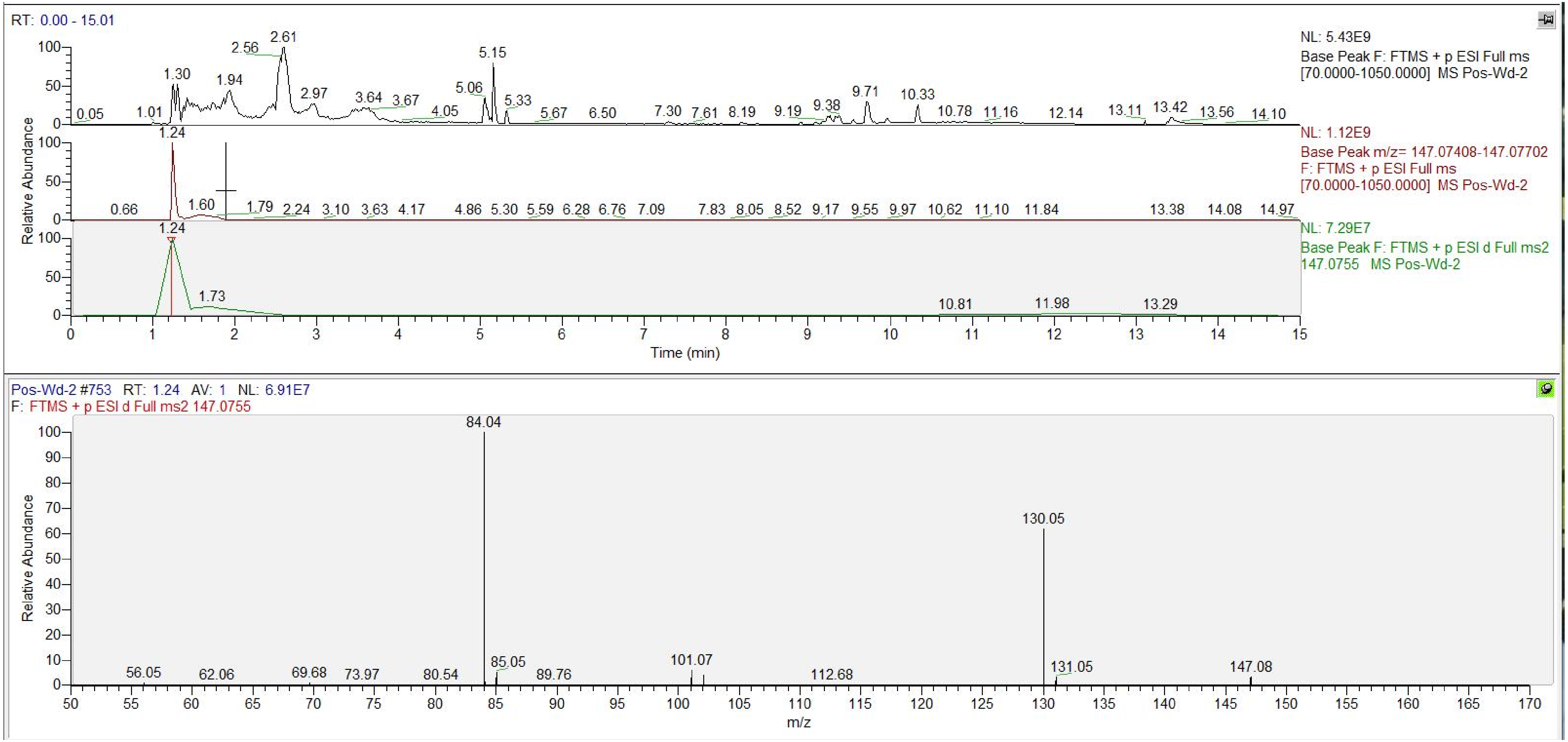
RT: 0.00 - 15.01
NL: 5.43E9
Base Peak F: FTMS + p ESI Full ms [70.0000-1050.0000] MS Pos-Wd-2
NL: 1.12E9
Base Peak m/z= 147.07408-147.07702 F: FTMS + p ESI Full ms [70.0000-1050.0000] MS Pos-Wd-2
NL: 7.29E7
Base Peak F: FTMS + p ESI d Full ms2 147.0755 MS Pos-Wd-2
Relative Abundance
Time (min)
Pos-Wd-2 #753 RT: 1.24 AV: 1 NL: 6.91E7
F: FTMS + p ESI d Full ms2 147.0755
84.04
130.05
56.05
62.06
69.68
73.97
80.54
85.05
89.76
101.07
112.68
131.05
147.08
m/z

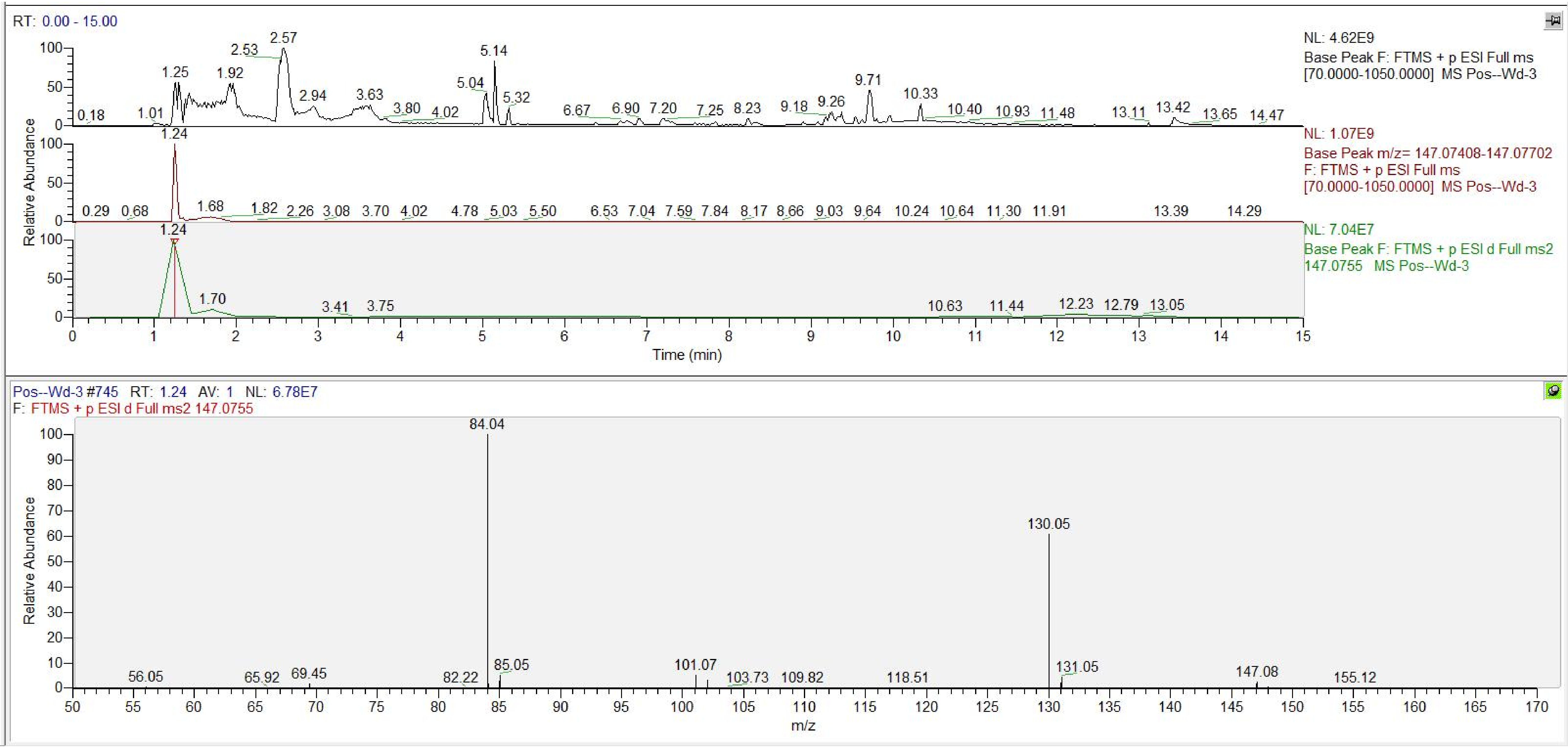
RT: 0.00 - 15.00
NL: 4.62E9
Base Peak F: FTMS + p ESI Full ms [70.0000-1050.0000] MS Pos--Wd-3
NL: 1.07E9
Base Peak m/z= 147.07408-147.07702 F: FTMS + p ESI Full ms [70.0000-1050.0000] MS Pos--Wd-3
NL: 7.04E7
Base Peak F: FTMS + p ESI d Full ms2 147.0755 MS Pos--Wd-3
Relative Abundance
Time (min)
Pos--Wd-3 #745 RT: 1.24 AV: 1 NL: 6.78E7
F: FTMS + p ESI d Full ms2 147.0755
84.04
130.05
85.05
101.07
131.05
147.08
56.05
65.92
69.45
82.22
103.73
109.82
118.51
155.12
m/z

Glutamine---- standard substance

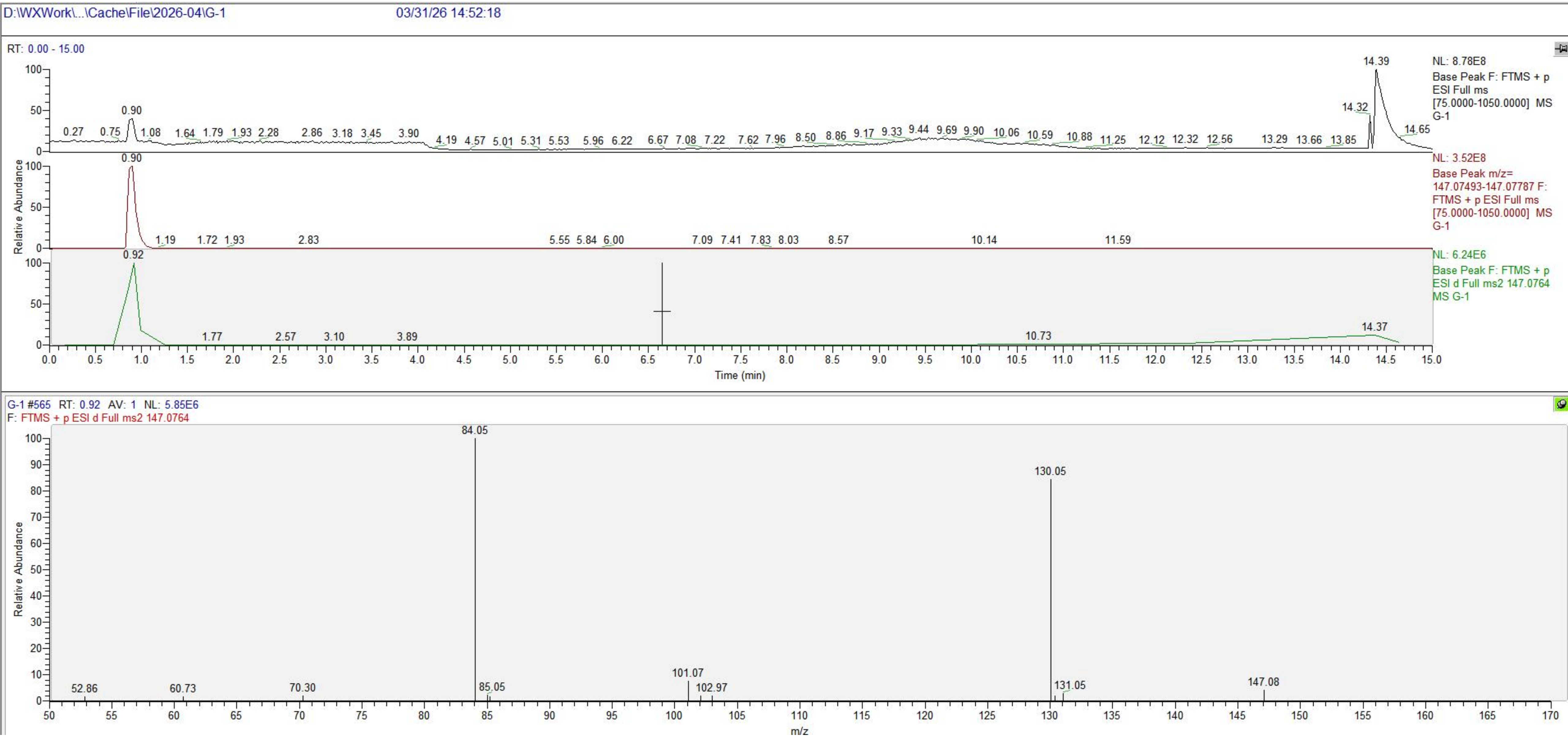

Supplement: Supplemental Figure 1.pdf [file IPHB_A_2668132_SM6108.pdf]
