# Supplementary material for: Wubie Fanchun Formula-inducible metabolites in primary ovarian insufficiency model mice that facilitate ovarian renovation
Source: Pharm Biol. 2026 May 12;64(1):725–48. doi: 10.1080/13880209.2026.2668132 (PMC13169455; doi:10.1080/13880209.2026.2668132)
Supplement: Supplemental Data.docx [file IPHB_A_2668132_SM6107.docx]

**Supplemental Table 1.** Experimental reagents and consumables

| Name | supplier | catalogue number |
| --- | --- | --- |
| Methanol | Merck | 34860 |
| Acetonitrile | Merck | [34851](https://www.sigmaaldrich.cn/CN/zh/product/sigald/34851) |
| 4-VCD | Sigma | 94956 |
| Wright Giemsa Stain Solution | Solarbio | G1020 |
| PBS（PH6.8） | Phygene Scientific | G4210 |
| Pentobarbital Sodium | Sigma | P3761 |
| 4% Paraformaldehyde | Beyotime | P0099 |
| E2 ELISA Kit | Nanjing Jiancheng Bioengineering Institute | H102-1-2 |
| FSH ELISA Kit | Nanjing Jiancheng Bioengineering Institute | H101-1-2 |
| AMH ELISA Kit | Nanjing Jiancheng Bioengineering Institute | H324-1-2 |
| Absolute Ethanol | Sinopharm | 100092683 |
| xylene | Sinopharm | 10023418 |
| Dewaxing liquid | Servicebio | G1128 |
| Neutral gum | Sinopharm | 10004160 |
| PAS Stain set | Servicebio | G1008 |
| General purpose tissue fixative | Servicebio | G1101 |
| Citric acid antigen repair solution（PH6.0) | Servicebio | G1202 |
| Name | supplier | catalogue number |
| EDTA Antigen Retrieval Solution | Servicebio | G1203 |
| PBS | Servicebio | G0002 |
| EDTA （PH8.0) | Servicebio | G1206 |
| Marker | Vazyme | MP102-01 |
| BSA | Servicebio | GC305010 |
| DAPI Staining reagents | Servicebio | G1012 |
| Anti-fluorescence quenching tablets | Servicebio | G1401 |
| Tissue autofluorescence quenching agent | Servicebio | G1221 |
| 0.9% Saline | Sichuan Kelun | - |
| Hematoxylin dyeing solution | Servicebio | G1004 |
| eosin dye | Servicebio | G1001 |
| ECL chemiluminescence substrate kit | Biosharp | BL520B |
| Antibody diluents | Beyotime | P0256 |
| Antibody eluent | Beyotime | P0025B |
| SDS-PAGE Protein loading buffer(5X) | Beyotime | P10015 |
| RIPA Lysate | Beyotime | P0013B |
| Skim milk powder | Biofroxx | 1172GR110 |
| Steel ball | Beyotime | F6621 |
| Slides (Paraffin slices) | Servicebio | G6012-1 |
| 1.5 mL Centrifuge tubes | Axygen | MCT-150-C |
| Anatomical instruments | Jinhuan medical | - |
| Cryostored tubes | Corning | - |
| Slides | Servicebio | WG6012 |
| Coverslip | Citotest Labware Manufacturing Co., Ltd | 10212432C |

**Supplemental Table 2.** antibodies information

| Name | supplier | catalogue number | dilution | species | RRID number |
| --- | --- | --- | --- | --- | --- |
| Goat Anti-Rabbit IgG (H+L) HRP | Affinity | S0001 | 1:5000 | Goat | AB_2839429 |
| Goat Anti-Mouse IgG (H+L) HRP | Affinity | S0002 | 1:5000 | Goat | AB_2839430 |
| Anti-AKT | Cell Signaling Technology | 4691 | 1:1000 | Rabbit | AB_915783 |
| Anti-phospho-AKT | Cell Signaling Technology | 4060 | 1:2000 | Rabbit | AB_2315049 |
| Anti-PI3K | Proteintech | 20584-1-AP | 1:1000 | Rabbit | AB_10734439 |
| Anti-PI3K | Cell Signaling Technology | 4249T | 1:1000 | Rabbit | - |
| Name | supplier | catalogue number | dilution | species | RRID number |
| Anti-phospho-PI3K | Abmart | T40116 | 1:1000 | Rabbit | AB_2936325 |
| Anti-phospho-PI3K | Cell Signaling Technology | 4228 | 1:1000 | Rabbit | AB_659940 |
| Anti-FOXO3A | Proteintech | 10849-1-AP | 1:5000 | Rabbit | AB_2247214 |
| Anti-FOXO3A | Proteintech | 66428-1-Ig | 1:5000 | Mouse | AB_2881799 |
| Anti-phospho-FOXO3A | Proteintech | 28755-1-AP | 1:1000 | Rabbit | AB_2881210 |

**Supplemental Table 3.** Summary of Drug Targets for Wubie Fanchun Oral Liquid.

| **Gene Symbol** | **Uniprot ID** |
| --- | --- |
| AKR1B1 | P15121 |
| TNF | P01375 |
| IL2 | P60568 |
| ADORA1 | P30542 |
| XDH | P47989 |
| RPS6KA3 | P51812 |
| EGFR | P00533 |
| CA2 | P00918 |
| ACHE | P22303 |
| CA4 | P22748 |
| NQO2 | P16083 |
| PTGS2 | P35354 |
| NOX4 | Q9NPH5 |
| ADRA2C | P18825 |
| ALDH2 | P05091 |
| NMUR2 | Q9GZQ4 |
| ADRA2A | P08913 |
| SQLE | Q14534 |
| CA7 | P43166 |
| CA12 | O43570 |
| ADH1A | P07327 |
| ADH1B | P00325 |
| ADH1C | P00326 |
| MCL1 | Q07820 |
| NOS2 | P35228 |
| CES1 | P23141 |
| CHRM4 | P08173 |
| CHRNA4 | P43681 |
| CHRNB2 | P17787 |
| CHRM5 | P08912 |
| CHRM2 | P08172 |
| CHRM1 | P11229 |
| CHRM3 | P20309 |
| CHRNA7 | P36544 |
| CES2 | O00748 |
| CA1 | P00915 |
| CYP11B1 | P15538 |
| CYP11B2 | P19099 |
| MGLL | Q99685 |
| PDE5A | O76074 |
| CD38 | P28907 |
| ACLY | P53396 |
| FDFT1 | P37268 |
| EGLN1 | Q9GZT9 |
| HMGCR | P04035 |
| KDM4E | B2RXH2 |
| KDM6B | O15054 |
| ENGASE | Q8NFI3 |
| SELL | P14151 |
| SELP | P16109 |
| LGALS3 | P17931 |
| CA9 | Q16790 |
| OGA | O60502 |
| FOLH1 | Q04609 |
| NAALAD2 | Q9Y3Q0 |
| NEU2 | Q9Y3R4 |
| CACNA2D1 | P54289 |
| SLC7A5 | Q01650 |
| GABBR2 | O75899 |
| GABBR1 | Q9UBS5 |
| ADORA3 | P0DMS8 |
| FYN | P06241 |
| TACR1 | P25103 |
| TH | P07101 |
| KDM4C | Q9H3R0 |
| LCK | P06239 |
| ADRA2B | P18089 |
| ADRA1A | P35348 |
| CPB2 | Q96IY4 |
| ANPEP | P15144 |
| ENPEP | Q07075 |
| KDM1A | O60341 |
| GABRR1 | P24046 |
| CPA3 | P15088 |
| SLC6A2 | P23975 |
| SLC6A4 | P31645 |
| TAAR1 | Q96RJ0 |
| SLC15A1 | P46059 |
| PEPD | P12955 |
| THRA | P10827 |
| THRB | P10828 |
| NR1H4 | Q96RI1 |
| APEX1 | P27695 |
| PTPRA | P18433 |
| KMO | O15229 |
| GABBR2 | O75899 |
| MAPKAPK2 | P49137 |
| CPB1 | P15086 |
| KIF11 | P52732 |
| ESR1 | P03372 |
| ESR2 | Q92731 |
| GRB2 | P62993 |
| ADORA2A | P29274 |
| CCNE1 | P24864 |
| CDK2 | P24941 |
| ADORA2B | P29275 |
| GDA | Q9Y2T3 |
| SLC22A6 | Q4U2R8 |
| ITGA2B | P08514 |
| ITGB3 | P05106 |
| F2 | P00734 |
| LTA4H | P09960 |
| ACE | P12821 |
| EGLN3 | Q9H6Z9 |
| REN | P00797 |
| PLA2G2A | P14555 |
| PLA2G5 | P39877 |
| SLC1A2 | P43004 |
| HCAR2 | Q8TDS4 |
| DPP4 | P27487 |
| XIAP | P98170 |
| SLC5A1 | P13866 |
| DLG4 | P78352 |
| LAP3 | P28838 |
| VEGFA | P15692 |
| FGF1 | P05230 |
| HPSE | Q9Y251 |
| CDK1 | P06493 |
| FGF2 | P09038 |
| LGALS4 | P56470 |
| LGALS8 | O00214 |
| HSP90AA1 | P07900 |
| PSEN2 | P49810 |
| PSENEN | Q9NZ42 |
| NCSTN | Q92542 |
| APH1A | Q96BI3 |
| PSEN1 | P49768 |
| APH1B | Q8WW43 |
| HTR2B | P41595 |
| DRD1 | P21728 |
| DRD2 | P14416 |
| ADRA1D | P25100 |
| HTR2A | P28223 |
| HTR2C | P28335 |
| DRD3 | P35462 |
| CYP2D6 | P10635 |
| HTR6 | P50406 |
| HTR1B | P28222 |
| RORC | P51449 |
| STAT3 | P40763 |
| TRPV1 | Q8NER1 |
| NOS1 | P29475 |
| GBA1 | P04062 |
| PYGB | P11216 |
| PYGM | P11217 |
| SLC1A1 | P43005 |
| GRM4 | Q14833 |
| GRM5 | P41594 |
| GRM8 | O00222 |
| GRM1 | Q13255 |
| GRM7 | Q14831 |
| GRIK1 | P39086 |
| GABBR2 | O75899 |
| GRIK2 | Q13002 |
| GRM3 | Q14832 |
| GRM6 | O15303 |
| GRM2 | Q14416 |
| GABRA1 | P14867 |
| GABRB2 | P47870 |
| GABRG2 | P18507 |
| SLC6A11 | P48066 |
| SLC6A13 | Q9NSD5 |
| OAT | P04181 |
| GRIA1 | P42261 |
| GRIA4 | P48058 |
| PLG | P00747 |
| GRIK5 | Q16478 |
| GRIK3 | Q13003 |
| GRIA2 | P42262 |
| BBOX1 | O75936 |
| TYR | P14679 |
| SLC5A2 | P31639 |
| ADA | P00813 |
| CA14 | Q9ULX7 |
| HRAS | P01112 |
| LGALS9 | O00182 |
| ADK | P55263 |
| CA13 | Q8N1Q1 |
| CCNA1 | P78396 |
| CCNA2 | P20248 |
| HK2 | P52789 |
| HK1 | P19367 |
| CA5A | P35218 |
| SLC29A1 | Q99808 |
| EPHX2 | P34913 |
| IGFBP3 | P17936 |
| CA6 | P23280 |
| GAPDH | P04406 |
| MME | P08473 |
| TYMP | P19971 |
| GBA | P04062 |
| MGAM | O43451 |
| MMP13 | P45452 |
| MMP1 | P03956 |
| MMP7 | P09237 |
| MMP12 | P39900 |
| MMP8 | P22894 |
| PNP | P00491 |
| AKR1C3 | P42330 |
| HSPA8 | P11142 |
| SLC5A4 | Q9NY91 |
| GRK1 | Q15835 |
| MAG | P20916 |
| GAA | P10253 |
| EDNRA | P25101 |
| ABL1 | P00519 |
| EPHA2 | P29317 |
| SRC | P12931 |
| MAP3K9 | P80192 |
| FGFR1 | P11362 |
| CDA | P32320 |
| AURKA | O14965 |
| BTK | Q06187 |
| ERN1 | O75460 |
| DAO | P14920 |
| ATIC | P31939 |
| ALOX12 | P18054 |
| BCL2L1 | Q07817 |
| PTPA | Q15257 |
| GLI1 | P08151 |
| PPP1CC | P36873 |
| GLRA1 | P23415 |
| GLRA2 | P23416 |
| TNNC1 | P63316 |
| TNNT2 | P45379 |
| TNNI3 | P19429 |
| ELANE | P08246 |
| SLC28A3 | Q9HAS3 |
| PTPN1 | P18031 |
| HSD11B2 | P80365 |
| HSD11B1 | P28845 |
| MMP9 | P14780 |
| JUN | P05412 |
| AR | P10275 |
| MMP2 | P08253 |
| PTGER4 | P35408 |
| PTGER2 | P43116 |
| PIM1 | P11309 |
| CSNK2A1 | P68400 |
| PTP4A3 | O75365 |
| FNTA | P49354 |
| FNTB | P49356 |
| BCL2 | P10415 |
| FTO | Q9C0B1 |
| LIMK1 | P53667 |
| CYP19A1 | P11511 |
| ABCB1 | P08183 |
| BCHE | P06276 |
| CYP1B1 | Q16678 |
| AURKB | Q96GD4 |
| KDR | P35968 |
| PLK1 | P53350 |
| MET | P08581 |
| AXL | P30530 |
| FASN | P49327 |
| PARP1 | P09874 |
| TNKS2 | Q9H2K2 |
| TNKS | O95271 |
| CRHR1 | P34998 |
| CDK5R1 | Q15078 |
| CDK5 | Q00535 |
| CCNB3 | Q8WWL7 |
| CCNB1 | P14635 |
| CCNB2 | O95067 |
| CDK6 | Q00534 |
| ABCG2 | Q9UNQ0 |
| CBR1 | P16152 |
| TBXAS1 | P24557 |
| ESRRA | P11474 |
| ESRRB | O95718 |
| LDHA | P00338 |
| LDHB | P07195 |
| FLT3 | P36888 |
| SYK | P43405 |
| GSK3B | P49841 |
| ABCC1 | P33527 |
| TTR | P02766 |
| CFTR | P13569 |
| AKR1B10 | O60218 |
| SULT2B1 | O00204 |
| BCAT2 | O15382 |
| PLA2G10 | O15496 |
| PAPSS1 | O43252 |
| GSR | P00390 |
| OTC | P00480 |
| HPRT1 | P00492 |
| C1R | P00736 |
| IGLV2-8 | P01709 |
| APCS | P02743 |
| SOD2 | P04179 |
| TK1 | P04183 |
| IGF1 | P01343 |
| REG1A | P05451 |
| PYGL | P06737 |
| GP1BA | P07359 |
| C8G | P07360 |
| HEXB | P07686 |
| APRT | P07741 |
| HSPA1A | P08107 |
| HSPA1B | P08107 |
| AMY1B | P04745 |
| AMY1C | P04745 |
| AMY1A | P04745 |
| RNASE2 | P10153 |
| UMPS | P11172 |
| MTHFD1 | P11586 |
| RNASE3 | P12724 |
| HSD17B1 | P14061 |
| RAC2 | P15153 |
| ABO | Q9NY01 |
| GM2A | P17900 |
| SRM | P19623 |
| SDS | P20132 |
| FECH | P22830 |
| WARS1 | P23381 |
| ITPKA | P23677 |
| BLVRB | P30043 |
| RNASE4 | P34096 |
| CBS | P35520 |
| PCK1 | P35558 |
| MAPK8 | P45983 |
| GNPDA1 | P46926 |
| FHIT | P49789 |
| CDK7 | P50613 |
| RHOA | P61586 |
| LYZ | P61626 |
| PPIA | P62937 |
| ADAM17 | P78536 |
| ARF1 | P84077 |
| PITPNA | Q00169 |
| PPARD | Q03181 |
| ARHGAP1 | Q07960 |
| ITK | Q08881 |
| MTAP | Q13126 |
| CHIT1 | Q13231 |
| RHEB | Q15382 |
| MAPK14 | Q16539 |
| GSTA3 | Q16772 |
| HAGH | Q16775 |
| ERI1 | Q8IV48 |
| CANT1 | Q8WVQ1 |
| KAT2B | Q92831 |
| BHMT | Q93088 |
| BIRC7 | Q96CA5 |
| PPCDC | Q96CD2 |
| NMNAT3 | Q96T66 |
| NUDT9 | Q9BW91 |
| UCK2 | Q9BZX2 |
| PLEKHA4 | Q9H4M7 |
| NMNAT1 | Q9HAN9 |
| TREM1 | Q9NP99 |
| NT5M | Q9NPB1 |
| PNPO | Q9NVS9 |
| CTSF | Q9UBX1 |
| MAN1B1 | Q9UKM7 |
| ARL5A | Q9Y689 |
| CHK1 | O14757 |
| KAT1 | O14929 |
| STK6 | O14965 |
| PDPK1 | O15530 |
| GET3 | O43681 |
| CCNT1 | O60563 |
| JAK2 | O60674 |
| CATL2 | O60911 |
| NR1I2 | O75469 |
| AMH | P03971 |
| RAF1 | P04049 |
| ALDOA | P04075 |
| SHBG | P04278 |
| CD1A | P06126 |
| INSR | P06213 |
| IGF1R | P08069 |
| NR3C2 | P08235 |
| MMP3 | P08254 |
| HCK | P08631 |
| GSTP1 | P09211 |
| GSTM1 | P09488 |
| C1S | P09871 |
| GSTT2 | P0CG29 |
| GSTT2 | P0CG30 |
| KIT | P10721 |
| ACADM | P11310 |
| ADHX | P11766 |
| CCL5 | P13501 |
| ICAM2 | P13598 |
| MIF | P14174 |
| BRAF | P15056 |
| FABP4 | P15090 |
| ARSA | P15289 |
| NQO1 | P15559 |
| NPR3 | P17342 |
| TPH1 | P17752 |
| BMP7 | P18075 |
| RXRA | P19793 |
| RAB5A | P20339 |
| AHCY | P23526 |
| CMA1 | P23946 |
| FCAR | P24071 |
| DDX6 | P26196 |
| DCK | P27707 |
| GSTM2 | P28161 |
| IMPA1 | P29218 |
| NOS3 | P29474 |
| AKT1 | P31749 |
| CDD | P32320 |
| PPARG | P37231 |
| STAT1 | P42224 |
| CASP3 | P42574 |
| ZAP70 | P43403 |
| CLK1 | P49759 |
| HINT1 | P49773 |
| FABP6 | P51161 |
| MMP16 | P51512 |
| GALK1 | P51570 |
| JAK3 | P52333 |
| DAPK1 | P53355 |
| EPHB4 | P54760 |
| NR1H2 | P55055 |
| CASP7 | P55210 |
| BACE1 | P56817 |
| NOS2 | P60321 |
| NANOS3 | P60323 |
| CDC42 | P60953 |
| TGFB2 | P61812 |
| NCS1 | P62166 |
| RAN | P62826 |
| GSTO1 | P78417 |
| LCN2 | P80188 |
| PTK2 | Q05397 |
| PDE4B | Q07343 |
| PPARA | Q07869 |
| ACK1 | Q07912 |
| TGM3 | Q08188 |
| PDE4D | Q08499 |
| BST1 | Q10588 |
| PDE3B | Q13370 |
| GALE | Q14376 |
| PDK2 | Q15119 |
| ERBB4 | Q15303 |
| QPCT | Q16769 |
| SETD7 | Q8WTS6 |
| ISG20 | Q96AZ6 |
| BAG1 | Q99933 |
| CATS | Q9BSJ6 |
| HDAC8 | Q9BY41 |
| RTN4R | Q9BZR6 |
| CD209 | Q9NNX6 |
| PAK6 | Q9NQU5 |
| ALDR | Q9UBJ2 |
| PADI4 | Q9UM07 |
| HEM2 | Q9Y2A7 |
| ST14 | Q9Y5Y6 |

**Supplemental Table 4.** Summary of Disease Targets for POI.

| **Gene Name** | **Uniprot ID** |
| --- | --- |
| AKR1C3 | P42330 |
| HSD17B1 | P14061 |
| eryF | Q00441 |
| HSD3B1 | P14060 |
| CYP3A4 | P08684 |
| BRCA1 | P38398 |
| BRCA2 | P51587 |
| TP53 | P04637 |
| RAD51C | O43502 |
| FMR1 | Q06787 |
| ATM | Q13315 |
| NR5A1 | Q13285 |
| FSHR | P23945 |
| NBN | O60934 |
| RAD51D | O75771 |
| FOXL2 | P58012 |
| FANCM | Q8IYD8 |
| NOBOX | O60393 |
| BMP15 | O95972 |
| CHEK2 | O96017 |
| STAG3 | Q9UJ98 |
| WT1 | P19544 |
| BRIP1 | Q9BX63 |
| PTEN | P60484 |
| POF1B | Q8WVV4 |
| CYP11A1 | P05108 |
| ERCC6 | Q03468 |
| PALB2 | Q86YC2 |
| TP63 | Q9H3D4 |
| GDF9 | O60383 |
| FANCA | O15360 |
| MCM9 | Q9NXL9 |
| CYP19A1 | P11511 |
| CDH1 | P12830 |
| CYP17A1 | P05093 |
| GALT | P07902 |
| MSH4 | O15457 |
| BNC1 | Q01954 |
| FIGLA | Q6QHK4 |
| PMS2 | P54278 |
| CTNNB1 | P35222 |
| MSH2 | P43246 |
| AKT1 | P31749 |
| PIK3CA | P42336 |
| WRN | Q14191 |
| KDR | P35968 |
| LHCGR | P22888 |
| AFP | P02771 |
| PDGFRB | P09619 |
| EIF2B2 | P49770 |
| MLH3 | Q9UHC1 |
| AR | P10275 |
| MSH6 | P52701 |
| ERBB2 | P04626 |
| POMC | P01189 |
| ERCC1 | P07992 |
| C11orf65 | Q8NCR3 |
| NTRK1 | P04629 |
| KRAS | P01116 |
| POLG | P54098 |
| DIAPH2 | O60879 |
| LMNA | P02545 |
| BARD1 | Q99728 |
| MRE11 | P49959 |
| NOS3 | P29474 |
| BLM | P54132 |
| BUB1B | O60566 |
| MCM8 | Q9UJA3 |
| EGFR | P00533 |
| HFM1 | A2PYH4 |
| XRCC2 | O43543 |
| RECQL4 | O94761 |
| MLH1 | P40692 |
| PMM2 | O15305 |
| THBS1 | P07996 |
| RAD50 | Q92878 |
| IL6 | P05231 |
| RAD54L | Q92698 |
| CDKN2A | Q8N726 |
| ESR1 | P03372 |
| AFF2 | P51816 |
| JAK2 | O60674 |
| KIT | P10721 |
| POR | P16435 |
| BMP6 | P22004 |
| KHDRBS1 | Q07666 |
| FBN1 | P35555 |
| VEGFA | P15692 |
| AMH | P03971 |
| NOTCH2 | Q04721 |
| TNF | P01375 |
| MECOM | Q03112 |
| HNF1B | P35680 |
| DICER1 | Q9UPY3 |
| INHA | P05111 |
| PRLR | P16471 |
| APC | P25054 |
| TSC2 | P49815 |
| MSH5 | O43196 |
| PTCH1 | Q13635 |
| RET | P07949 |
| PURA | Q00577 |
| FANCD2 | Q9BXW9 |
| MRPS22 | P82650 |
| REC8 | O95072 |
| BRAF | P15056 |
| ERCC4 | Q92889 |
| PEX6 | Q13608 |
| CLPB | Q9H078 |
| IGF2R | P11717 |
| GGPS1 | O95749 |
| NUP107 | P57740 |
| ERCC2 | P18074 |
| POLGARF | A0A3B3IS91 |
| LARS2 | Q15031 |
| ESR2 | Q92731 |
| CDKN1B | P46527 |
| C14orf39 | Q8N1H7 |
| RB1 | P06400 |
| NR0B1 | P51843 |
| FANCC | Q00597 |
| PDGFRA | P16234 |
| SPIDR | Q14159 |
| IGF1 | P05019 |
| SRD5A2 | P31213 |
| STAR | P49675 |
| INS | P01308 |
| HSF2BP | O75031 |
| FANCG | O15287 |
| NR5A2 | O00482 |
| RYR3 | Q15413 |
| ERCC3 | P19447 |
| PSMC3IP | Q9P2W1 |
| NF1 | P21359 |
| PREPL | Q4J6C6 |
| MRPS7 | Q9Y2R9 |
| SYCE1 | Q8N0S2 |
| FGFR2 | P21802 |
| RAD51 | Q06609 |
| DMRT3 | Q9NQL9 |
| GNRH1 | P01148 |
| CYP21A2 | P08686 |
| SGO2 | Q562F6 |
| LRRC41 | Q15345 |
| TERT | O14746 |
| BMPR1A | P36894 |
| PRKN | O60260 |
| SIRT6 | Q8N6T7 |
| SERPINE1 | P05121 |
| TSHR | P16473 |
| TSC1 | Q92574 |
| ZNF462 | Q96JM2 |
| PGR | P06401 |
| AARS2 | Q5JTZ9 |
| ADAMTS1 | Q9UHI8 |
| CRP | P02741 |
| HROB | Q8N3J3 |
| RICTOR | Q6R327 |
| SH2B1 | Q9NRF2 |
| ALB | P02768 |
| FGFR3 | P22607 |
| ERCC5 | P28715 |
| MET | P08581 |
| MT-CYB | P00156 |
| MND1 | Q9BWT6 |
| MUC16 | Q8WXI7 |
| FGFR1 | P11362 |
| CXCL8 | P10145 |
| INSL6 | Q9Y581 |
| TUG1 | A0A6I8PU40 |
| DPPA2 | Q7Z7J5 |
| MMP2 | P08253 |
| FANCE | Q9HB96 |
| MAP3K1 | Q13233 |
| BRDT | Q58F21 |
| BRWD1 | Q9NSI6 |
| TG | P01266 |
| ALK | Q9UM73 |
| LEP | P41159 |
| ADAMTS6 | Q9UKP5 |
| ATG9B | Q674R7 |
| IL10 | P22301 |
| MUTYH | Q9UIF7 |
| PTPN11 | Q06124 |
| AOPEP | Q8N6M6 |
| TGFB1 | P01137 |
| XPC | Q01831 |
| AXIN2 | Q9Y2T1 |
| FANCF | Q9NPI8 |
| AMHR2 | Q16671 |
| HSD17B4 | P51659 |
| REN | P00797 |
| PRL | P01236 |
| HNF1A | P20823 |
| HRAS | P01112 |
| SMARCB1 | Q12824 |
| ACE | P12821 |
| ABCA3 | Q99758 |
| MMP9 | P14780 |
| XPA | P23025 |
| CGA | P01215 |
| ABCD1 | P33897 |
| FH | P07954 |
| CTNNA1 | P35221 |
| TTN | Q8WZ42 |
| EXT2 | Q93063 |
| CFTR | P13569 |
| SMARCA4 | P51532 |
| FAS | P25445 |
| HIF1A | Q16665 |
| PPM1D | O15297 |
| MTOR | P42345 |
| TWNK | Q96RR1 |
| TGFBR2 | P37173 |
| AIRE | O43918 |
| FLCN | Q8NFG4 |
| STK11 | Q15831 |
| CDKN2B | P42772 |
| MMP1 | P03956 |
| BCL2 | P10415 |
| MSR1 | P21757 |
| PGBD3 | Q8N328 |
| IL1B | P01584 |
| MYC | P01106 |
| EIF2B4 | Q9UI10 |
| EXT1 | Q16394 |
| IL2 | P60568 |
| GATA4 | P43694 |
| POLR3H | Q9Y535 |
| FLT1 | P17948 |
| SHBG | P04278 |
| CASP3 | P42574 |
| HMMR | O75330 |
| SFTPB | P07988 |
| PARP1 | P09874 |
| VIM | P08670 |
| RNASEL | Q05823 |
| MUC1 | P15941 |
| STAT3 | P40763 |
| SOX9 | P48436 |
| ANGPT2 | O15123 |
| AAAS | Q9NRG9 |
| IGFBP3 | P17936 |
| SOHLH1 | Q5JUK2 |
| TWIST1 | Q15672 |
| MDM2 | Q00987 |
| FGF2 | P09038 |
| ZSWIM7 | Q19AV6 |
| DROSHA | Q9NRR4 |
| FASLG | P48023 |
| CSF3 | P09919 |
| HSD3B2 | P26439 |
| MAPK1 | P28482 |
| IFNG | P01579 |
| SIRT1 | Q96EB6 |
| RRAS2 | P62070 |
| RYR1 | P21817 |
| TLR4 | O00206 |
| IGF2 | P01344 |
| RECK | O95980 |
| SMAD4 | Q13485 |
| WNT4 | P56705 |
| IL1A | P01583 |
| ELN | P15502 |
| TGFBR1 | P36897 |
| ABCB1 | P08183 |
| ZNF276 | Q8N554 |
| MYH11 | P35749 |
| CLPP | Q16740 |
| CDKN1A | P38936 |
| POLR1C | O15160 |
| RECQL | P46063 |
| TIMP1 | P01033 |
| GNAS | P84996 |
| PGRMC1 | O00264 |
| EDN1 | P05305 |
| IGFBP1 | P08833 |
| EPO | P01588 |
| FOXO3 | O43524 |
| BMP4 | P12644 |
| SRA1 | Q9HD15 |
| INSR | P06213 |
| EGF | P01133 |
| SMAD6 | O43541 |
| RAD51B | O15315 |
| POU5F1 | Q01860 |
| COL2A1 | P02458 |
| MIR22HG | Q0VDD5 |
| CARS2 | Q9HA77 |
| VDR | P11473 |
| PTGS2 | P35354 |
| MTHFR | P42898 |
| KITLG | P21583 |
| TBX5 | Q99593 |
| HOXA11 | P31270 |
| APOB | P04114 |
| CAV1 | Q03135 |
| SERPINH1 | P50454 |
| NFKB1 | P19838 |
| ABRAXAS1 | Q6UWZ7 |
| PPARG | P37231 |
| WFDC2 | Q14508 |
| VHL | P40337 |
| FSHB | P01225 |
| F2 | P00734 |
| HLA-DRB1 | P01911 |
| BMPR1B | O00238 |
| JUN | P05412 |
| COL1A1 | P02452 |
| KRT7 | P08729 |
| CTLA4 | P16410 |
| CCL2 | P13500 |
| EIF2B5 | Q13144 |
| TIMP2 | P16035 |
| GNRHR | P30968 |
| EXO1 | Q9UQ84 |
| BAX | Q07812 |
| SPP1 | P10451 |
| COMT | P21964 |
| APOA1 | P02647 |
| ZP3 | P21754 |
| APOE | P02649 |
| IGF1R | P08069 |
| TGFB3 | P10600 |
| CYP11B1 | P15538 |
| ERAL1 | O75616 |
| MC2R | Q01718 |
| ITGB3 | P05106 |
| SMAD3 | P84022 |
| ENG | P17813 |
| SMAD5-AS1 | Q9Y6J3 |
| PLAU | P00749 |
| CRH | P06850 |
| IL18 | Q14116 |
| ATAD3A | Q9NVI7 |
| NOTCH1 | P46531 |
| TGFB2 | P61812 |
| DSTYK | Q6XUX3 |
| MMP14 | P50281 |
| PTH | P01270 |
| POLE | Q07864 |
| IL4 | P05112 |
| FN1 | P02751 |
| PAX2 | Q02962 |
| MMP8 | P22894 |
| SPINK1 | P00995 |
| MMP3 | P08254 |
| DAZL | Q92904 |
| PLG | P00747 |
| GH1 | P01241 |
| FXR1 | P51114 |
| BMP2 | P12643 |
| DES | P17661 |
| BGLAP | P02818 |
| HARS2 | P49590 |
| CDKN1C | P49918 |
| LDLR | P01130 |
| LRP5 | O75197 |
| SOD2 | P04179 |
| VRK1 | Q99986 |
| SLC27A4 | Q6P1M0 |
| GLI3 | P10071 |
| CXCL12 | P48061 |
| GSTP1 | P09211 |
| PTPN22 | Q9Y2R2 |
| EP300 | Q09472 |
| STAT1 | P42224 |
| ZMPSTE24 | O75844 |
| F5 | P12259 |
| NDE1 | Q9NXR1 |
| ADIPOQ | Q15848 |
| FANCL | Q9NW38 |
| BDNF | P23560 |
| MYH7 | P12883 |
| H2AC18 | Q6FI13 |
| ERBB3 | P21860 |
| AKT2 | P31751 |
| IL17A | Q16552 |
| ESRRB | O95718 |
| TYMS | P04818 |
| GALK1 | P51570 |
| FST | P19883 |
| CDK4 | P11802 |
| F3 | P13726 |
| MITF | O75030 |
| SOD1 | P00441 |
| ALPL | P05186 |
| BPESC1 | Q9GZL8 |
| CBL | P22681 |
| B2M | P61769 |
| SCN5A | Q14524 |
| CCND1 | P24385 |
| TEK | Q02763 |
| SERPINC1 | P01008 |
| CSF2 | P04141 |
| ATR | Q13535 |
| SRY | Q05066 |
| WDR19 | Q8NEZ3 |
| NFKB2 | Q00653 |
| CASP8 | Q14790 |
| DAB2 | P98082 |
| BCL2L1 | Q07817 |
| GAPDH | P04406 |
| LCN2 | P80188 |
| CHD7 | Q9P2D1 |
| SGPL1 | O95470 |
| RIPK1 | Q13546 |
| BMPR2 | Q13873 |
| CDK2 | P24941 |
| ICAM1 | P05362 |
| MAPK14 | Q16539 |
| BIRC5 | O15392 |
| GLI1 | P08151 |
| COL3A1 | P02461 |
| KISS1 | Q15726 |
| GJA1 | P17302 |
| IGFBP2 | P18065 |
| PAX8 | Q06710 |
| ARID1A | O14497 |
| OXT | P01178 |
| PAPPA | Q13219 |
| XRCC3 | O43542 |
| FOS | P01100 |
| COL1A2 | P08123 |
| ABCG5 | Q9H222 |
| CDK1 | P06493 |
| NANOS3 | P60323 |
| SETD2 | Q9BYW2 |
| CALB2 | P22676 |
| CALCA | P06881 |
| CD44 | P16070 |
| HGF | P14210 |
| GATA3 | P23771 |
| HNRNPA2B1 | P22626 |
| DMD | P11532 |
| DMC1 | Q14565 |
| WWOX | Q9NZC7 |
| VWF | P04275 |
| GPC3 | P51654 |
| VEGFC | P49767 |
| XIAP | P98170 |
| TPO | P07202 |
| ANGPT1 | Q15389 |
| LGALS3 | P17931 |
| HP | P00738 |
| SERPINA1 | P01009 |
| RELA | Q04206 |
| ANXA5 | P08758 |
| CYP1A1 | P04798 |
| MAP2K1 | Q02750 |
| CHEK1 | O14757 |
| BAG3 | O95817 |
| JAG1 | P78504 |
| RAF1 | P04049 |
| SOHLH2 | Q9NX45 |
| DCN | P07585 |
| SERPINA3 | P01011 |
| SLC2A1 | P11166 |
| TP73 | O15350 |
| LHX8 | Q68G74 |
| PPARA | Q07869 |
| GPER1 | Q99527 |
| LGR6 | Q9HBX8 |
| PRIM1 | P49642 |
| CFH | P08603 |
| EDNRA | P25101 |
| COL5A1 | P20908 |
| PGF | P49763 |
| C3 | P01024 |
| FBXW7 | Q969H0 |
| MAPK8 | P45983 |
| TNFSF11 | O14788 |
| MEN1 | O00255 |
| PIK3R1 | P27986 |
| FGF23 | Q9GZV9 |
| CSF1 | P09603 |
| PTGS1 | P23219 |
| TNFRSF1A | P19438 |
| SHH | Q15465 |
| EIF4ENIF1 | Q9NRA8 |
| ADAMTS19 | Q8TE59 |
| FGF7 | P21781 |
| SRC | P12931 |
| ABCA1 | O95477 |
| CCR6 | P51684 |
| MMP7 | P09237 |
| CTSD | P07339 |
| FLNA | P21333 |
| KRT20 | P35900 |
| PCNA | P12004 |
| HELLS | Q9NRZ9 |
| MKRN3 | Q13064 |
| LOX | P28300 |
| ACTB | P60709 |
| CST3 | P01034 |
| PON1 | P27169 |
| DSP | P15924 |
| INSL3 | P51460 |
| TGFA | P01135 |
| CGB5 | P0DN86 |
| TNXB | P22105 |
| CHAT | P28329 |
| NOTCH3 | Q9UM47 |
| EIF2B3 | Q9NR50 |
| FOXP2 | O15409 |
| FOLR1 | P15328 |
| RHOA | P61586 |
| FZD4 | Q9ULV1 |
| ELANE | P08246 |
| MYOD1 | P15172 |
| AGTR1 | P30556 |
| IL6R | P08887 |
| ITGB1 | P05556 |
| CEACAM5 | P06731 |
| POLG2 | Q9UHN1 |
| CDK12 | Q9NYV4 |
| SYCP2L | Q5T4T6 |
| NSF | P46459 |
| ALPP | P05187 |
| AGT | P01019 |
| TNNT2 | P45379 |
| MBL2 | P11226 |
| AURKA | O14965 |
| EIF2B1 | Q14232 |
| NPPB | P16860 |
| PECAM1 | P16284 |
| SLC34A2 | O95436 |
| TNFSF10 | P50591 |
| NBR1 | Q14596 |
| TMEM150B | A6NC51 |
| GHR | P10912 |
| DNMT3A | Q9Y6K1 |
| LPL | P06858 |
| TLR7 | Q9NYK1 |
| MME | P08473 |
| CDH2 | P19022 |
| MSH3 | P20585 |
| DNMT1 | P26358 |
| PCSK9 | Q8NBP7 |
| THBD | P07204 |
| HSPB1 | P04792 |
| JUP | P14923 |
| CLU | P10909 |
| PRKCD | Q05655 |
| IFI27 | P40305 |
| LEPR | P48357 |
| CASR | P41180 |
| RBBP8 | Q99708 |
| CCNB1 | P14635 |
| ACTA1 | P68133 |
| XRCC6 | P12956 |
| SP1 | P08047 |
| NPPA | P01160 |
| HSPD1 | P10809 |
| IFNA1 | P01562 |
| CLDN3 | O15551 |
| KISS1R | Q969F8 |
| XRCC1 | P18887 |
| CCNE1 | P24864 |
| MAPK3 | P27361 |
| E2F1 | Q01094 |
| CPEB1 | Q9BZB8 |
| SNAI2 | O43623 |
| PHB1 | P35232 |
| PTK2 | Q05397 |
| RAD52 | P43351 |
| TCF12 | Q99081 |
| TNNI3 | P19429 |
| NQO1 | P15559 |
| DIRAS3 | O95661 |
| NR3C1 | P04150 |
| FBLN5 | Q9UBX5 |
| ABCG2 | Q9UNQ0 |
| CD4 | P01730 |
| PRKAR1A | P10644 |
| ATP7B | P35670 |
| CD274 | Q9NZQ7 |
| TFRC | P02786 |
| CXCR4 | P61073 |
| GHRL | Q9UBU3 |
| HMOX1 | P09601 |
| NKX2-5 | P52952 |
| NKX2-1 | P43699 |
| RCBTB1 | Q8NDN9 |
| SMAD7 | O15105 |
| XPNPEP2 | O43895 |
| CCN2 | P29279 |
| PLAT | P00750 |
| EFEMP2 | O95967 |
| SPARC | P09486 |
| CYCS | P99999 |
| SMAD2 | Q15796 |
| BANF1 | O75531 |
| HELQ | Q8TDG4 |
| MYBPC3 | Q14896 |
| CD40 | P25942 |
| BCL2L11 | O43521 |
| TACSTD2 | P09758 |
| TP53BP1 | Q12888 |
| TXN | P10599 |
| TOP1 | P11387 |
| PRKDC | P78527 |
| SELP | P16109 |
| LTBP1 | Q14766 |
| INHBB | P09529 |
| DPP4 | P27487 |
| INHBA | P08476 |
| IL3 | P08700 |
| RMND1 | Q9NWS8 |
| SLPI | P03973 |
| ABL1 | P00519 |
| MIF | P14174 |
| TTR | P02766 |
| PLCG1 | P19174 |
| H2AX | P16104 |
| DMRT1 | Q9Y5R6 |
| ETS1 | P14921 |
| IL2RA | P01589 |
| FGF8 | P55075 |
| GAA | P10253 |
| ERCC8 | Q13216 |
| IL1RN | P18510 |
| HMGCR | P04035 |
| MCPH1 | Q8NEM0 |
| ACTA2 | P62736 |
| ABCC1 | P33527 |
| SALL4 | Q9UJQ4 |
| PIK3CG | P48736 |
| APEX1 | P27695 |
| EIF2AK3 | Q9NZJ5 |
| SNAI1 | O95863 |
| GPR3 | P46089 |
| NPM1 | P06748 |
| CFLAR | O15519 |
| PDGFB | P01127 |
| SST | P61278 |
| EPHB4 | P54760 |
| VCP | P55072 |
| GSTM1 | P09488 |
| SDHB | P21912 |
| HSPA1A | P0DMV8 |
| SELE | P16581 |
| CLDN4 | O14493 |
| RYR2 | Q92736 |
| PAEP | P09466 |
| ABCG8 | Q9H221 |
| HSPA4 | P34932 |
| NEK8 | Q86SG6 |
| CXCL1 | P09341 |
| ATRX | P46100 |
| IGFBP4 | P22692 |
| HLA-G | P17693 |
| FGF1 | P05230 |
| MECP2 | P51608 |
| NFKBIA | P25963 |
| KRT18 | P05783 |
| PLAUR | Q03405 |
| LCAT | P04180 |
| DKC1 | O60832 |
| GLI2 | P10070 |
| ROCK1 | Q13464 |
| VEGFD | O43915 |
| DNMT3B | Q9UBC3 |
| TBX1 | O43435 |
| GDF15 | Q99988 |
| CD36 | P16671 |
| HMGB1 | P09429 |
| PARP2 | Q9UGN5 |
| CASP1 | P29466 |
| DGCR8 | Q8WYQ5 |
| TFAP2A | P05549 |
| DIABLO | Q9NR28 |
| TIMP3 | P35625 |
| DACH2 | Q96NX9 |
| KAT6A | Q92794 |
| FBLN1 | P23142 |
| CDC25A | P30304 |
| GRN | P28799 |
| CD34 | P28906 |
| NRG1 | Q02297 |
| SAMD9 | Q5K651 |
| RPS6KB1 | P23443 |
| FANCI | Q9NVI1 |
| PROM1 | O43490 |
| SULF1 | Q8IWU6 |
| IL7 | P13232 |
| NLRP3 | Q96P20 |
| ABCC2 | Q92887 |
| HLA-DQB1 | P01920 |
| MAP2K2 | P36507 |
| PLK1 | P53350 |
| IRS1 | P35568 |
| VCAM1 | P19320 |
| CDKN3 | Q16667 |
| SPATA22 | Q8NHS9 |
| TWIST2 | Q8WVJ9 |
| NRP1 | O14786 |
| KLK7 | P49862 |
| ZP2 | Q05996 |
| MUC5AC | P98088 |
| TOP2A | P11388 |
| ZEB2 | O60315 |
| PRDM9 | Q9NQV7 |
| CASP9 | P55211 |
| SERPINB2 | P05120 |
| ITGA2 | P17301 |
| MED12 | Q93074 |
| LIF | P15018 |
| CD8A | P01732 |
| CCBE1 | Q6UXH8 |
| CCNA2 | P20248 |
| CEP290 | O15078 |
| CYP11B2 | P19099 |
| POLD1 | P28340 |
| GSK3B | P49841 |
| CYP1B1 | Q16678 |
| ATP7A | Q04656 |
| RBP4 | P02753 |
| MPO | P05164 |
| CD46 | P15529 |
| CCNG1 | P51959 |
| NCOA3 | Q9Y6Q9 |
| TXNRD2 | Q9NNW7 |
| DHFR | P00374 |
| AXIN1 | O15169 |
| HNF4A | P41235 |
| LRRC56 | Q8IYG6 |
| BSG | P35613 |
| SPO11 | Q9Y5K1 |
| IGSF10 | Q6WRI0 |
| KRT19 | P08727 |
| NCAM1 | P13591 |
| ABCB4 | P21439 |
| SMPD1 | P17405 |
| XRCC4 | Q13426 |
| HSD11B1 | P28845 |
| ITGB4 | P16144 |
| MSX2 | P35548 |
| FLT4 | P35916 |
| HSPA8 | P11142 |
| GATA6 | Q92908 |
| MAPK10 | P53779 |
| AKT3 | Q9Y243 |
| NOD2 | Q9HC29 |
| MUC4 | Q99102 |
| NGF | P01138 |
| EPRS1 | P07814 |
| KRT8 | P05787 |
| TNFRSF10B | O14763 |
| MTA1 | Q13330 |
| NPR2 | P20594 |
| EZH2 | Q15910 |
| ENPP1 | P22413 |
| HBEGF | Q99075 |
| PAX3 | P23760 |
| GSTT1 | P30711 |
| ANXA2 | P07355 |
| NRAS | P01111 |
| ITGAV | P06756 |
| CDK6 | Q00534 |
| TUBB | P07437 |
| PROP1 | O75360 |
| TYMP | P19971 |
| HMGA2 | P52926 |
| ZEB1 | P37275 |
| VTCN1 | Q7Z7D3 |
| SMC1B | Q8NDV3 |
| TF | P02787 |
| PRKCA | P17252 |
| HSP90AA1 | P07900 |
| NACC1 | Q96RE7 |
| PKHD1 | P08F94 |
| CSF1R | P07333 |
| SOX3 | P41225 |
| HDAC9 | Q9UKV0 |
| RAN | P62826 |
| CD40LG | P29965 |
| KARS1 | Q15046 |
| CDH5 | P33151 |
| CASP10 | Q92851 |
| ASNS | P08243 |
| ATG7 | O95352 |
| HBB | P68871 |
| ERBB4 | Q15303 |
| RASSF1 | Q9NS23 |
| RUNX1 | Q01196 |
| SKP2 | Q13309 |
| AREG | P15514 |
| IFT140 | Q96RY7 |
| SUFU | Q9UMX1 |
| LIPC | P11150 |
| L1CAM | P32004 |
| GADD45A | P24522 |
| SOX2 | P48431 |
| PCSK1 | P29120 |
| CLCNKB | P51801 |
| RTEL1 | Q9NZ71 |
| PRSS23 | O95084 |
| TTC21B | Q7Z4L5 |
| TNFRSF10A | O00220 |
| CRYAB | P02511 |
| NR3C2 | P08235 |
| GATA1 | P15976 |
| SYP | P08247 |
| YBX1 | P67809 |
| FXN | Q16595 |
| ALDH18A1 | P54886 |
| U2AF1 | Q01081 |
| RUNX2 | Q13950 |
| MMP13 | P45452 |
| SHC1 | P29353 |
| ABCB11 | O95342 |
| TGM2 | P21980 |
| NME1 | P15531 |
| JAK1 | P23458 |
| ICOSLG | O75144 |
| UIMC1 | Q96RL1 |
| PDCD1 | Q15116 |
| BECN1 | Q14457 |
| NOS2 | P35228 |
| RPS19 | P39019 |
| COL5A2 | P05997 |
| AMBP | P02760 |
| VTN | P04004 |
| MCL1 | Q07820 |
| CP | P00450 |
| DLEU1 | O43261 |
| CD80 | P33681 |
| MEGF8 | Q7Z7M0 |
| MT-ATP6 | P00846 |
| DYNC2H1 | Q8NCM8 |
| GRIN2B | Q13224 |
| C9orf72 | Q96LT7 |
| PTHLH | P12272 |
| SELL | P14151 |
| CKB | P12277 |
| WNT5A | P41221 |
| SQSTM1 | Q13501 |
| KCNQ1 | P51787 |
| BSND | Q8WZ55 |
| ANKRD31 | Q8N7Z5 |
| CDH13 | P55290 |
| EZR | P15311 |
| LAMA2 | P24043 |
| SLC6A4 | P31645 |
| ARID1B | Q8NFD5 |
| CYP27A1 | Q02318 |
| CYFIP1 | Q7L576 |
| CXCL10 | P02778 |
| SOX11 | P35716 |
| ATP6V0A2 | Q9Y487 |
| KLK5 | Q9Y337 |
| IFT172 | Q9UG01 |
| TKT | P29401 |
| CXADR | P78310 |
| NF2 | P35240 |
| PRKD1 | Q15139 |
| IL11RA | Q14626 |
| MYCN | P04198 |
| AARS1 | P49588 |
| NSD1 | Q96L73 |
| PHEX | P78562 |
| OGG1 | O15527 |
| ATXN10 | Q9UBB4 |
| HFE | Q30201 |
| TFAP2B | Q92481 |
| CGB3 | P0DN86 |
| IDO1 | P14902 |
| FASN | P49327 |
| RETN | Q9HD89 |
| F7 | P08709 |
| CDC42 | P60953 |
| DCAF17 | Q5H9S7 |
| TNFRSF11B | O00300 |
| ACACA | Q13085 |
| SMARCA2 | P51531 |
| PYCR1 | P32322 |
| F2R | P25116 |
| TCF20 | Q9UGU0 |
| HDAC1 | Q13547 |
| VCAN | P13611 |
| CCND3 | P30281 |
| SARS2 | Q9NP81 |
| CDC25C | P30307 |
| PTTG1 | O95997 |
| PLAGL1 | Q9UM63 |
| CTSB | P07858 |
| TMEM185A | Q8NFB2 |
| TLR2 | O60603 |
| GBA1 | P04062 |
| ELAVL1 | Q15717 |
| PKP2 | Q99959 |
| CHGA | P10645 |
| MYL2 | P10916 |
| HLA-DQA1 | P01909 |
| SELENBP1 | Q13228 |
| NRIP1 | P48552 |
| GREM1 | O60565 |
| ATP8B1 | O43520 |
| TSHB | P01222 |
| FGF3 | P11487 |
| IL5 | P05113 |
| IL11 | P20809 |
| MGMT | P16455 |
| DNA2 | P51530 |
| CD86 | P42081 |
| LIG4 | P49917 |
| RARB | P10826 |
| MBNL1 | Q9NR56 |
| RAC1 | P63000 |
| FOXP3 | Q9BZS1 |
| NLRP5 | P59047 |
| TGFBR3 | Q03167 |
| NTRK3 | Q16288 |
| CCK | P06307 |
| CYP24A1 | Q07973 |
| IGFBP5 | P24593 |
| NFE2L2 | Q16236 |
| CTSL | P07711 |
| PDX1 | P52945 |
| ITGAM | P11215 |
| TAC3 | Q9UHF0 |
| TRPS1 | Q9UHF7 |
| PCMT1 | P22061 |
| GLA | P06280 |
| MYH6 | P13533 |
| STN1 | Q9H668 |
| FHL1 | Q13642 |
| GJB2 | P29033 |
| DBH | P09172 |
| OSR2 | Q8N2R0 |
| CCL5 | P13501 |
| LPA | P08519 |
| SMC1A | Q14683 |
| ANK2 | Q01484 |
| GK | P32189 |
| IL13 | P35225 |
| IKBKG | Q9Y6K9 |
| TFAM | Q00059 |
| CETP | P11597 |
| NANOG | Q9H9S0 |
| FGFR4 | P22455 |
| DDX4 | Q9NQI0 |
| FOXE1 | O00358 |
| CASP7 | P55210 |
| IL24 | Q13007 |
| CYP27B1 | O15528 |
| STAT5A | P42229 |
| GRP | P07492 |
| LGALS1 | P09382 |
| PTH1R | Q03431 |
| JUND | P17535 |
| TFF1 | P04155 |
| GJA4 | P35212 |
| AGTR2 | P50052 |
| APLN | Q9ULZ1 |
| HSD17B3 | P37058 |
| COL7A1 | Q02388 |
| PCCA | P05165 |
| OFD1 | O75665 |
| KLK4 | Q9Y5K2 |
| PRORP | O15091 |
| PDGFD | Q9GZP0 |
| IL4R | P24394 |
| CD247 | P20963 |
| ACAN | P16112 |
| LDLRAP1 | Q5SW96 |
| ITGA6 | P23229 |
| KL | Q9UEF7 |
| FKBP14 | Q9NWM8 |
| NOG | Q13253 |
| SYCP3 | Q8IZU3 |
| SFN | P31947 |
| CTCF | P49711 |
| HSPA5 | P11021 |
| PCCB | P05166 |
| CDKN2C | P42773 |
| ESCO2 | Q56NI9 |
| SAT1 | P21673 |
| ENO2 | P09104 |
| SLC25A24 | Q6NUK1 |
| FGF13 | Q92913 |
| FXR2 | P51116 |
| SOCS3 | O14543 |
| FREM1 | Q5H8C1 |
| CUL1 | Q13616 |
| DHCR7 | Q9UBM7 |
| GATA2 | P23769 |
| CCR7 | P32248 |
| ANKLE2 | Q86XL3 |
| EMD | P50402 |
| DUSP1 | P28562 |
| HLA-B | P01889 |
| GAST | P01350 |
| G6PD | P11413 |
| GRM5 | P41594 |
| CAT | P04040 |
| EXOSC3 | Q9NQT5 |
| TNNC1 | P63316 |
| FOXO1 | Q12778 |
| KPNA2 | P52292 |
| CD59 | P13987 |
| ITGB2 | P05107 |
| DDB2 | Q92466 |
| MYD88 | Q99836 |
| ACTN4 | O43707 |
| TPM3 | P06753 |
| EBAG9 | O00559 |
| XRCC5 | P13010 |
| KLK3 | P07288 |
| APAF1 | O14727 |
| SHOX | O15266 |
| ALX4 | Q9H161 |
| CDR2 | Q01850 |
| GPC4 | O75487 |
| CREBBP | Q92793 |
| NPHP3 | Q7Z494 |
| TRAF3IP1 | Q8TDR0 |
| GCLC | P48506 |
| ERCC6L2 | Q5T890 |
| NGFR | P08138 |
| UGT1A1 | P22309 |
| CASP2 | P42575 |
| ADAMTS13 | Q76LX8 |
| CDH3 | P22223 |
| NPHP1 | O15259 |
| ZNF713 | Q8N859 |
| SLC31A1 | O15431 |
| ADNP | Q9H2P0 |
| RPA1 | P27694 |
| USP53 | Q70EK8 |
| RHOD | O00212 |
| RSF1 | Q96T23 |
| ADRB2 | P07550 |
| MLX | Q9UH92 |
| FURIN | P09958 |
| INVS | Q9Y283 |
| THPO | P40225 |
| CTAG1B | P78358 |
| HSD11B2 | P80365 |
| ESRRG | P62508 |
| PRTN3 | P24158 |
| MSN | P26038 |
| ABCA12 | Q86UK0 |
| LDHA | P00338 |
| ABCC3 | O15438 |
| RASGRP1 | O95267 |
| RLN2 | P04090 |
| COL4A5 | P29400 |
| IL6ST | P40189 |
| PAPSS2 | O95340 |
| NAMPT | P43490 |
| WRAP53 | Q9BUR4 |
| EIF4EBP1 | Q13541 |
| APOC3 | P02656 |
| EIF4E | P06730 |
| TPM1 | P09493 |
| EPHX1 | P07099 |
| NPHP4 | O75161 |
| ASPM | Q8IZT6 |
| AGER | Q15109 |
| COL11A1 | P12107 |
| E2F2 | Q14209 |
| PRSS1 | P07477 |
| RARA | P10276 |
| TLK1 | Q9UKI8 |
| GNAI2 | P04899 |
| ARNT | P27540 |
| GPT | P24298 |
| PLOD1 | Q02809 |
| SFTPA1 | Q8IWL2 |
| FGR | P09769 |
| SLX4 | Q8IY92 |
| SLC17A5 | Q9NRA2 |
| CREB1 | P16220 |
| MGP | P08493 |
| ITGA5 | P08648 |
| XK | P51811 |
| CYP7A1 | P22680 |
| INF2 | Q27J81 |
| ZFPM2 | Q8WW38 |
| RPL10 | P27635 |
| KLRK1 | P26718 |
| IRF1 | P10914 |
| RAB23 | Q9ULC3 |
| SERPINF1 | P36955 |
| CDK7 | P50613 |
| MKI67 | P46013 |
| GGT1 | P19440 |
| NDUFAF3 | Q9BU61 |
| TUBB8 | Q3ZCM7 |
| ZIC1 | Q15915 |
| MIR7-3HG | Q8N6C7 |
| DNAH1 | Q9P2D7 |
| CR2 | P20023 |
| NOTCH4 | Q99466 |
| EFNB1 | P98172 |
| XPO1 | O14980 |
| ASAH1 | Q13510 |
| DANCR | P0C864 |
| SNCG | O76070 |
| PAX6 | P26367 |
| TNFRSF10C | O14798 |
| TH | P07101 |
| MTHFD1 | P11586 |
| S100B | P04271 |
| IFT80 | Q9P2H3 |
| CBS | P35520 |
| MEIOB | Q8N635 |
| ACTC1 | P68032 |
| RBM28 | Q9NW13 |
| CCR2 | P41597 |
| EDNRB | P24530 |
| PAK1 | Q13153 |
| CCL11 | P51671 |
| APP | P05067 |
| HAS2 | Q92819 |
| RPS20 | P60866 |
| UPF1 | Q92900 |
| TERF2 | Q15554 |
| IDH1 | O75874 |
| E2F4 | Q16254 |
| TUBA1B | P68363 |
| CFI | P05156 |
| PIK3CB | P42338 |
| SYCP1 | Q15431 |
| NR1H2 | P55055 |
| UMOD | P07911 |
| FEN1 | P39748 |
| DCK | P27707 |
| PTRH2 | Q9Y3E5 |
| CAPN10 | Q9HC96 |
| SATB2 | Q9UPW6 |
| ATF3 | P18847 |
| TOPBP1 | Q92547 |
| THY1 | P04216 |
| BMAL1 | O00327 |
| AQP2 | P41181 |
| SEPTIN9 | Q9UHD8 |
| ADM | P35318 |
| BBS9 | Q3SYG4 |
| MSX1 | P28360 |
| NEB | P20929 |
| IL21 | Q9HBE4 |
| EIF2S2 | P20042 |
| GRPR | P30550 |
| IL15 | P40933 |
| SNRPN | P63162 |
| CA9 | Q16790 |
| SDHA | P31040 |
| PXN | P49023 |
| MAPT | P10636 |
| FDX1 | P10109 |
| RPE65 | Q16518 |
| DSG2 | Q14126 |
| NANOS1 | Q8WY41 |
| SMARCAL1 | Q9NZC9 |
| NTRK2 | Q16620 |
| KRT14 | P02533 |
| CD19 | P15391 |
| DLK1 | P80370 |
| PALLD | Q8WX93 |
| SOX10 | P56693 |
| RPL5 | P46777 |
| CEACAM6 | P40199 |
| SYNE1 | Q8NF91 |
| CLCNKA | P51800 |
| KMT2D | O14686 |
| SPRY2 | O43597 |
| POSTN | Q15063 |
| NPY | P01303 |
| LTA | P01374 |
| PI3 | P19957 |
| ITGB6 | P18564 |
| KEAP1 | Q14145 |
| LYVE1 | Q9Y5Y7 |
| CLDN16 | Q9Y5I7 |
| CLDN14 | O95500 |
| NUP155 | O75694 |
| MYLK | Q15746 |
| SLC12A2 | P55011 |
| NIPBL | Q6KC79 |
| RIN2 | Q8WYP3 |
| MUC2 | Q02817 |
| LIG1 | P18858 |
| ACVR2B | Q13705 |
| MTR | Q99707 |
| FOXK2 | Q01167 |
| HSPG2 | P98160 |
| STX1A | Q16623 |
| ACKR3 | P25106 |
| SDHD | O14521 |
| CROCC | Q5TZA2 |
| FDXR | P22570 |
| SRD5A1 | P18405 |
| HTR1A | P08908 |
| SNCA | P37840 |
| LIG3 | P49916 |
| MUS81 | Q96NY9 |
| SDCCAG8 | Q86SQ7 |
| POLR2C | P19387 |
| ALDH2 | P05091 |
| COL11A2 | P13942 |
| GC | P02774 |
| NTHL1 | P78549 |
| MT-CO1 | P00395 |
| TNFRSF1B | P20333 |
| FGA | P02671 |
| CCR5 | P51681 |
| NAA10 | P41227 |
| YARS1 | P54577 |
| TGIF1 | Q15583 |
| SCGB1A1 | P11684 |
| BAP1 | Q92560 |
| IHH | Q14623 |
| AIFM1 | O95831 |
| ACP5 | P13686 |
| DAG1 | Q14118 |
| AGK | Q53H12 |
| GANAB | Q14697 |
| ACHE | P22303 |
| CACNA1S | Q13698 |
| IL2RB | P14784 |
| CRYAA | P02489 |
| SCNN1A | P37088 |
| CEP164 | Q9UPV0 |
| SERPINA7 | P05543 |
| DIO3 | P55073 |
| RSPO1 | Q2MKA7 |
| REL | Q04864 |
| YBX2 | Q9Y2T7 |
| HSPB2 | Q16082 |
| NR1H4 | Q96RI1 |
| LHB | P01229 |
| KASH5 | Q8N6L0 |
| RPS24 | P62847 |
| HERC1 | Q15751 |
| SERPINA6 | P08185 |
| ARAF | P10398 |
| SEMA3A | Q14563 |
| IFNB1 | P01574 |
| NPPC | P23582 |
| PHOX2B | Q99453 |
| PDE11A | Q9HCR9 |
| CDC73 | Q6P1J9 |
| CTC1 | Q2NKJ3 |
| CEBPB | P17676 |
| RHO | P08100 |
| NUDT10 | Q8NFP7 |
| CD47 | Q08722 |
| LTBP4 | Q8N2S1 |
| TNFAIP3 | P21580 |
| ABCC8 | Q09428 |
| ADA | P00813 |
| NDN | Q99608 |
| OXTR | P30559 |
| ROMO1 | P60602 |
| NPHS2 | Q9NP85 |
| COL4A1 | P02462 |
| DNAH8 | Q96JB1 |
| BMI1 | P35226 |
| VEGFB | P49765 |
| SCT | P09683 |
| LTF | P02788 |
| BRCC3 | P46736 |
| FYN | P06241 |
| HIRA | P54198 |
| SMARCA5 | O60264 |
| IQCB1 | Q15051 |
| CYB5A | P00167 |
| LMX1B | O60663 |
| DNAJC30 | Q96LL9 |
| FGF10 | O15520 |
| TMEM43 | Q9BTV4 |
| BRD4 | O60885 |
| C1S | P09871 |
| NLRP11 | P59045 |
| SLC25A13 | Q9UJS0 |
| FOXE3 | Q13461 |
| ACSL6 | Q9UKU0 |
| TARDBP | Q13148 |
| NCF1 | P14598 |
| SFTPD | P35247 |
| SMN1 | Q16637 |
| FOXC2 | Q99958 |
| HIC1 | Q14526 |
| TJP1 | Q07157 |
| AIP | O00170 |
| PITX2 | Q99697 |
| TNNI3K | Q59H18 |
| CYP26B1 | Q9NR63 |
| YARS2 | Q9Y2Z4 |
| NDUFAF2 | Q8N183 |
| PLA2G6 | O60733 |
| SPECC1L | Q69YQ0 |
| CYP1A2 | P05177 |
| GSN | P06396 |
| MLANA | Q16655 |
| TGM1 | P22735 |
| BCAP31 | P51572 |
| PSEN1 | P49768 |
| LIMK1 | P53667 |
| KRT5 | P13647 |
| TRAF6 | Q9Y4K3 |
| GHRH | P01286 |
| IL1R1 | P14778 |
| IDH2 | P48735 |
| MAGEL2 | Q9UJ55 |
| SLC52A2 | Q9HAB3 |
| YAP1 | P46937 |
| PTX3 | P26022 |
| BGN | P21810 |
| TNFRSF13B | O14836 |
| CYP2C9 | P11712 |
| HLA-A | P04439 |
| GATA5 | Q9BWX5 |
| PSMB8 | P28062 |
| MB | P02144 |
| SMO | Q99835 |
| ZFHX4 | Q86UP3 |
| DGKE | P52429 |
| VARS1 | P26640 |
| SRPRA | P08240 |
| KCNH2 | Q12809 |
| CD14 | P08571 |
| HTR2A | P28223 |
| IRS2 | Q9Y4H2 |
| BRSK1 | Q8TDC3 |
| OTX2 | P32243 |
| MKS1 | Q9NXB0 |
| C4A | P0C0L4 |
| IL7R | P16871 |
| SOS1 | Q07889 |
| CAMSAP2 | Q08AD1 |
| SOCS1 | O15524 |
| SKI | P12755 |
| SMN2 | Q16637 |
| SGO1 | Q5FBB7 |
| MDC1 | Q14676 |
| SLC25A3 | Q00325 |
| UBR4 | Q5T4S7 |
| SFRP4 | Q6FHJ7 |
| AURKB | Q96GD4 |
| SIGLEC5 | O15389 |
| NXF5 | Q9H1B4 |
| HAX1 | O00165 |
| OAS1 | P00973 |
| WDR62 | O43379 |
| B4GALNT1 | Q00973 |
| NUP133 | Q8WUM0 |
| SYCP2 | Q9BX26 |
| LDB3 | O75112 |
| LBR | Q14739 |
| MTRR | Q9UBK8 |
| ITGAL | P20701 |
| CISH | Q9NSE2 |
| FOXM1 | Q08050 |
| IL23R | Q5VWK5 |
| KDM6A | O15550 |
| NES | P48681 |
| HADHA | P40939 |
| SDC1 | P18827 |
| BSCL2 | Q96G97 |
| DDI1 | Q8WTU0 |
| DRD2 | P14416 |
| CHRM2 | P08172 |
| KCNN4 | O15554 |
| COL4A4 | P53420 |
| MTM1 | Q13496 |
| SOX8 | P57073 |
| ALMS1 | Q8TCU4 |
| GSR | P00390 |
| ABCC6 | O95255 |
| SLC2A4 | P14672 |
| RPS10 | P46783 |
| TPM2 | P07951 |
| IL33 | O95760 |
| BCKDHB | P21953 |
| DDC | P20711 |
| MT-ATP8 | P03928 |
| FANCB | Q8NB91 |
| ANAPC1 | Q9H1A4 |
| APOA4 | P06727 |
| IGFBP6 | P24592 |
| CRKL | P46109 |
| BTK | Q06187 |
| TEX11 | Q8IYF3 |
| PLP1 | P60201 |
| APOH | P02749 |
| ROBO4 | Q8WZ75 |
| LIFR | P42702 |
| CYP3A5 | P20815 |
| SAA1 | P0DJI8 |
| KNG1 | P01042 |
| SNRPA | P09012 |
| RHOF | Q9HBH0 |
| EDN3 | P14138 |
| TSPAN12 | O95859 |
| CCL3 | P10147 |
| HELB | Q8NG08 |
| HS6ST1 | O60243 |
| STAG1 | Q8WVM7 |
| AMACR | Q9UHK6 |
| NOS1 | P29475 |
| LEMD3 | Q9Y2U8 |
| YY1 | P25490 |
| GFAP | P14136 |
| MS4A1 | P11836 |
| UBB | P0CG47 |
| FGF4 | P08620 |
| CSTB | P04080 |
| AKR1B1 | P15121 |
| IFNA2 | P01563 |
| DYRK1A | Q13627 |
| CDC7 | O00311 |
| F13A1 | P00488 |
| RNASE3 | P12724 |
| SCARB1 | Q8WTV0 |
| F10 | P00742 |
| TMCO1 | Q9UM00 |
| MAGED2 | Q9UNF1 |
| EHMT1 | Q9H9B1 |
| BAZ1B | Q9UIG0 |
| CXCR2 | P25025 |
| CD28 | P10747 |
| COL25A1 | Q9BXS0 |
| HBG2 | P69892 |
| HDAC2 | Q92769 |
| BBS10 | Q8TAM1 |
| IFT122 | Q9HBG6 |
| FARP1 | Q9Y4F1 |
| RRAGA | Q7L523 |
| NFKBIZ | Q9BYH8 |
| DEFB1 | P60022 |
| POGZ | Q7Z3K3 |
| PTPN3 | P26045 |
| GARS1 | P41250 |
| RPL11 | P62913 |
| CYP2E1 | P05181 |
| FGF9 | P31371 |
| STRA8 | Q7Z7C7 |
| EXOSC9 | Q06265 |
| BTD | P43251 |
| DNM2 | P50570 |
| UGCG | Q16739 |
| CYP2C19 | P33261 |
| HNRNPA1 | P09651 |
| HTR2C | P28335 |
| POLB | P06746 |
| NPHS1 | O60500 |
| ALDH3A2 | P51648 |
| MLXIPL | Q9NP71 |
| DSC2 | Q02487 |
| SPATA16 | Q9BXB7 |
| DKK1 | O94907 |
| SLC25A20 | O43772 |
| GTF2H5 | Q6ZYL4 |
| TLR3 | O15455 |
| FRAS1 | Q86XX4 |
| HARS1 | P12081 |
| DMPK | Q09013 |
| DHH | O43323 |
| ADAMTS5 | Q9UNA0 |
| NEU1 | Q99519 |
| TNFRSF11A | Q9Y6Q6 |
| TYR | P14679 |
| TMEM67 | Q5HYA8 |
| RPS26 | P62854 |
| TNFSF12 | O43508 |
| S100A8 | P05109 |
| USP7 | Q93009 |
| PIGA | P37287 |
| DNAJB11 | Q9UBS4 |
| SREBF2 | Q12772 |
| ALOX5 | P09917 |
| RFPL4A | A6NLU0 |
| PPIG | Q13427 |
| CBX2 | Q14781 |
| HTRA1 | Q92743 |
| HOXA9 | P31269 |
| SLC22A18 | Q96BI1 |
| SETX | Q7Z333 |
| KCNJ11 | Q14654 |
| HPRT1 | P00492 |
| PEX13 | Q92968 |
| CLCN1 | P35523 |
| FCGR2A | P12318 |
| TOR1A | O14656 |
| UFSP2 | Q9NUQ7 |
| DPYD | Q12882 |
| LAMC1 | P11047 |
| TRIM37 | O94972 |
| TBK1 | Q9UHD2 |
| NAT2 | P11245 |
| KMT2A | Q03164 |
| PNPO | Q9NVS9 |
| PEX19 | P40855 |
| TET2 | Q6N021 |
| CD2AP | Q9Y5K6 |
| CC2D2A | Q9P2K1 |
| OTC | P00480 |
| VIP | P01282 |
| DLL1 | O00548 |
| SPTAN1 | Q13813 |
| DLL4 | Q9NR61 |
| LMNB2 | Q03252 |
| WDR11 | Q9BZH6 |
| TOP1MT | Q969P6 |
| MCM3AP | O60318 |
| NR1I2 | O75469 |
| COL4A3 | Q01955 |
| GHSR | Q92847 |
| TERF1 | P54274 |
| SOCS2 | O14508 |
| RAD9A | Q99638 |
| TLR9 | Q9NR96 |
| PEX26 | Q7Z412 |
| RPS17 | P08708 |
| GDF2 | Q9UK05 |
| ZIC2 | O95409 |
| GCK | P35557 |
| PLK4 | O00444 |
| DDB1 | Q16531 |
| IGHE | P01854 |
| TAP2 | Q03519 |
| BCL7B | Q9BQE9 |
| HTRA2 | O43464 |
| HADHB | P55084 |
| MFN2 | O95140 |
| XPO5 | Q9HAV4 |
| TCF7L2 | Q9NQB0 |
| ACADM | P11310 |
| ALG2 | Q9H553 |
| PARD3 | Q8TEW0 |
| LMNB1 | P20700 |
| MYH9 | P35579 |
| MC4R | P32245 |
| RPS27 | P42677 |
| MAF | O75444 |
| TPH1 | P17752 |
| SMC3 | Q9UQE7 |
| NUP160 | Q12769 |
| CAMP | P49913 |
| STAT4 | Q14765 |
| RUNX3 | Q13761 |
| PIK3C2A | O00443 |
| BEX3 | Q00994 |
| MACF1 | O94854 |
| VEZF1 | Q14119 |
| CRHR1 | P34998 |
| TAMM41 | Q96BW9 |
| EPOR | P19235 |
| JAZF1 | Q86VZ6 |
| IFT52 | Q9Y366 |
| FUS | P35637 |
| EPAS1 | Q99814 |
| PDIA4 | P13667 |
| NUP93 | Q8N1F7 |
| SPAST | Q9UBP0 |
| MBP | P02686 |
| SULT1A1 | P50225 |
| PAX7 | P23759 |
| MAD2L2 | Q9UI95 |
| EPPIN | O95925 |
| NR2C2 | P49116 |
| COL6A3 | P12111 |
| AVP | P01185 |
| ADAMTS4 | O75173 |
| APC2 | O95996 |
| PADI6 | Q6TGC4 |
| CRHBP | P24387 |
| DSG1 | Q02413 |
| TRH | P20396 |
| PDGFRL | Q15198 |
| PEX2 | P28328 |
| ACVR2A | P27037 |
| F2RL1 | P55085 |
| MEF2A | Q02078 |
| SLC37A4 | O43826 |
| PBX1 | P40424 |
| SLC22A5 | O76082 |
| CASC2 | Q8IU53 |
| DHX9 | Q08211 |
| GTF2I | P78347 |
| FCN2 | Q15485 |
| RAD21 | O60216 |
| CEL | P19835 |
| PROKR2 | Q8NFJ6 |
| LRP1 | Q07954 |
| SCNN1B | P51168 |
| UFD1 | Q92890 |
| UQCRFS1 | P47985 |
| DCTN1 | Q14203 |
| ALOXE3 | Q9BYJ1 |
| GZMB | P10144 |
| MT-ND2 | P03891 |
| MMP12 | P39900 |
| S1PR1 | P21453 |
| BRD3 | Q15059 |
| PPARGC1A | Q9UBK2 |
| PLEC | Q15149 |
| RLN1 | P04808 |
| UNG | P13051 |
| DNASE1 | P24855 |
| TBX4 | P57082 |
| S100A9 | P06702 |
| NOD1 | Q9Y239 |
| RNF168 | Q8IYW5 |
| CPT2 | P23786 |
| PTPRC | P08575 |
| ACE2 | Q9BYF1 |
| PRDM1 | O75626 |
| CHKA | P35790 |
| FLG | P20930 |
| RPL35A | P18077 |
| TAP1 | Q03518 |
| CCNT2 | O60583 |
| TUBB3 | Q13509 |
| MYO5A | Q9Y4I1 |
| IL22 | Q9GZX6 |
| ACVR1 | Q04771 |
| SFTPA2 | Q8IWL1 |
| FBN2 | P35556 |
| CARS1 | P49589 |
| CNTNAP1 | P78357 |
| KDM1A | O60341 |
| RUVBL1 | Q9Y265 |
| MAD2L1 | Q13257 |
| IKBKB | O14920 |
| IL12RB1 | P42701 |
| THOC6 | Q86W42 |
| DDX3X | O00571 |
| FKBP6 | O75344 |
| PRKACA | P17612 |
| F8 | P00451 |
| IRS4 | O14654 |
| CD79A | P11912 |
| MTAP | Q13126 |
| BCR | P11274 |
| UBE2D1 | P51668 |
| TBCE | Q15813 |
| ALOX12B | O75342 |
| NPC1 | O15118 |
| DYSF | O75923 |
| NFIX | Q14938 |
| ASPH | Q12797 |
| KCNJ2 | P63252 |
| PTGER2 | P43116 |
| GLB1 | P16278 |
| CLCN7 | P51798 |
| GCG | P01275 |
| UFL1 | O94874 |
| KCNE1 | P15382 |
| EGR1 | P18146 |
| ACADS | P16219 |
| GHRHR | Q02643 |
| UBA1 | P22314 |
| FTO | Q9C0B1 |
| SALL1 | Q9NSC2 |
| DMP1 | Q13316 |
| NODAL | Q96S42 |
| SLC6A3 | Q01959 |
| ACTG1 | P63261 |
| PEX1 | O43933 |
| CDC20 | Q12834 |
| HACD1 | B0YJ81 |
| TARS1 | P26639 |
| SDHC | Q99643 |
| PROS1 | P07225 |
| CENPE | Q02224 |
| GALE | Q14376 |
| TACR3 | P29371 |
| GPX3 | P22352 |
| SLC11A1 | P49279 |
| TAB2 | Q9NYJ8 |
| ATP6V1B1 | P15313 |
| MMACHC | Q9Y4U1 |
| KCNJ5 | P48544 |
| CPS1 | P31327 |
| PAFAH1B1 | P43034 |
| FIG4 | Q92562 |
| COL6A1 | P12109 |
| COL18A1 | P39060 |
| ATG9A | Q7Z3C6 |
| CALB1 | P05937 |
| ALG8 | Q9BVK2 |
| NHERF1 | O14745 |
| ABCA4 | P78363 |
| P4HB | P07237 |
| RFC2 | P35250 |
| GATM | P50440 |
| AHR | P35869 |
| HUWE1 | Q7Z6Z7 |
| ADA2 | Q9NZK5 |
| REST | Q13127 |
| TRPC6 | Q9Y210 |
| TTC7A | Q9ULT0 |
| RNF8 | O76064 |
| LZTR1 | Q8N653 |
| COG2 | Q14746 |
| PKM | P14618 |
| SYNE2 | Q8WXH0 |
| MSTN | O14793 |
| HESX1 | Q9UBX0 |
| TNFRSF8 | P28908 |
| SLC13A5 | Q86YT5 |
| TRIP11 | Q15643 |
| SLC25A4 | P12235 |
| BUD23 | O43709 |
| RPS27A | P62979 |
| CYP2B6 | P20813 |
| COX5A | P20674 |
| ATRIP | Q8WXE1 |
| RMI2 | Q96E14 |
| UGT1A6 | P19224 |
| ZP4 | Q12836 |
| BUB1 | O43683 |
| RPGRIP1L | Q68CZ1 |
| GUSB | P08236 |
| AGPAT2 | O15120 |
| IL1RAPL1 | Q9NZN1 |
| CD81 | P60033 |
| CXCL9 | Q07325 |
| UPF3B | Q9BZI7 |
| CHI3L1 | P36222 |
| COG4 | Q9H9E3 |
| KIF7 | Q2M1P5 |
| CCN1 | O00622 |
| PDCD4 | Q53EL6 |
| CXCR3 | P49682 |
| ACADVL | P49748 |
| CD55 | P08174 |
| ASS1 | P00966 |
| CALR | P27797 |
| CHD4 | Q14839 |
| BBS1 | Q8NFJ9 |
| THBS4 | P35443 |
| RPL15 | P61313 |
| DMBT1 | Q9UGM3 |
| MASP2 | O00187 |
| WHAMM | Q8TF30 |
| ATG2A | Q2TAZ0 |
| MT-ND1 | P03886 |
| TFEB | P19484 |
| USH2A | O75445 |
| LONP1 | P36776 |
| BMP7 | P18075 |
| SYNGAP1 | Q96PV0 |
| STS | P08842 |
| ATP2A2 | P16615 |
| LAMP2 | P13473 |
| CIITA | P33076 |
| GLS | O94925 |
| WNT10A | Q9GZT5 |
| BCL6 | P41182 |
| POLR3A | O14802 |
| FGFRL1 | Q8N441 |
| TTI2 | Q6NXR4 |
| APTX | Q7Z2E3 |
| PDGFA | P04085 |
| ZPR1 | O75312 |
| CCL18 | P55774 |
| S100A1 | P23297 |
| GALM | Q96C23 |
| PEG3 | Q9GZU2 |
| RAG1 | P15918 |
| SLC12A4 | Q9UP95 |
| POLR2A | P24928 |
| CHRND | Q07001 |
| MYO1E | Q12965 |
| CSF3R | Q99062 |
| FAT4 | Q6V0I7 |
| GFRA1 | P56159 |
| LATS1 | O95835 |
| RPS4X | P62701 |
| GAS1 | P54826 |
| MOV10 | Q9HCE1 |
| SMARCD2 | Q92925 |
| TNC | P24821 |
| UBR2 | Q8IWV8 |
| TRPM7 | Q96QT4 |
| PGM1 | P36871 |
| PSEN2 | P49810 |
| TRAF2 | Q12933 |
| MCM7 | P33993 |
| CCR3 | P51677 |
| DDR2 | Q16832 |
| LRBA | P50851 |
| CYLD | Q9NQC7 |
| ITGAX | P20702 |
| RBPJ | Q06330 |
| GET3 | O43681 |
| CTNND1 | O60716 |
| HLA-C | P10321 |
| SF3B1 | O75533 |
| TRIP13 | Q15645 |
| IL1RAPL2 | Q9NP60 |
| LRPPRC | P42704 |
| IBSP | P21815 |
| DARS2 | Q6PI48 |
| GJB1 | P08034 |
| CYP2D6 | P10635 |
| CRIPTO | P13385 |
| MEST | Q5EB52 |
| CD69 | Q07108 |
| CDC45 | O75419 |
| SMAD1 | Q15797 |
| EIF6 | P56537 |
| GTF2IRD1 | Q9UHL9 |
| SSH1 | Q8WYL5 |
| SLC2A2 | P11168 |
| ANG | P03950 |
| MATR3 | P43243 |
| FLAD1 | Q8NFF5 |
| IFIH1 | Q9BYX4 |
| LINC01554 | Q52M75 |
| ARF1 | P84077 |
| CHRNA3 | P32297 |
| LFNG | Q8NES3 |
| OTUD5 | Q96G74 |
| RNF213 | Q63HN8 |
| ACVRL1 | P37023 |
| STAT2 | P52630 |
| HMGA1 | P17096 |
| TTC16 | Q8NEE8 |
| LOXL1 | Q08397 |
| SLC16A1 | P53985 |
| PF4 | P02776 |
| GNB1 | P62873 |
| H6PD | O95479 |
| GRB2 | P62993 |
| SIX2 | Q9NPC8 |
| SLC25A1 | P53007 |
| PRKAG2 | Q9UGJ0 |
| PRODH | O43272 |
| ABCG1 | P45844 |
| ADAM17 | P78536 |
| TUBA1A | Q71U36 |
| PAH | P00439 |
| CHRNG | P07510 |
| C4B | P0C0L5 |
| SH2B3 | Q9UQQ2 |
| ETFDH | Q16134 |
| SLC5A5 | Q92911 |
| CDH23 | Q9H251 |
| ALDH1A2 | O94788 |
| AOC1 | P19801 |
| CXCR1 | P25024 |
| CSH1 | P0DML2 |
| TBL2 | Q9Y4P3 |
| FOXJ1 | Q92949 |
| ADD1 | P35611 |
| NTS | P30990 |
| PIBF1 | Q8WXW3 |
| TMEM231 | Q9H6L2 |
| PRKG1 | Q13976 |
| RARS2 | Q5T160 |
| NCOR1 | O75376 |
| DCLRE1B | Q9H816 |
| PRSS2 | P07478 |
| MBD4 | O95243 |
| ATXN8OS | P0DMR3 |
| CENPI | Q92674 |
| PNPLA7 | Q6ZV29 |
| SLC19A1 | P41440 |
| F9 | P00740 |
| HBA2 | P69905 |
| ZIC4 | Q8N9L1 |
| HOXA10 | P31260 |
| ANTXR1 | Q9H6X2 |
| SUN1 | O94901 |
| ATAD1 | Q8NBU5 |
| CDKL5 | O76039 |
| TSPY1 | Q01534 |
| MADD | Q8WXG6 |
| NDUFB11 | Q9NX14 |
| PYY | P10082 |
| THBS2 | P35442 |
| EWSR1 | Q01844 |
| FAM111A | Q96PZ2 |
| ARG1 | P05089 |
| EDARADD | Q8WWZ3 |
| ITGA3 | P26006 |
| WNT1 | P04628 |
| DHRS11 | Q6UWP2 |
| HCRT | O43612 |
| TCTN3 | Q6NUS6 |
| CLDN5 | O00501 |
| CLIP2 | Q9UDT6 |
| PRF1 | P14222 |
| SGCD | Q92629 |
| FABP4 | P15090 |
| DDIT3 | P35638 |
| EARS2 | Q5JPH6 |
| POLR2L | P62875 |
| CDC6 | Q99741 |
| LNPEP | Q9UIQ6 |
| MID1 | O15344 |
| ROBO2 | Q9HCK4 |
| ANAPC7 | Q9UJX3 |
| SWSAP1 | Q6NVH7 |
| UBR5 | O95071 |
| HNRNPK | P61978 |
| FLI1 | Q01543 |
| RLIM | Q9NVW2 |
| COQ8B | Q96D53 |
| GNA11 | P29992 |
| SIX3 | O95343 |
| MMP11 | P24347 |
| TBX21 | Q9UL17 |
| IL12B | P29460 |
| SOX14 | O95416 |
| CITED2 | Q99967 |
| KDM4C | Q9H3R0 |
| TMEM216 | Q9P0N5 |
| MPLKIP | Q8TAP9 |
| ABCB7 | O75027 |
| JAK3 | P52333 |
| SLC26A4 | O43511 |
| WFS1 | O76024 |
| AQP1 | P29972 |
| HPS3 | Q969F9 |
| PTGIS | Q16647 |
| RPL26 | P61254 |
| FADD | Q13158 |
| SIM1 | P81133 |
| FGF18 | O76093 |
| PSMA7 | O14818 |
| HCK | P08631 |
| RMI1 | Q9H9A7 |
| LAMP1 | P11279 |
| LCK | P06239 |
| CFL1 | P23528 |
| EIF4H | Q15056 |
| GTF2IRD2 | Q86UP8 |
| METTL27 | Q8N6F8 |
| VPS37D | Q86XT2 |
| TMEM270 | Q6UE05 |
| CYBA | P13498 |
| OCLN | Q16625 |
| PPIB | P23284 |
| ABCB6 | Q9NP58 |
| YWHAG | P61981 |
| ALG9 | Q9H6U8 |
| NSD2 | O96028 |
| SRSF6 | Q13247 |
| FA2H | Q7L5A8 |
| NUP85 | Q9BW27 |
| FHL2 | Q14192 |
| PFAS | O15067 |
| RIGI | O95786 |
| BMP8B | P34820 |
| GAPDHS | O14556 |
| ALPG | P10696 |
| MT-ND5 | P03915 |
| SUZ12 | Q15022 |
| OPTN | Q96CV9 |
| KRT10 | P13645 |
| ADAM10 | O14672 |
| HES7 | Q9BYE0 |
| PUM1 | Q14671 |
| IFNGR1 | P15260 |
| TSEN54 | Q7Z6J9 |
| FCN3 | O75636 |
| LRRK2 | Q5S007 |
| MT-ND3 | P03897 |
| RASA1 | P20936 |
| PABPC1 | P11940 |
| IL10RA | Q13651 |
| VCL | P18206 |
| DPP9 | Q86TI2 |
| CEBPA | P49715 |
| EREG | O14944 |
| CSPP1 | Q1MSJ5 |
| CYP51A1 | Q16850 |
| ATXN2 | Q99700 |
| COL9A2 | Q14055 |
| RPS7 | P62081 |
| DNM1L | O00429 |
| RIF1 | Q5UIP0 |
| CDH11 | P55287 |
| CSN1S1 | P47710 |
| RAPSN | Q13702 |
| PEX14 | O75381 |
| LGALS7 | P47929 |
| SCP2 | P22307 |
| UBC | P0CG48 |
| RARS1 | P54136 |
| CTCFL | Q8NI51 |
| MYOG | P15173 |
| BAG5 | Q9UL15 |
| STUB1 | Q9UNE7 |
| PSG2 | P11465 |
| PGK1 | P00558 |
| RBM5 | P52756 |
| PRPF31 | Q8WWY3 |
| CACNA1C | Q13936 |
| AP3D1 | O14617 |
| PRDM16 | Q9HAZ2 |
| MAGT1 | Q9H0U3 |
| CORIN | Q9Y5Q5 |
| SEM1 | P60896 |
| RPA2 | P15927 |
| GPX4 | P36969 |
| SPG7 | Q9UQ90 |
| SFTA3 | P0C7M3 |
| SHMT1 | P34896 |
| ANLN | Q9NQW6 |
| ANPEP | P15144 |
| BLOC1S1 | P78537 |
| PAX5 | Q02548 |
| BHLHE40 | O14503 |
| HOXB7 | P09629 |
| HERC2 | O95714 |
| AP3B1 | O00203 |
| F12 | P00748 |
| KANSL1 | Q7Z3B3 |
| CHUK | O15111 |
| NSUN2 | Q08J23 |
| PRKCE | Q02156 |
| STAT5B | P51692 |
| CPB2 | Q96IY4 |
| COL6A2 | P12110 |
| COX10 | Q12887 |
| ALG12 | Q9BV10 |
| PIK3CD | O00329 |
| PON2 | Q15165 |
| TAFAZZIN | Q16635 |
| AIM2 | O14862 |
| XPNPEP3 | Q9NQH7 |
| FLT3 | P36888 |
| MFAP5 | Q13361 |
| WNT9B | O14905 |
| CHP1 | Q99653 |
| NCL | P19338 |
| TLR5 | O60602 |
| MT-RNR1 | A0A0C5B5G6 |
| FKBP10 | Q96AY3 |
| LBP | P18428 |
| SLC33A1 | O00400 |
| SCD | O00767 |
| KRT13 | P13646 |
| STOX1 | Q6ZVD7 |
| SRSF2 | Q01130 |
| GCLM | P48507 |
| NDRG1 | Q92597 |
| MYH10 | P35580 |
| GDNF | P39905 |
| SOST | Q9BQB4 |
| TCF4 | P15884 |
| SOX6 | P35712 |
| SF3B4 | Q15427 |
| GNPTG | Q9UJJ9 |
| SPG11 | Q96JI7 |
| UBE3A | Q05086 |
| LAMC2 | Q13753 |
| ROR2 | Q01974 |
| TRAP1 | Q12931 |
| NAGLU | P54802 |
| CNTLN | Q9NXG0 |
| SMARCA1 | P28370 |
| POU1F1 | P28069 |
| KIF11 | P52732 |
| SIL1 | Q9H173 |
| CHD8 | Q9HCK8 |
| RAB27A | P51159 |
| EYA4 | O95677 |
| SIX5 | Q8N196 |
| CYP2C8 | P10632 |
| PPARD | Q03181 |
| RPS16 | P62249 |
| FOLR3 | P41439 |
| IGF2BP3 | O00425 |
| GMNN | O75496 |
| GK2 | Q14410 |
| NPY2R | P49146 |
| MACROD1 | Q9BQ69 |
| MICA | Q29983 |
| LIPE | Q05469 |
| FTH1 | P02794 |
| EFEMP1 | Q12805 |
| KRT17 | Q04695 |
| LCT | P09848 |
| TLR8 | Q9NR97 |
| MBD2 | Q9UBB5 |
| RPL3 | P39023 |
| LRP2 | P98164 |
| HMBS | P08397 |
| PPIA | P62937 |
| SEC23B | Q15437 |
| SLC12A6 | Q9UHW9 |
| RFWD3 | Q6PCD5 |
| ELK1 | P19419 |
| GAD1 | Q99259 |
| DYNC1H1 | Q14204 |
| NEK2 | P51955 |
| TRIM21 | P19474 |
| DCLRE1C | Q96SD1 |
| NOX4 | Q9NPH5 |
| CUL4A | Q13619 |
| RPS15 | P62841 |
| PLA2G7 | Q13093 |
| ITGA2B | P08514 |
| COPS5 | Q92905 |
| ARID2 | Q68CP9 |
| OPRM1 | P35372 |
| HAVCR1 | Q96D42 |
| PROCR | Q9UNN8 |
| DNAJB6 | O75190 |
| NAT10 | Q9H0A0 |
| PYGM | P11217 |
| CTSK | P43235 |
| TRIM33 | Q9UPN9 |
| RPS29 | P62273 |
| CTSA | P10619 |
| EIF2S1 | P05198 |
| GJA5 | P36382 |
| NSMF | Q6X4W1 |
| ALCAM | Q13740 |
| PIK3R2 | O00459 |
| PROC | P04070 |
| ROBO1 | Q9Y6N7 |
| HSD17B12 | Q53GQ0 |
| BCHE | P06276 |
| PAPPA-AS1 | Q5QFB9 |
| MYOM1 | P52179 |
| MCM2 | P49736 |
| ENO1 | P06733 |
| EIF2AK4 | Q9P2K8 |
| NUF2 | Q9BZD4 |
| LAMB3 | Q13751 |
| POLR3K | Q9Y2Y1 |
| CABLES1 | Q8TDN4 |
| AQP5 | P55064 |
| EEF1A2 | Q05639 |
| PGRMC2 | O15173 |
| SLC11A2 | P49281 |
| LRP4 | O75096 |
| DIAPH1 | O60610 |
| GREB1L | Q9C091 |
| RPS14 | P62263 |
| SEMA3E | O15041 |
| RPS6KA3 | P51812 |
| DEFB4A | O15263 |
| ADCYAP1 | P18509 |
| RACK1 | P63244 |
| XDH | P47989 |
| FBL | P22087 |
| PLCE1 | Q9P212 |
| OLR1 | P78380 |
| ALG13 | Q9NP73 |
| TAF4B | Q92750 |
| GNE | Q9Y223 |
| PAK2 | Q13177 |
| RECQL5 | O94762 |
| CALM3 | P0DP25 |
| DHX37 | Q8IY37 |
| SNAP25 | P60880 |
| PROK1 | P58294 |
| CNTNAP2 | Q9UHC6 |
| POLR2B | P30876 |
| PLOD2 | O00469 |
| THRB | P10828 |
| SLC4A1 | P02730 |
| G6PC1 | P35575 |
| EDA | Q92838 |
| ATF4 | P18848 |
| UGT1A8 | Q9HAW9 |
| PNKP | Q96T60 |
| STIL | Q15468 |
| COLQ | Q9Y215 |
| IARS2 | Q9NSE4 |
| LAMA3 | Q16787 |
| BAK1 | Q16611 |
| NEXMIF | Q5QGS0 |
| NR4A1 | P22736 |
| RPL38 | P63173 |
| PRDX1 | Q06830 |
| PHGDH | O43175 |
| CYSLTR1 | Q9Y271 |
| ETV6 | P41212 |
| LAMA4 | Q16363 |
| POLH | Q9Y253 |
| RBX1 | P62877 |
| BID | P55957 |
| ATP5PO | P48047 |
| PHLDA2 | Q53GA4 |
| ATF2 | P15336 |
| PAX9 | P55771 |
| PSAP | P07602 |
| CFAP47 | Q6ZTR5 |
| SF3B2 | Q13435 |
| IKZF1 | Q13422 |
| PRDM2 | Q13029 |
| PLAA | Q9Y263 |
| PRDX5 | P30044 |
| FARS2 | O95363 |
| MT-ND4 | P03905 |
| SERPING1 | P05155 |
| MDM4 | O15151 |
| CUL7 | Q14999 |
| MKKS | Q9NPJ1 |
| ISG15 | P05161 |
| CLN3 | Q13286 |
| FGB | P02675 |
| SRF | P11831 |
| CADM1 | Q9BY67 |
| IARS1 | P41252 |
| CHRNE | Q04844 |
| EIF2AK2 | P19525 |
| FCGR3A | P08637 |
| LRP6 | O75581 |
| YWHAZ | P63104 |
| TACC3 | Q9Y6A5 |
| TSR1 | Q2NL82 |
| S100A4 | P26447 |
| TAGLN | Q01995 |
| STIM1 | Q13586 |
| PTH2R | P49190 |
| GFI1 | Q99684 |
| MMUT | P22033 |
| RXRA | P19793 |
| PROK2 | Q9HC23 |
| DKK3 | Q9UBP4 |
| ATP2B3 | Q16720 |
| SGCB | Q16585 |
| CCL4 | P13236 |
| PANX1 | Q96RD7 |
| DDR1 | Q08345 |
| ASCC1 | Q8N9N2 |
| ITK | Q08881 |
| RBL2 | Q08999 |
| NTF4 | P34130 |
| ENPP2 | Q13822 |
| ADORA2A | P29274 |
| ALG3 | Q92685 |
| TNNI2 | P48788 |
| TRB | P0DSE2 |
| GSC | P56915 |
| SYNM | O15061 |
| MYB | P10242 |
| SIX1 | Q15475 |
| GSS | P48637 |
| ANOS1 | P23352 |
| HEXB | P07686 |
| WEE1 | P30291 |
| DELEC1 | Q9P2X7 |
| TFF3 | Q07654 |
| EEF2 | P13639 |
| MATK | P42679 |
| MYF6 | P23409 |
| HHIP | Q96QV1 |
| PDS5B | Q9NTI5 |
| PML | P29590 |
| BIRC3 | Q13489 |
| PLA2G4A | P47712 |
| UCP1 | P25874 |
| PCM1 | Q15154 |
| GALNT3 | Q14435 |
| CUL4B | Q13620 |
| OSM | P13725 |
| ASH1L | Q9NR48 |
| IQGAP2 | Q13576 |
| EIF4G1 | Q04637 |
| RIT1 | Q92963 |
| PPP3CA | Q08209 |
| SMARCE1 | Q969G3 |
| UQCC6 | Q69YU5 |
| CXCL2 | P19875 |
| RAB3GAP1 | Q15042 |
| CCNH | P51946 |
| FERMT1 | Q9BQL6 |
| EXOSC10 | Q01780 |
| CDON | Q4KMG0 |
| PODXL | O00592 |
| AXL | P30530 |
| HOXD13 | P35453 |
| RAB5A | P20339 |
| GCM2 | O75603 |
| SON | P18583 |
| CHD3 | Q12873 |
| NEUROD1 | Q13562 |
| OPA1 | O60313 |
| CEP57 | Q86XR8 |
| EGR2 | P11161 |
| CASK | O14936 |
| CDCA7 | Q9BWT1 |
| KLF4 | O43474 |
| USP9X | Q93008 |
| CYB561 | P49447 |
| HK2 | P52789 |
| SH2D1A | O60880 |
| ADAMTSL1 | Q8N6G6 |
| PC | P11498 |
| ADH1B | P00325 |
| ACAD9 | Q9H845 |
| FRS2 | Q8WU20 |
| EMP2 | P54851 |
| BCL11A | Q9H165 |
| AIF1 | P55008 |
| HLA-E | P13747 |
| TK1 | P04183 |
| EDN2 | P20800 |
| FZR1 | Q9UM11 |
| FUT2 | Q10981 |
| KIF14 | Q15058 |
| TPX2 | Q9ULW0 |
| HCP5 | Q6MZN7 |
| HOXD9 | P28356 |
| BBS4 | Q96RK4 |
| MAP3K7 | O43318 |
| CTBP1 | Q13363 |
| BIN1 | O00499 |
| CD163 | Q86VB7 |
| MARS2 | Q96GW9 |
| ANKRD39 | Q53RE8 |
| ABCC4 | O15439 |
| STK4 | Q13043 |
| ARSA | P15289 |
| HSD17B2 | P37059 |
| FOXA2 | Q9Y261 |
| IGF2BP2 | Q9Y6M1 |
| CD151 | P48509 |
| TUSC3 | Q13454 |
| RAD21L1 | Q9H4I0 |
| LPP | Q93052 |
| POLR2F | P61218 |
| RREB1 | Q92766 |
| MPI | P34949 |
| PIGT | Q969N2 |
| CRABP1 | P29762 |
| TRIM25 | Q14258 |
| FOSL1 | P15407 |
| AHI1 | Q8N157 |
| TMEM70 | Q9BUB7 |
| SATB1 | Q01826 |
| ERVW-1 | Q9UQF0 |
| ADCY10 | Q96PN6 |
| TCTN2 | Q96GX1 |
| PDSS1 | Q5T2R2 |
| GRHL3 | Q8TE85 |
| SREBF1 | P36956 |
| SIRT3 | Q9NTG7 |
| COPB2 | P35606 |
| UCP2 | P55851 |
| TSPAN32 | Q96QS1 |
| GAD2 | Q05329 |
| MC1R | Q01726 |
| HDAC3 | O15379 |
| KDSR | Q06136 |
| USF1 | P22415 |
| SPRY1 | O43609 |
| AHSG | P02765 |
| NCOA1 | Q15788 |
| PTGER3 | P43115 |
| SUN2 | Q9UH99 |
| PTGER4 | P35408 |
| SYK | P43405 |
| DPM1 | O60762 |
| TUBGCP6 | Q96RT7 |
| EFNA4 | P52798 |
| ARHGAP31 | Q2M1Z3 |
| CD63 | P08962 |
| GOLPH3 | Q9H4A6 |
| CHRNA1 | P02708 |
| INTS11 | Q5TA45 |
| MMP10 | P09238 |
| FUCA1 | P04066 |
| MC3R | P41968 |
| PSMC5 | P62195 |
| XBP1 | P17861 |
| SPRY4 | Q9C004 |
| SULT2A1 | Q06520 |
| NOP56 | O00567 |
| DNAH5 | Q8TE73 |
| HAND2 | P61296 |
| AGRN | O00468 |
| HEY2 | Q9UBP5 |
| SHROOM3 | Q8TF72 |
| SPART | Q8N0X7 |
| CSNK2A1 | P68400 |
| RAP1A | P62834 |
| CCT7 | Q99832 |
| KLF5 | Q13887 |
| HSP90AB1 | P08238 |
| RPS3 | P23396 |
| AICDA | Q9GZX7 |
| TGFBI | Q15582 |
| CPT1A | P50416 |
| ARX | Q96QS3 |
| NT5E | P21589 |
| FBP1 | P09467 |
| DEPDC5 | O75140 |
| DCAF8 | Q5TAQ9 |
| RPS15A | P62244 |
| ELP1 | O95163 |
| CHRNB1 | P11230 |
| GAS6 | Q14393 |
| SHOX2 | O60902 |
| HTT | P42858 |
| MYO18B | Q8IUG5 |
| DHCR24 | Q15392 |
| INCENP | Q9NQS7 |
| BAD | Q92934 |
| NEDD4 | P46934 |
| B9D2 | Q9BPU9 |
| FECH | P22830 |
| CBX1 | P83916 |
| ESM1 | Q9NQ30 |
| ID3 | Q02535 |
| NFATC1 | O95644 |
| MAGI2 | Q86UL8 |
| AP2B1 | P63010 |
| CANX | P27824 |
| ODC1 | P11926 |
| TRDMT1 | O14717 |
| SKIL | P12757 |
| BMP10 | O95393 |
| HTR3A | P46098 |
| WIF1 | Q9Y5W5 |
| HSF1 | Q00613 |
| RPL18 | Q07020 |
| DNMT3L | Q9UJW3 |
| GJC2 | Q5T442 |
| KIF23 | Q02241 |
| TUBB6 | Q9BUF5 |
| MUC5B | Q9HC84 |
| UTP4 | Q969X6 |
| DRD1 | P21728 |
| CNBP | P62633 |
| IFI16 | Q16666 |
| BMP1 | P13497 |
| B4GALT1 | P15291 |
| TMEM98 | Q9Y2Y6 |
| WDR73 | Q6P4I2 |
| CRHR2 | Q13324 |
| CBX5 | P45973 |
| ADAMTSL4 | Q6UY14 |
| CEP85L | Q5SZL2 |
| DRD4 | P21917 |
| CSK | P41240 |
| TMPO | P42166 |
| TYK2 | P29597 |
| SIRT7 | Q9NRC8 |
| PABPN1 | Q86U42 |
| DIS3 | Q9Y2L1 |
| TRO | Q12816 |
| PRDX2 | P32119 |
| RAB7A | P51149 |
| SLC7A7 | Q9UM01 |
| VLDLR | P98155 |
| BIRC2 | Q13490 |
| PTGFR | P43088 |
| OSGEP | Q9NPF4 |
| SMAD5 | Q99717 |
| MLKL | Q8NB16 |
| LEF1 | Q9UJU2 |
| TNFSF13B | Q9Y275 |
| PTPA | Q15257 |
| MTHFS | P49914 |
| HAVCR2 | Q8TDQ0 |
| NAP1L4 | Q99733 |
| PRKAA1 | Q13131 |
| PPP1CB | P62140 |
| METTL3 | Q86U44 |
| PDIA3 | P30101 |
| SOD3 | P08294 |
| ID1 | P41134 |
| IL23A | Q9NPF7 |
| PRKCZ | Q05513 |
| PUF60 | Q9UHX1 |
| CHRNA7 | P36544 |
| CTTN | Q14247 |
| STRA6 | Q9BX79 |
| NRXN1 | Q9ULB1 |
| C12orf57 | Q99622 |
| DPP3 | Q9NY33 |
| EHMT2 | Q96KQ7 |
| TFPI | P10646 |
| CASP14 | P31944 |
| WNK4 | Q96J92 |
| SLC1A5 | Q15758 |
| RPS6 | P62753 |
| SLC5A1 | P13866 |
| ITIH2 | P19823 |
| GAL | P22466 |
| GLUD1 | P00367 |
| CAPN2 | P17655 |
| IRAK1 | P51617 |
| CDK5RAP2 | Q96SN8 |
| HDAC6 | Q9UBN7 |
| EFTUD2 | Q15029 |
| UGDH | O60701 |
| TUBG1 | P23258 |
| SLC10A2 | Q12908 |
| PDE4D | Q08499 |
| RRM2 | P31350 |
| CCDC47 | Q96A33 |
| P2RY12 | Q9H244 |
| SSTR3 | P32745 |
| PRKAA2 | P54646 |
| KDM2B | Q8NHM5 |
| ADORA1 | P30542 |
| RBL1 | P28749 |
| RPL35 | P42766 |
| HSPA9 | P38646 |
| WASHC5 | Q12768 |
| HLCS | P50747 |
| MEIG1 | Q5JSS6 |
| MSI2 | Q96DH6 |
| KLLN | B2CW77 |
| LOXL2 | Q9Y4K0 |
| EIF2AK1 | Q9BQI3 |
| TCF3 | P15923 |
| IL18R1 | Q13478 |
| RNASE2 | P10153 |
| AEBP1 | Q8IUX7 |
| LACTB | P83111 |
| LAGE3 | Q14657 |
| MAMLD1 | Q13495 |
| BBS5 | Q8N3I7 |
| H4C1 | P62805 |
| HNRNPH2 | P55795 |
| CXCL3 | P19876 |
| ABCD3 | P28288 |
| NBAS | A2RRP1 |
| CCL20 | P78556 |
| PSMB2 | P49721 |
| RPGR | Q92834 |
| YWHAQ | P27348 |
| B9D1 | Q9UPM9 |
| SLIT2 | O94813 |
| UBE2I | P63279 |
| UTRN | P46939 |
| CARD11 | Q9BXL7 |
| TLR1 | Q15399 |
| DNTT | P04053 |
| CD68 | P34810 |
| RPSA | P08865 |
| CBX3 | Q13185 |
| APOC1 | P02654 |
| SPTBN1 | Q01082 |
| IL17F | Q96PD4 |
| MTMR10 | Q9NXD2 |
| STAC3 | Q96MF2 |
| TUBGCP2 | Q9BSJ2 |
| NTF3 | P20783 |
| HPSE2 | Q8WWQ2 |
| MSI1 | O43347 |
| CNR1 | P21554 |
| TYRO3 | Q06418 |
| BLVRB | P30043 |
| FUT1 | P19526 |
| ABO | P16442 |
| CPOX | P36551 |
| BBS2 | Q9BXC9 |
| SPTLC1 | O15269 |
| SPINK5 | Q9NQ38 |
| EEFSEC | P57772 |
| HADH | Q16836 |
| RAB8A | P61006 |
| LAMA5 | O15230 |
| SERPINB3 | P29508 |
| TLE6 | Q9H808 |
| NEDD4L | Q96PU5 |
| STXBP2 | Q15833 |
| SMURF1 | Q9HCE7 |
| TRADD | Q15628 |
| PTPN6 | P29350 |
| EPHA3 | P29320 |
| SCG5 | P05408 |
| HBG1 | P69891 |
| KCNJ8 | Q15842 |
| PLS3 | P13797 |
| PPBP | P02775 |
| PSMB4 | P28070 |
| NHEJ1 | Q9H9Q4 |
| DAXX | Q9UER7 |
| HSPB8 | Q9UJY1 |
| DDX41 | Q9UJV9 |
| SRP72 | O76094 |
| RPL22 | P35268 |
| GCDH | Q92947 |
| TCN1 | P20061 |
| CYP2R1 | Q6VVX0 |
| HOXA13 | P31271 |
| PDYN | P01213 |
| ETS2 | P15036 |
| MOGS | Q13724 |
| LUC7L2 | Q9Y383 |
| EPHA1 | P21709 |
| GCN1 | Q92616 |
| FABP5 | Q01469 |
| EPX | P11678 |
| PTGDS | P41222 |
| PKN1 | Q16512 |
| AKR1C2 | P52895 |
| GNB3 | P16520 |
| TUFM | P49411 |
| CGB7 | P0DN87 |
| HNMT | P50135 |
| FNDC5 | Q8NAU1 |
| PLEK | P08567 |
| DSE | Q9UL01 |
| PKLR | P30613 |
| ABHD5 | Q8WTS1 |
| TTPA | P49638 |
| PRKCSH | P14314 |
| TNFAIP6 | P98066 |
| CAVIN1 | Q6NZI2 |
| TIMM50 | Q3ZCQ8 |
| GAR1 | Q9NY12 |
| PEPD | P12955 |
| AP4M1 | O00189 |
| GATD3 | P0DPI2 |
| PRPF8 | Q6P2Q9 |
| KRT84 | Q9NSB2 |
| UBTF | P17480 |
| RBFOX2 | O43251 |
| DCX | O43602 |
| BLZF1 | Q9H2G9 |
| SUPT5H | O00267 |
| ACVR1B | P36896 |
| CDK8 | P49336 |
| NFKBIB | Q15653 |
| TOP3A | Q13472 |
| PSAT1 | Q9Y617 |
| RBBP4 | Q09028 |
| LYST | Q99698 |
| NUP153 | P49790 |
| ARSL | P51690 |
| SOX18 | P35713 |
| COL27A1 | Q8IZC6 |
| HSP90B1 | P14625 |
| IFNGR2 | P38484 |
| MT-ND4L | P03901 |
| IFNAR1 | P17181 |
| MAP1LC3A | Q9H492 |
| CACNA1A | O00555 |
| TUBB4A | P04350 |
| CD70 | P32970 |
| CXCL16 | Q9H2A7 |
| MTA3 | Q9BTC8 |
| PSMB9 | P28065 |
| TLR6 | Q9Y2C9 |
| COL10A1 | Q03692 |
| RBBP7 | Q16576 |
| BRAT1 | Q6PJG6 |
| FMN2 | Q9NZ56 |
| HMGB2 | P26583 |
| PRKCQ | Q04759 |
| SSTR5 | P35346 |
| NDUFS2 | O75306 |
| ATP1A1 | P05023 |
| JARID2 | Q92833 |
| CAPN1 | P07384 |
| PDE4A | P27815 |
| MAP4K4 | O95819 |
| POLR3B | Q9NW08 |
| LMOD1 | P29536 |
| TLX1NB | P0CAT3 |
| AHCY | P23526 |
| GALNS | P34059 |
| IL16 | Q14005 |
| SNHG12 | Q9BXW3 |
| WNT10B | O00744 |
| CTSG | P08311 |
| NR2F2 | P24468 |
| BBC3 | Q96PG8 |
| THRA | P10827 |
| AKAP13 | Q12802 |
| ACVR1C | Q8NER5 |
| NAP1L1 | P55209 |
| GALK2 | Q01415 |
| IAPP | P10997 |
| CRYBA4 | P53673 |
| SLC4A2 | P04920 |
| SCN9A | Q15858 |
| SAG | P10523 |
| PELI1 | Q96FA3 |
| CDT1 | Q9H211 |
| PPP1R12A | O14974 |
| CUL3 | Q13618 |
| PSMC2 | P35998 |
| PPP1R15A | O75807 |
| FGF19 | O95750 |
| CD9 | P21926 |
| PDP1 | Q9P0J1 |
| TACR1 | P25103 |
| AKAP1 | Q92667 |
| SNAP29 | O95721 |
| FOXC1 | Q12948 |
| DMRT2 | Q9Y5R5 |
| EPHX2 | P34913 |
| GPI | P06744 |
| EIF2S3 | P41091 |
| DHX30 | Q7L2E3 |
| SERPINB7 | O75635 |
| DLX4 | Q92988 |
| GNAQ | P50148 |
| POLR1A | O95602 |
| FIBP | O43427 |
| EIF1 | P41567 |
| FMO3 | P31513 |
| CD27 | P26842 |
| VASH1 | Q7L8A9 |
| TBX3 | O15119 |
| STIP1 | P31948 |
| KHDRBS3 | O75525 |
| AFG3L2 | Q9Y4W6 |
| TRPV6 | Q9H1D0 |
| ACP1 | P24666 |
| VARS2 | Q5ST30 |
| VAX1 | Q5SQQ9 |
| ZAP70 | P43403 |
| TNIP1 | Q15025 |
| DLX5 | P56178 |
| PRRT2 | Q7Z6L0 |
| MAOA | P21397 |
| FER1L6 | Q2WGJ9 |
| SYCE2 | Q6PIF2 |
| CXorf38 | Q8TB03 |
| DMRTC1 | Q5HYR2 |
| SBF2 | Q86WG5 |
| SLC22A2 | O15244 |
| DDRGK1 | Q96HY6 |
| GJA8 | P48165 |
| LRG1 | P02750 |
| MNX1 | P50219 |
| APOD | P05090 |
| MGAM | O43451 |
| HJURP | Q8NCD3 |
| TET1 | Q8NFU7 |
| RPS4Y1 | P22090 |
| SLC9A1 | P19634 |
| PRMT1 | Q99873 |
| SLC5A6 | Q9Y289 |
| H2BC21 | Q16778 |
| CLIC1 | O00299 |
| PRPF4 | O43172 |
| FGG | P02679 |
| BACH1 | O14867 |
| DTL | Q9NZJ0 |
| CX3CR1 | P49238 |
| SH3TC2 | Q8TF17 |
| CCL22 | O00626 |
| COL4A2 | P08572 |
| SCARF2 | Q96GP6 |
| CLTC | Q00610 |
| MCM3 | P25205 |
| TAC1 | P20366 |
| KAT6B | Q8WYB5 |
| HSPB6 | O14558 |
| ITGA8 | P53708 |
| NDUFS3 | O75489 |
| PRNP | F7VJQ1 |
| TMEM138 | Q9NPI0 |
| SRSF1 | Q07955 |
| CCAR2 | Q8N163 |
| WARS2 | Q9UGM6 |
| TSPO | B1AH88 |
| SPRED1 | Q7Z699 |
| TSEN34 | Q9BSV6 |
| DVL1 | O14640 |
| SLC3A2 | P08195 |
| HAS1 | Q92839 |
| UNC13D | Q70J99 |
| MCM5 | P33992 |
| DNAJA2 | O60884 |
| P4HA2 | O15460 |
| PPP1R15B | Q5SWA1 |
| BBS12 | Q6ZW61 |
| XAF1 | Q6GPH4 |
| TRPC3 | Q13507 |
| AQP9 | O43315 |
| COL9A1 | P20849 |
| IVL | P07476 |
| SEC63 | Q9UGP8 |
| CENPA | P49450 |
| TERF2IP | Q9NYB0 |
| MN1 | Q10571 |
| GIP | P09681 |
| HCCS | P53701 |
| ADAMTS16 | Q8TE57 |
| RNPC3 | Q96LT9 |
| ISL1 | P61371 |
| ATF6 | P18850 |
| PSMC4 | P43686 |
| STAG2 | Q8N3U4 |
| TET3 | O43151 |
| SLAMF1 | Q13291 |
| RALGDS | Q12967 |
| SLC30A8 | Q8IWU4 |
| CLDN10 | P78369 |
| TMEM237 | Q96Q45 |
| SSTR2 | P30874 |
| PMEL | P40967 |
| CHD5 | Q8TDI0 |
| LYZ | P61626 |
| ACOT11 | Q8WXI4 |
| LOXL3 | P58215 |
| HSPE1 | P61604 |
| TAPBP | O15533 |
| TRIM71 | Q2Q1W2 |
| CX3CL1 | P78423 |
| H2AC20 | Q16777 |
| DGUOK | Q16854 |
| ARL13B | Q3SXY8 |
| TRIM28 | Q13263 |
| A2ML1 | A8K2U0 |
| PSMD14 | O00487 |
| MYL3 | P08590 |
| MMADHC | Q9H3L0 |
| IFT88 | Q13099 |
| LHX1 | P48742 |
| ASPA | P45381 |
| SAE1 | Q9UBE0 |
| RAB11A | P62491 |
| SLCO1B1 | Q9Y6L6 |
| B3GLCT | Q6Y288 |
| GBA2 | Q9HCG7 |
| ADAMTS9 | Q9P2N4 |
| GLIS3 | Q8NEA6 |
| UBA52 | P62987 |
| FTL | P02792 |
| SCYL1 | Q96KG9 |
| RPL31 | P62899 |
| FOSL2 | P15408 |
| CISD2 | Q8N5K1 |
| CTR9 | Q6PD62 |
| ZFX | P17010 |
| KIF17 | Q9P2E2 |
| NUP210 | Q8TEM1 |
| SNRPE | P62304 |
| ARSH | Q5FYA8 |
| FGF17 | O60258 |
| TXNRD1 | Q16881 |
| COL17A1 | Q9UMD9 |
| STING1 | Q86WV6 |
| INPPL1 | O15357 |
| FAF1 | Q9UNN5 |
| AQP4 | P55087 |
| SUCLA2 | Q9P2R7 |
| HBE1 | P02100 |
| SLCO1B3 | Q9NPD5 |
| ESPL1 | Q14674 |
| MAVS | Q7Z434 |
| ZMYND10 | O75800 |
| TRIM32 | Q13049 |
| ROS1 | P08922 |
| KIF3A | Q9Y496 |
| DAZ1 | Q9NQZ3 |
| GLUL | P15104 |
| EIF3E | P60228 |
| IGFBP7 | Q16270 |
| IRX2-DT | Q86SI9 |
| RAD23B | P54727 |
| UBE2D3 | P61077 |
| H4C16 | P62805 |
| PLIN1 | O60240 |
| MAP2K6 | P52564 |
| SSTR1 | P30872 |
| RNF2 | Q99496 |
| PNPLA6 | Q8IY17 |
| BCL7C | Q8WUZ0 |
| PAX4 | O43316 |
| IRF7 | Q92985 |
| ASIP | P42127 |
| CD1C | P29017 |
| NEFL | P07196 |
| NUMA1 | Q14980 |
| VKORC1 | Q9BQB6 |
| NHLH2 | Q02577 |
| NPR3 | P17342 |
| PYCARD | Q9ULZ3 |
| SNX10 | Q9Y5X0 |
| RHEB | Q15382 |
| SNRPB | P14678 |
| PSMB5 | P28074 |
| BST1 | Q10588 |
| PLD2 | O14939 |
| LRRC59 | Q96AG4 |
| ANXA4 | P09525 |
| SMPD4 | Q9NXE4 |
| ATP5F1A | P25705 |
| TFE3 | P19532 |
| COQ6 | Q9Y2Z9 |
| IL18BP | O95998 |
| NME2 | P22392 |
| DUSP19 | Q8WTR2 |
| RRM1 | P23921 |
| DOCK8 | Q8NF50 |
| ESX1 | Q8N693 |
| DCAF1 | Q9Y4B6 |
| POLQ | O75417 |
| LHX3 | Q9UBR4 |
| SLC35A1 | P78382 |
| ABCA7 | Q8IZY2 |
| PLAG1 | Q6DJT9 |
| PPP2R3C | Q969Q6 |
| PTS | Q03393 |
| TCEAL1 | Q15170 |
| ITLN1 | Q8WWA0 |
| RPGRIP1 | Q96KN7 |
| AQP3 | Q92482 |
| SLC40A1 | Q9NP59 |
| DNAJA1 | P31689 |
| NCOA6 | Q14686 |
| KLF1 | Q13351 |
| FPGS | Q05932 |
| DHPS | P49366 |
| HDAC4 | P56524 |
| KAT2B | Q92831 |
| TIA1 | P31483 |
| RUNX1T1 | Q06455 |
| ENPEP | Q07075 |
| TNFSF13 | O75888 |
| NDUFA13 | Q9P0J0 |
| CYBB | P04839 |
| MAN1B1 | Q9UKM7 |
| OLIG2 | Q13516 |
| NDUFA9 | Q16795 |
| SIAH1 | Q8IUQ4 |
| MSMB | P08118 |
| RPN1 | P04843 |
| KRT6A | P02538 |
| SUV39H1 | O43463 |
| RPS18 | P62269 |
| DCANP1 | Q8TF63 |
| MT-CO3 | P00414 |
| VSX2 | P58304 |
| SCLT1 | Q96NL6 |
| FKBP8 | Q14318 |
| TRAF1 | Q13077 |
| NIPAL4 | Q0D2K0 |
| KPNB1 | Q14974 |
| UBE2T | Q9NPD8 |
| DEFB103B | P81534 |
| BTC | P35070 |
| RBM8A | Q9Y5S9 |
| IQGAP1 | P46940 |
| MMP26 | Q9NRE1 |
| HSD17B6 | O14756 |
| HUS1 | O60921 |
| CKS1B | P61024 |
| RAD18 | Q9NS91 |
| H3C14 | Q71DI3 |
| UGP2 | Q16851 |
| ZIC3 | O60481 |
| CLIP1 | P30622 |
| MRPL50 | Q8N5N7 |
| HOXA4 | Q00056 |
| MMAA | Q8IVH4 |
| SP100 | P23497 |
| YME1L1 | Q96TA2 |
| JUNB | P17275 |
| GDAP1 | Q8TB36 |
| CDCA5 | Q96FF9 |
| UCN | P55089 |
| KDM5C | P41229 |
| CERS2 | Q96G23 |
| CARM1 | Q86X55 |
| PRKCI | P41743 |
| GDF6 | Q6KF10 |
| SLC6A8 | P48029 |
| EPM2A | B3EWF7 |
| RPL7A | P62424 |
| TIMP4 | Q99727 |
| MDK | P21741 |
| AZGP1 | P25311 |
| CCR9 | P51686 |
| BST2 | Q10589 |
| NHS | Q6T4R5 |
| ID2 | Q02363 |
| SPOCK2 | Q92563 |
| SLC4A4 | Q9Y6R1 |
| TOP2B | Q02880 |
| HNRNPH1 | P31943 |
| PRMT5 | O14744 |
| TACC1 | O75410 |
| P2RX7 | Q99572 |
| PXDN | Q92626 |
| BAIAP2L1 | Q9UHR4 |
| LEFTY2 | O00292 |
| LGR5 | O75473 |
| MTMR2 | Q13614 |
| TMEM199 | Q8N511 |
| NDUFS4 | O43181 |
| SCAPER | Q9BY12 |
| CDK9 | P50750 |
| NLK | Q9UBE8 |
| YWHAE | P62258 |
| TRIM8 | Q9BZR9 |
| NOP58 | Q9Y2X3 |
| BNIP3 | Q12983 |
| AADAC | P22760 |
| PORCN | Q9H237 |
| ETV5 | P41161 |
| CLDN1 | O95832 |
| NEBL | O76041 |
| UVSSA | Q2YD98 |
| CNN1 | P51911 |
| ALOX15B | O15296 |
| IL27 | Q8NEV9 |
| RDX | P35241 |
| PEBP1 | P30086 |
| CD177 | Q8N6Q3 |
| TIMMDC1 | Q9NPL8 |
| RPL21 | P46778 |
| TSEN2 | Q8NCE0 |
| THRAP3 | Q9Y2W1 |
| PIKFYVE | Q9Y2I7 |
| IRF2 | P14316 |
| BCL2A1 | Q16548 |
| COQ2 | Q96H96 |
| FXYD2 | P54710 |
| TRA | P0DSE1 |
| KRT6B | P04259 |
| PSIP1 | O75475 |
| RBM10 | P0DW28 |
| DPH2 | Q9BQC3 |
| NR1I3 | Q14994 |
| CNP | P09543 |
| MRPL23 | Q16540 |
| SP3 | Q02447 |
| A1CF | Q9NQ94 |
| PSMA5 | P28066 |
| VRK2 | Q86Y07 |
| PFKFB3 | Q16875 |
| UCP3 | P55916 |
| CR1 | P17927 |
| RXRB | P28702 |
| PENK | P01210 |
| FCGR2B | P31994 |
| VAPB | O95292 |
| EIF4A3 | P38919 |
| CALD1 | Q05682 |
| ROBO3 | Q96MS0 |
| ALDH9A1 | P49189 |
| LRRC32 | Q14392 |
| TPP1 | O14773 |
| RAB3GAP2 | Q9H2M9 |
| ARMC5 | Q96C12 |
| ATL1 | Q8WXF7 |
| CARD9 | Q9H257 |
| CYP3A7 | P24462 |
| SLIT3 | O75094 |
| RARG | P13631 |
| CFB | P00751 |
| INHBC | P55103 |
| PPFIBP1 | Q86W92 |
| REPIN1 | Q9BWE0 |
| NR1H3 | Q13133 |
| EPHB1 | P54762 |
| PIN1 | Q13526 |
| LMX1A | Q8TE12 |
| MYO6 | Q9UM54 |
| PRKRA | O75569 |
| PLA2G2A | P14555 |
| AMPH | P49418 |
| NFATC2 | Q13469 |
| HIP1 | O00291 |
| AGGF1 | Q8N302 |
| NEK9 | Q8TD19 |
| MYOCD | Q8IZQ8 |
| DNAH11 | Q96DT5 |
| TOE1 | Q96GM8 |
| PRDX3 | P30048 |
| GART | P22102 |
| A2M | P01023 |
| PIGR | P01833 |
| WNT7B | P56706 |
| ADCYAP1R1 | P41586 |
| BFSP2 | Q13515 |
| OGT | O15294 |
| RARRES2 | Q99969 |
| ATP5F1B | P06576 |
| ARRB2 | P32121 |
| CNTF | P26441 |
| M6PR | P20645 |
| CETN2 | P41208 |
| RIPK3 | Q9Y572 |
| KRIT1 | O00522 |
| AGO2 | Q9UKV8 |
| RNF7 | Q9UBF6 |
| ARHGAP24 | Q8N264 |
| MAP2K3 | P46734 |
| SLC25A5 | P05141 |
| ANTXR2 | P58335 |
| KLF2 | Q9Y5W3 |
| KLB | Q86Z14 |
| NLRP7 | Q8WX94 |
| ABCA13 | Q86UQ4 |
| PPP1R3A | Q16821 |
| IRF3 | Q14653 |
| SETBP1 | Q9Y6X0 |
| GPX1 | P07203 |
| PRPH2 | P23942 |
| TOR1AIP1 | Q5JTV8 |
| PMPCB | O75439 |
| TRPV1 | Q8NER1 |
| LEPROTL1 | O95214 |
| AKAP12 | Q02952 |
| KCNK9 | Q9NPC2 |
| LGALS7B | P47929 |
| SLC12A5 | Q9H2X9 |
| GCSH | P23434 |
| ADORA2B | P29275 |
| KLK15 | Q9H2R5 |
| LCN1 | P31025 |
| RPS2 | P15880 |
| ARG2 | P78540 |
| GPHN | Q9NQX3 |
| P4HTM | Q9NXG6 |
| COG6 | Q9Y2V7 |
| ACAT1 | P24752 |
| CRELD1 | Q96HD1 |
| TCTN1 | Q2MV58 |
| CHP2 | O43745 |
| STX6 | O43752 |
| MAP3K3 | Q99759 |
| TGM3 | Q08188 |
| PNPLA3 | Q9NST1 |
| SLC1A2 | P43004 |
| RDH12 | Q96NR8 |
| PADI4 | Q9UM07 |
| UAP1 | Q16222 |
| SACS | Q9NZJ4 |
| KDM5B | Q9UGL1 |
| H3-3A | P84243 |
| TRIM27 | P14373 |
| SLC12A7 | Q9Y666 |
| SAA4 | P35542 |
| SSBP1 | Q04837 |
| ARHGEF2 | Q92974 |
| GOLM1 | Q8NBJ4 |
| CHKB | Q9Y259 |
| CHRD | Q9H2X0 |
| TLX3 | O43711 |
| CYP2A6 | P11509 |
| LMAN1 | P49257 |
| POLR2H | P52434 |
| TTK | P33981 |
| MX1 | P20591 |
| SELENOP | P49908 |
| MTA2 | O94776 |
| HNRNPUL1 | Q9BUJ2 |
| CDK11A | Q9UQ88 |
| CLUAP1 | Q96AJ1 |
| APLNR | P35414 |
| CPLANE1 | Q9H799 |
| MIA2 | Q96PC5 |
| PHKA2 | P46019 |
| PSMD1 | Q99460 |
| GABBR2 | O75899 |
| TBX6 | O95947 |
| DSPP | Q9NZW4 |
| KRT16 | P08779 |
| SLC1A1 | P43005 |
| PPRC1 | Q5VV67 |
| SFPQ | P23246 |
| MRTFA | Q969V6 |
| MAOB | P27338 |
| NFIA | Q12857 |
| ALDH5A1 | P51649 |
| SRCAP | Q6ZRS2 |
| ADIPOR1 | Q96A54 |
| SNRNP200 | O75643 |
| SPINK13 | Q1W4C9 |
| PYCR2 | Q96C36 |
| SGCE | O43556 |
| THADA | Q6YHU6 |
| NAA50 | Q9GZZ1 |
| GDF3 | Q9NR23 |
| CLCN3 | P51790 |
| HPX | P02790 |
| PPP2R5C | Q13362 |
| KLKB1 | P03952 |
| SLC31A2 | O15432 |
| MFF | Q9GZY8 |
| TCEA1 | P23193 |
| ARL6 | Q9H0F7 |
| AP2M1 | Q96CW1 |
| CD99 | P14209 |
| CAND1 | Q86VP6 |
| EYS | Q5T1H1 |
| CFAP418 | Q96NL8 |
| CDH17 | Q12864 |
| DNM3 | Q9UQ16 |
| ALDOA | P04075 |
| PRIMPOL | Q96LW4 |
| EPHB2 | P29323 |
| ADRB1 | P08588 |
| IRF4 | Q15306 |
| IGFALS | P35858 |
| POLI | Q9UNA4 |
| RAD17 | O75943 |
| JAM3 | Q9BX67 |
| OPRK1 | P41145 |
| TYRP1 | P17643 |
| PROX1 | Q92786 |
| SLC16A4 | O15374 |
| PFN1 | P07737 |
| PFKL | P17858 |
| TNFRSF25 | Q93038 |
| PGAM5 | Q96HS1 |
| IVD | P26440 |
| TNKS | O95271 |
| RNF135 | Q8IUD6 |
| RANBP1 | P43487 |
| LGR4 | Q9BXB1 |
| BUB3 | O43684 |
| CRB1 | P82279 |
| RAC3 | P60763 |
| PARK7 | Q99497 |
| INS-IGF2 | F8WCM5 |
| LSP1 | P33241 |
| FOXO4 | P98177 |
| DRD3 | P35462 |
| CXCL5 | P42830 |
| SMC2 | O95347 |
| COG3 | Q96JB2 |
| ROCK2 | O75116 |
| SPG21 | Q9NZD8 |
| ATP6AP2 | O75787 |
| TNNT3 | P45378 |
| IFT20 | Q8IY31 |
| ZNF423 | Q2M1K9 |
| MICOS13 | Q5XKP0 |
| LIM2 | P55344 |
| SLC29A1 | Q99808 |
| DACH1 | Q9UI36 |
| CLDN11 | O75508 |
| THOC2 | Q8NI27 |
| MALT1 | Q9UDY8 |
| KTN1 | Q86UP2 |
| MT-CO2 | P00403 |
| HTR1B | P28222 |
| ANK3 | Q12955 |
| IER3IP1 | Q9Y5U9 |
| GPBAR1 | Q8TDU6 |
| YBX3 | P16989 |
| ILF3 | Q12906 |
| DPM3 | Q9P2X0 |
| HYAL1 | Q12794 |
| UQCRC2 | P22695 |
| HOXA5 | P20719 |
| RSAD2 | Q8WXG1 |
| H1-0 | P07305 |
| ERFE | Q4G0M1 |
| IDH3G | P51553 |
| ENTPD1 | P49961 |
| TJP2 | Q9UDY2 |
| ASXL3 | Q9C0F0 |
| GLDC | P23378 |
| HPR | P00739 |
| MARCKS | P29966 |
| AMELX | Q99217 |
| NUPR1 | O60356 |
| TOMM40 | O96008 |
| KIF21A | Q7Z4S6 |
| NUCB2 | P80303 |
| SIRT2 | Q8IXJ6 |
| CACNA1G | O43497 |
| GNAI1 | P63096 |
| ULK1 | O75385 |
| SOX4 | Q06945 |
| CEP19 | Q96LK0 |
| PRPF6 | O94906 |
| DEK | P35659 |
| WNT2 | P09544 |
| TIMM44 | O43615 |
| CD83 | Q01151 |
| RANBP2 | P49792 |
| CCL25 | O15444 |
| MYF5 | P13349 |
| IL1RAP | Q9NPH3 |
| TEFM | Q96QE5 |
| PARS2 | Q7L3T8 |
| PHB2 | Q99623 |
| LAMC3 | Q9Y6N6 |
| ADAR | P55265 |
| PSMD3 | O43242 |
| BCKDHA | P12694 |
| CCT6A | P40227 |
| SKP1 | P63208 |
| SERTAD1 | Q9UHV2 |
| GNL2 | Q13823 |
| MROH7 | Q68CQ1 |
| PSMD11 | O00231 |
| RLBP1 | P12271 |
| GNL3 | Q9BVP2 |
| TBL1XR1 | Q9BZK7 |
| MAP3K14 | Q99558 |
| RPS8 | P62241 |
| GABARAPL1 | Q9H0R8 |
| ECE1 | P42892 |
| EXOSC4 | Q9NPD3 |
| TUBB4B | P68371 |
| ORAI1 | Q96D31 |
| PCBP1 | Q15365 |
| ELOVL1 | Q9BW60 |
| UBE2A | P49459 |
| EXOSC6 | Q5RKV6 |
| GRHL2 | Q6ISB3 |
| ATP5F1C | P36542 |
| ARHGEF10 | O15013 |
| GGCX | P38435 |
| DOT1L | Q8TEK3 |
| NPC1L1 | Q9UHC9 |
| CLCN2 | P51788 |
| CACNA1H | O95180 |
| TXNIP | Q9H3M7 |
| PTGES2 | Q9H7Z7 |
| GNS | P15586 |
| ODAD1 | Q96M63 |
| PTPRN | Q16849 |
| KIF2A | O00139 |
| ALPK1 | Q96QP1 |
| COPA | P53621 |
| ABCA2 | Q9BZC7 |
| KAT5 | Q92993 |
| VAV1 | P15498 |
| FKBP1A | P62942 |
| TSLP | Q969D9 |
| BACH2 | Q9BYV9 |
| HRH2 | P25021 |
| AMER1 | Q5JTC6 |
| AGPS | O00116 |
| CDK5RAP3 | Q96JB5 |
| CDC27 | P30260 |
| NAA11 | Q9BSU3 |
| ARF6 | P62330 |
| OVGP1 | Q12889 |
| FGF5 | P12034 |
| SECISBP2 | Q96T21 |
| OAS3 | Q9Y6K5 |
| RBCK1 | Q9BYM8 |
| MAX | P61244 |
| CALCR | P30988 |
| DOCK1 | Q14185 |
| ONECUT2 | O95948 |
| PTGES3 | Q15185 |
| SYNPO | Q8N3V7 |
| PNP | P00491 |
| EPHB3 | P54753 |
| NRG3 | P56975 |
| WNT3 | P56703 |
| TNXA | Q16473 |
| NADSYN1 | Q6IA69 |
| PPP2R5D | Q14738 |
| AGA | P20933 |
| ALG6 | Q9Y672 |
| SCARA3 | Q6AZY7 |
| IGHG1 | P01857 |
| FBF1 | Q8TES7 |
| TSSC4 | Q9Y5U2 |
| UNC45A | Q9H3U1 |
| REV3L | O60673 |
| SOAT2 | O75908 |
| YWHAB | P31946 |
| VAMP8 | Q9BV40 |
| JAG2 | Q9Y219 |
| CUL5 | Q93034 |
| AMD1 | P17707 |
| RNASEH2A | O75792 |
| CCP110 | O43303 |
| CEP97 | Q8IW35 |
| SLC27A2 | O14975 |
| COL4A6 | Q14031 |
| HSPH1 | Q92598 |
| BTRC | Q9Y297 |
| TBC1D20 | Q96BZ9 |
| CYC1 | P08574 |
| EME1 | Q96AY2 |
| IGHV4-38-2 | P0DP08 |
| MYCNOS | P40205 |
| TDG | Q13569 |
| ANO1 | Q5XXA6 |
| SERPINF2 | P08697 |
| SULT1E1 | P49888 |
| SCO1 | O75880 |
| POLRMT | O00411 |
| XPOT | O43592 |
| RPS9 | P46781 |
| IFT70B | Q8N4P2 |
| WNT11 | O96014 |
| CAP1 | Q01518 |
| SALL2 | Q9Y467 |
| ICMT | O60725 |
| CRYGS | P22914 |
| BFSP1 | Q12934 |
| LGALS4 | P56470 |
| SPRED2 | Q7Z698 |
| KCNK2 | O95069 |
| PTPRO | Q16827 |
| TARS2 | Q9BW92 |
| SASH1 | O94885 |
| HSPA1L | P34931 |
| TM7SF3 | Q9NS93 |
| VSIG4 | Q9Y279 |
| MYOZ2 | Q9NPC6 |
| SMARCC2 | Q8TAQ2 |
| FCGRT | P55899 |
| MAP2 | P11137 |
| SPTBN2 | O15020 |
| SOAT1 | P35610 |
| TCL1A | P56279 |
| RPLP0 | P05388 |
| DDHD2 | O94830 |
| CLOCK | O15516 |
| BMPER | Q8N8U9 |
| KRT1 | P04264 |
| NAGS | Q8N159 |
| TNFRSF4 | P43489 |
| CD38 | P28907 |
| F11R | Q9Y624 |
| CLPX | O76031 |
| CDK5 | Q00535 |
| MBTPS1 | Q14703 |
| EEF1G | P26641 |
| SLC52A1 | Q9NWF4 |
| GPAM | Q9HCL2 |
| COA6 | Q5JTJ3 |
| SERPINA4 | P29622 |
| ANKRD49 | Q8WVL7 |
| BBS7 | Q8IWZ6 |
| CFC1 | P0CG37 |
| CEP192 | Q8TEP8 |
| MTREX | P42285 |
| NSDHL | Q15738 |
| WDR37 | Q9Y2I8 |
| UBE2N | P61088 |
| TTC8 | Q8TAM2 |
| CSNK2A2 | P19784 |
| ANGPTL2 | Q9UKU9 |
| NIN | Q8N4C6 |
| DDX23 | Q9BUQ8 |
| MICB | Q29980 |
| ST3GAL4 | Q11206 |
| CXCL13 | O43927 |
| FBXO32 | Q969P5 |
| PRKCB | P05771 |
| NCOR2 | Q9Y618 |
| TREH | O43280 |
| RPS25 | P62851 |
| DDOST | P39656 |
| HSPA6 | P17066 |
| EEF1A1 | P68104 |
| FOXF2 | Q12947 |
| MFGE8 | Q08431 |
| PTP4A3 | O75365 |
| NR0B2 | Q15466 |
| ZNRF3 | Q9ULT6 |
| KCTD5 | Q9NXV2 |
| HAPSTR1 | Q14CZ0 |
| CLP1 | Q92989 |
| SNRPD1 | P62314 |
| TBP | P20226 |
| STRADA | Q7RTN6 |
| GDF5 | P43026 |
| EGLN1 | Q9GZT9 |
| LYN | P07948 |
| CHTOP | Q9Y3Y2 |
| MED15 | Q96RN5 |
| WDR45 | Q9Y484 |
| MVK | Q03426 |
| PSMD8 | P48556 |
| REEP1 | Q9H902 |
| SSTR4 | P31391 |
| SEPTIN5 | Q99719 |
| CLTCL1 | P53675 |
| ELOVL4 | Q9GZR5 |
| PPP1R13L | Q8WUF5 |
| AKR1C4 | P17516 |
| IFIT1 | P09914 |
| TFR2 | Q9UP52 |
| NUMB | P49757 |
| SCAP | Q12770 |
| BCAT1 | P54687 |
| REEP2 | Q9BRK0 |
| PGAM1 | P18669 |
| PSTPIP1 | O43586 |
| NFIB | O00712 |
| CNTN4 | Q8IWV2 |
| HNRNPM | P52272 |
| BCL7A | Q4VC05 |
| GATAD2A | Q86YP4 |
| BOC | Q9BWV1 |
| CCL23 | P55773 |
| SAMHD1 | Q9Y3Z3 |
| ADSL | P30566 |
| RAB6A | P20340 |
| ATAD5 | Q96QE3 |
| FBXO31 | Q5XUX0 |
| GP2 | P55259 |
| SLC4A7 | Q9Y6M7 |
| C1QBP | Q07021 |
| FADS2 | O95864 |
| GJC1 | P36383 |
| SUMO1 | P63165 |
| PSMC6 | P62333 |
| AHCTF1 | Q8WYP5 |
| SMAD9 | O15198 |
| TMEM86A | Q8N2M4 |
| CXCL11 | O14625 |
| MLIP | Q5VWP3 |
| SENP1 | Q9P0U3 |
| ARFGAP3 | Q9NP61 |
| SMURF2 | Q9HAU4 |
| PSMD7 | P51665 |
| CHFR | Q96EP1 |
| NAT1 | P18440 |
| NARS2 | Q96I59 |
| NMB | P08949 |
| KIRREL1 | Q96J84 |
| DIO2 | Q92813 |
| SLCO4A1 | Q96BD0 |
| SCN10A | Q9Y5Y9 |
| PPP2CA | P67775 |
| AGPAT1 | Q99943 |
| CD52 | P31358 |
| HEYL | Q9NQ87 |
| MIR17HG | Q75NE6 |
| PRR4 | Q16378 |
| KHDC3L | Q587J8 |
| GPR161 | Q8N6U8 |
| TXNL4A | P83876 |
| TUBB1 | Q9H4B7 |
| SORD | Q00796 |
| TREX1 | Q9NSU2 |
| CD58 | P19256 |
| FAT2 | Q9NYQ8 |
| FOXD1 | Q16676 |
| CA8 | P35219 |
| CCL8 | P80075 |
| PGAP1 | Q75T13 |
| TRAPPC10 | P48553 |
| CCNT1 | O60563 |
| TBX2 | Q13207 |
| CST6 | Q15828 |
| RAX | Q9Y2V3 |
| HSPA12A | O43301 |
| MSTO1 | Q9BUK6 |
| CPSF4 | O95639 |
| ALDH1A3 | P47895 |
| SFXN4 | Q6P4A7 |
| EBF1 | Q9UH73 |
| PLCB4 | Q15147 |
| CEP70 | Q8NHQ1 |
| PIM1 | P11309 |
| F11 | P03951 |
| BRD2 | P25440 |
| C19orf12 | Q9NSK7 |
| GZMA | P12544 |
| GNLY | P22749 |
| USP10 | Q14694 |
| HOXB5 | P09067 |
| MAP2K7 | O14733 |
| DIS3L2 | Q8IYB7 |
| IMMT | Q16891 |
| ITGA9 | Q13797 |
| GOPC | Q9HD26 |
| FBXO5 | Q9UKT4 |
| ZMYND11 | Q15326 |
| ASIC1 | P78348 |
| CCL19 | Q99731 |
| CLSPN | Q9HAW4 |
| LAT | O43561 |
| HBQ1 | P09105 |
| IGF2-AS | Q6U949 |
| NCBP1 | Q09161 |
| HIBCH | Q6NVY1 |
| ATP5PB | P24539 |
| GCNT2 | Q8N0V5 |
| GPC1 | P35052 |
| TRA2B | P62995 |
| SNTB2 | Q13425 |
| HK1 | P19367 |
| MEPE | Q9NQ76 |
| ASCL1 | P50553 |
| PER2 | O15055 |
| INTS10 | Q9NVR2 |
| IGKC | P01834 |
| GTF3C1 | Q12789 |
| COIL | P38432 |
| RPLP1 | P05386 |
| WDR45B | Q5MNZ6 |
| CCDC28B | Q9BUN5 |
| SLX4IP | Q5VYV7 |
| LAMA1 | P25391 |
| IMMP2L | Q96T52 |
| ADIPOR2 | Q86V24 |
| SCTR | P47872 |
| IFNAR2 | P48551 |
| UBE2S | Q16763 |
| BAMBI | Q13145 |
| SLC35A3 | Q9Y2D2 |
| NRGN | Q92686 |
| TUBB2B | Q9BVA1 |
| MPDU1 | O75352 |
| ANXA6 | P08133 |
| PSMB3 | P49720 |
| PIK3C3 | Q8NEB9 |
| MAPK7 | Q13164 |
| LETM1 | O95202 |
| ARFGAP1 | Q8N6T3 |
| RAB3IL1 | Q8TBN0 |
| ARFGAP2 | Q8N6H7 |
| ASH2L | Q9UBL3 |
| RHD | Q02161 |
| ARHGDIA | P52565 |
| TLN1 | Q9Y490 |
| HNRNPU | Q00839 |
| FABP12 | A6NFH5 |
| ATAD3B | Q5T9A4 |
| NCK1 | P16333 |
| CCNB3 | Q8WWL7 |
| PARVA | Q9NVD7 |
| RAB33B | Q9H082 |
| IMPA1 | P29218 |
| FBXL4 | Q9UKA2 |
| PRKAR1B | P31321 |
| NR2F1 | P10589 |
| PEMT | Q9UBM1 |
| ABCC11 | Q96J66 |
| SIX6 | O95475 |
| PPA2 | Q9H2U2 |
| MXD1 | Q05195 |
| TRPC1 | P48995 |
| IGHA1 | P01876 |
| NHERF2 | Q15599 |
| TCEA3 | O75764 |
| PLCZ1 | Q86YW0 |
| BAG2 | O95816 |
| HRH1 | P35367 |
| PPP2R2B | Q00005 |
| TMEM107 | Q6UX40 |
| PNKD | Q8N490 |
| CSN2 | P05814 |
| WDR5 | P61964 |
| CRX | O43186 |
| MERTK | Q12866 |
| RHCE | P18577 |
| CD200 | P41217 |
| KPRP | Q5T749 |
| FABP3 | P05413 |
| TMEM38B | Q9NVV0 |
| ATXN3 | P54252 |
| KAT2A | Q92830 |
| SYNE3 | Q6ZMZ3 |
| CHCHD4 | Q8N4Q1 |
| TRIM50 | Q86XT4 |
| NXF2 | Q9GZY0 |
| CAST | P20810 |
| IST1 | P53990 |
| GLO1 | Q04760 |
| DGCR2 | P98153 |
| DGCR6 | Q14129 |
| FDPS | P14324 |
| ADAMTS17 | Q8TE56 |
| GFM1 | Q96RP9 |
| CNTROB | Q8N137 |
| KNL1 | Q8NG31 |
| PMM1 | Q92871 |
| ACLY | P53396 |
| ZFYVE26 | Q68DK2 |
| CRABP2 | P29373 |
| NMT1 | P30419 |
| SYTL2 | Q9HCH5 |
| IL3RA | P26951 |
| PITX1 | P78337 |
| KIF1C | O43896 |
| ADH4 | P08319 |
| SPATA7 | Q9P0W8 |
| CRISP3 | P54108 |
| CRAT | P43155 |
| PRR32 | B1ATL7 |
| LIMS2 | Q7Z4I7 |
| DLX6 | P56179 |
| PSMA3 | P25788 |
| HLA-DMB | P28068 |
| NUP88 | Q99567 |
| SIN3A | Q96ST3 |
| GPNMB | Q14956 |
| PDLIM5 | Q96HC4 |
| MTHFD2 | P13995 |
| PAF1 | Q8N7H5 |
| SF3A3 | Q12874 |
| SLC25A11 | Q02978 |
| ATP13A2 | Q9NQ11 |
| HJV | Q6ZVN8 |
| USP2 | O75604 |
| GOLGB1 | Q14789 |
| COASY | Q13057 |
| SERAC1 | Q96JX3 |
| ATP2A1 | O14983 |
| MYBBP1A | Q9BQG0 |
| DNAJC19 | Q96DA6 |
| LY96 | Q9Y6Y9 |
| SCART1 | Q4G0T1 |
| AFF4 | Q9UHB7 |
| HECTD4 | Q9Y4D8 |
| ARHGAP18 | Q8N392 |
| C5AR1 | P21730 |
| RNF4 | P78317 |
| HEY1 | Q9Y5J3 |
| DAP3 | P51398 |
| PSORS1C1 | Q9UIG5 |
| DAB2IP | Q5VWQ8 |
| FAM218A | Q96MZ4 |
| NDRG2 | Q9UN36 |
| PSMD9 | O00233 |
| RPS3A | P61247 |
| LSM2 | Q9Y333 |
| ALYREF | Q86V81 |
| PIAS1 | O75925 |
| CUX1 | Q13948 |
| CBFB | Q13951 |
| PNPLA2 | Q96AD5 |
| POLDIP3 | Q9BY77 |
| LCA5 | Q86VQ0 |
| CEP104 | O60308 |
| DBI | P07108 |
| MCU | Q8NE86 |
| WASL | O00401 |
| NUAK2 | Q9H093 |
| UTS2 | O95399 |
| WNT6 | Q9Y6F9 |
| DNAJB4 | Q9UDY4 |
| ZNF74 | Q16587 |
| DGCR6L | Q9BY27 |
| HOXD12 | P35452 |
| LAMB1 | P07942 |
| UGT2B7 | P16662 |
| PGK2 | P07205 |
| MT2A | P02795 |
| ZNF23 | P17027 |
| NISCH | Q9Y2I1 |
| VAMP4 | O75379 |
| SNAP23 | O00161 |
| PSME3 | P61289 |
| GDF1 | P27539 |
| FAM161A | Q3B820 |
| RASA2 | Q15283 |
| PLAC1 | Q9HBJ0 |
| GUCY2D | Q02846 |
| CYB5R3 | P00387 |
| ILF2 | Q12905 |
| WAPL | Q7Z5K2 |
| STAT6 | P42226 |
| PDCD10 | Q9BUL8 |
| APOBEC3A | P31941 |
| PCDHGA8 | Q9Y5G5 |
| SPI1 | P17947 |
| OPRD1 | P41143 |
| XRN2 | Q9H0D6 |
| SOX5 | P35711 |
| HNRNPC | P07910 |
| TTF2 | Q9UNY4 |
| CILK1 | Q9UPZ9 |
| OSGIN2 | Q9Y236 |
| ZNF597 | Q96LX8 |
| GABRG3 | Q99928 |
| MRPS14 | O60783 |
| KRT86 | O43790 |
| OGN | P20774 |
| RSPH4A | Q5TD94 |
| CKAP5 | Q14008 |
| CRYBB3 | P26998 |
| MCM10 | Q7L590 |
| IRF8 | Q02556 |
| PHOX2A | O14813 |
| PHC3 | Q8NDX5 |
| PLCG2 | P16885 |
| PATZ1 | Q9HBE1 |
| MALL | Q13021 |
| ADGRG1 | Q9Y653 |
| CXCR5 | P32302 |
| TOP3B | O95985 |
| VAPA | Q9P0L0 |
| FAT1 | Q14517 |
| EIF5 | P55010 |
| SLC2A4RG | Q9NR83 |
| PIGW | Q7Z7B1 |
| HEPACAM | Q14CZ8 |
| CHRNA4 | P43681 |
| REV1 | Q9UBZ9 |
| VPS52 | Q8N1B4 |
| RBMX | P38159 |
| MAGED1 | Q9Y5V3 |
| SUPT16H | Q9Y5B9 |
| MMAB | Q96EY8 |
| RPS13 | P62277 |
| ATXN7 | O15265 |
| CDKAL1 | Q5VV42 |
| IRAK4 | Q9NWZ3 |
| TCHH | Q07283 |
| KIF22 | Q14807 |
| CTH | P32929 |
| PINK1 | Q9BXM7 |
| TLR10 | Q9BXR5 |
| VAMP2 | P63027 |
| CBR3 | O75828 |
| NME3 | Q13232 |
| PTPN2 | P17706 |
| EIF4A1 | P60842 |
| WDHD1 | O75717 |
| PRPF3 | O43395 |
| LAMTOR2 | Q9Y2Q5 |
| CBR1 | P16152 |
| ATXN1 | P54253 |
| FBLN2 | P98095 |
| RAPGEF1 | Q13905 |
| GJA3 | Q9Y6H8 |
| CEBPD | P49716 |
| FDFT1 | P37268 |
| MILR1 | Q7Z6M3 |
| DNM1 | Q05193 |
| MYO7A | Q13402 |
| WWTR1 | Q9GZV5 |
| WLS | Q5T9L3 |
| PTPRU | Q92729 |
| COL24A1 | Q17RW2 |
| EGFL7 | Q9UHF1 |
| DNAJB1 | P25685 |
| C8B | P07358 |
| REG3A | Q06141 |
| USP1 | O94782 |
| UROS | P10746 |
| TRPC4 | Q9UBN4 |
| YTHDF2 | Q9Y5A9 |
| LRIG2 | O94898 |
| SMOX | Q9NWM0 |
| NPTX1 | Q15818 |
| ELAVL3 | Q14576 |
| COMMD1 | Q8N668 |
| CCDC88C | Q9P219 |
| ACOT7 | O00154 |
| BMP3 | P12645 |
| NRF1 | Q16656 |
| FGF6 | P10767 |
| NEK7 | Q8TDX7 |
| RPL39 | P62891 |
| MFN1 | Q8IWA4 |
| SLC46A1 | Q96NT5 |
| RTN4 | Q9NQC3 |
| NUP98 | P52948 |
| HMGCL | P35914 |
| IL37 | Q9NZH6 |
| ITGB8 | P26012 |
| DMWD | Q09019 |
| KIF5A | Q12840 |
| CA2 | P00918 |
| IRF9 | Q00978 |
| MTNR1B | P49286 |
| PDHX | O00330 |
| AMPD1 | P23109 |
| SLC16A12 | Q6ZSM3 |
| TMEM114 | B3SHH9 |
| OGDH | Q02218 |
| GAMT | Q14353 |
| RAB3IP | Q96QF0 |
| ACOT13 | Q9NPJ3 |
| EFNB2 | P52799 |
| PRKAR2A | P13861 |
| GBP1 | P32455 |
| DDAH2 | O95865 |
| GDF11 | O95390 |
| ARL17A | Q8IVW1 |
| GTF2H1 | P32780 |
| LPO | P22079 |
| KLK2 | P20151 |
| ITGA1 | P56199 |
| GTF2H4 | Q92759 |
| CHRNA9 | Q9UGM1 |
| UBE2L3 | P68036 |
| IRF2BPL | Q9H1B7 |
| RPS6KA1 | Q15418 |
| PPIF | P30405 |
| HTR5A | P47898 |
| NOL11 | Q9H8H0 |
| MATN1 | P21941 |
| COPZ1 | P61923 |
| TONSL | Q96HA7 |
| CHIC1 | Q5VXU3 |
| ARID4B | Q4LE39 |
| HRG | P04196 |
| RPL30 | P62888 |
| RNASEH2B | Q5TBB1 |
| RPS11 | P62280 |
| ENTPD5 | O75356 |
| BCKDK | O14874 |
| TRMT2A | Q8IZ69 |
| SNX11 | Q9Y5W9 |
| CCNL2 | Q96S94 |
| EEA1 | Q15075 |
| OR13F1 | Q8NGS4 |
| PANK2 | Q9BZ23 |
| RPL23A | P62750 |
| ANO10 | Q9NW15 |
| TIMM10 | P62072 |
| DUX4 | Q9UBX2 |
| GALNT17 | Q6IS24 |
| FMO1 | Q01740 |
| KIR2DL1 | P43626 |
| PFKP | Q01813 |
| ARF4 | P18085 |
| DPY30 | Q9C005 |
| ITPR1 | Q14643 |
| SERPIND1 | P05546 |
| RNASEH2C | Q8TDP1 |
| PRSS3 | P35030 |
| ENDOG | Q14249 |
| AQP8 | O94778 |
| TMEM87A | Q8NBN3 |
| ZDHHC15 | Q96MV8 |
| NUDT6 | P53370 |
| BRD7 | Q9NPI1 |
| PITX3 | O75364 |
| MAK16 | Q9BXY0 |
| NOP2 | P46087 |
| FPR1 | P21462 |
| COPS6 | Q7L5N1 |
| NDUFA4 | O00483 |
| CEP55 | Q53EZ4 |
| PCSK2 | P16519 |
| MYL9 | P24844 |
| TEX29 | Q8N6K0 |
| TENM3 | Q9P273 |
| IPO5 | O00410 |
| LTV1 | Q96GA3 |
| IMPDH1 | P20839 |
| BCORL1 | Q5H9F3 |
| SLC6A9 | P48067 |
| LRIT1 | Q9P2V4 |
| TIMM22 | Q9Y584 |
| ZNF365 | Q70YC4 |
| CSPG4 | Q6UVK1 |
| HSPA1B | P0DMV9 |
| FES | P07332 |
| ELOA | Q14241 |
| EIF5AL1 | Q6IS14 |
| ATP1A3 | P13637 |
| MAIP1 | Q8WWC4 |
| LMBRD1 | Q9NUN5 |
| NME4 | O00746 |
| OPHN1 | O60890 |
| ZNF142 | P52746 |
| TUBB2A | Q13885 |
| VASN | Q6EMK4 |
| DDHD1 | Q8NEL9 |
| PRMT7 | Q9NVM4 |
| NKX2-2 | O95096 |
| CYP26A1 | O43174 |
| FERD3L | Q96RJ6 |
| SACM1L | Q9NTJ5 |
| FOSB | P53539 |
| DSTN | P60981 |
| RANBP9 | Q96S59 |
| POC1B | Q8TC44 |
| MRPL40 | Q9NQ50 |
| EIF5B | O60841 |
| SLC6A2 | P23975 |
| DCTN2 | Q13561 |
| CLASP1 | Q7Z460 |
| NCR1 | O76036 |
| TLE1 | Q04724 |
| MBTPS2 | O43462 |
| CHST11 | Q9NPF2 |
| ACBD5 | Q5T8D3 |
| CYP2J2 | P51589 |
| SYNGR1 | O43759 |
| INHBE | P58166 |
| COX11 | Q9Y6N1 |
| ABHD11 | Q8NFV4 |
| PDE3B | Q13370 |
| AP5Z1 | O43299 |
| LENG1 | Q96BZ8 |
| ALPI | P09923 |
| PSMA2 | P25787 |
| ISG20 | Q96AZ6 |
| STARD3 | Q14849 |
| PRICKLE4 | Q2TBC4 |
| HES5 | Q5TA89 |
| AKT1S1 | Q96B36 |
| MTMR1 | Q13613 |
| CHRNB4 | P30926 |
| SYNCRIP | O60506 |
| IGFL3 | Q6UXB1 |
| SH3KBP1 | Q96B97 |
| NDUFS8 | O00217 |
| ANO6 | Q4KMQ2 |
| IDE | P14735 |
| ZBTB18 | Q99592 |
| ACTN1 | P12814 |
| CSTF1 | Q05048 |
| POU2F1 | P14859 |
| ACAA1 | P09110 |
| SLC1A3 | P43003 |
| TIMM29 | Q9BSF4 |
| MT-RNR2 | Q8IVG9 |
| MAPK9 | P45984 |
| POM121C | A8CG34 |
| CES1 | P23141 |
| VANGL2 | Q9ULK5 |
| RXFP2 | Q8WXD0 |
| RAB18 | Q9NP72 |
| KCNJ16 | Q9NPI9 |
| ANKK1 | Q8NFD2 |
| POLM | Q9NP87 |
| PEG10 | Q86TG7 |
| PSMC1 | P62191 |
| GABRB3 | P28472 |
| FKBP5 | Q13451 |
| TGM6 | O95932 |
| SLC2A6 | Q9UGQ3 |
| ACTR3 | P61158 |
| PTPRT | O14522 |
| DNAH9 | Q9NYC9 |
| ZFR | Q96KR1 |
| INSIG1 | O15503 |
| DCT | P40126 |
| THAP1 | Q9NVV9 |
| RRBP1 | Q9P2E9 |
| MYH14 | Q7Z406 |
| CDK11B | P21127 |
| FYTTD1 | Q96QD9 |
| DCLK1 | O15075 |
| PCTP | Q9UKL6 |
| SLC25A29 | Q8N8R3 |
| ACOT12 | Q8WYK0 |
| KRTAP9-9 | Q9BYP9 |
| C1QA | P02745 |
| PSMD10 | O75832 |
| ZC4H2 | Q9NQZ6 |
| HLTF | Q14527 |
| CAPZB | P47756 |
| TIMM8A | O60220 |
| DST | Q03001 |
| CELF1 | Q92879 |
| PARPBP | Q9NWS1 |
| UNC5A | Q6ZN44 |
| SRPK3 | Q9UPE1 |
| USP26 | Q9BXU7 |
| ZNF12 | P17014 |
| PDLIM3 | Q53GG5 |
| LAMP3 | Q9UQV4 |
| CLINT1 | Q14677 |
| TULP1 | O00294 |
| BMP5 | P22003 |
| TFF2 | Q03403 |
| CSNK2B | P67870 |
| HNRNPF | P52597 |
| AIPL1 | Q9NZN9 |
| HNRNPD | Q14103 |
| GNB1L | Q9BYB4 |
| CREM | Q03060 |
| SLC25A19 | Q9HC21 |
| VMA21 | Q3ZAQ7 |
| RASIP1 | Q5U651 |
| GRIK2 | Q13002 |
| COL14A1 | Q05707 |
| P3H2 | Q8IVL5 |
| APOBEC3G | Q9HC16 |
| SSR4 | P51571 |
| ADCY5 | O95622 |
| DNAI2 | Q9GZS0 |
| ZNHIT3 | Q15649 |
| CCM2 | Q9BSQ5 |
| CDK3 | Q00526 |
| KIRREL2 | Q6UWL6 |
| MARK4 | Q96L34 |
| KPNA3 | O00505 |
| LSM4 | Q9Y4Z0 |
| CMTM5 | Q96DZ9 |
| CFAP100 | Q494V2 |
| SPDYE4 | A6NLX3 |
| PRR25 | Q96S07 |
| HNRNPR | O43390 |
| FMOD | Q06828 |
| TUBA1C | Q9BQE3 |
| CCDC88A | Q3V6T2 |
| PDSS2 | Q86YH6 |
| CHRNA5 | P30532 |
| BOD1L1 | Q8NFC6 |
| HMCN1 | Q96RW7 |
| PIGN | O95427 |
| FERMT2 | Q96AC1 |
| MCF2 | P10911 |
| GBGT1 | Q8N5D6 |
| CEP78 | Q5JTW2 |
| NCCRP1 | Q6ZVX7 |
| PVALB | P20472 |
| CD33 | P20138 |
| PDE6D | O43924 |
| PRPF19 | Q9UMS4 |
| ACR | P10323 |
| FCER2 | P06734 |
| BPTF | Q12830 |
| DDA1 | Q9BW61 |
| PEX5L | Q8IYB4 |
| SLC39A4 | Q6P5W5 |
| MBD1 | Q9UIS9 |
| AP1M1 | Q9BXS5 |
| EEF1D | P29692 |
| U2AF2 | P26368 |
| TMEM201 | Q5SNT2 |
| ZNF71 | Q9NQZ8 |
| EZH1 | Q92800 |
| CHRNB2 | P17787 |
| ALDH1B1 | P30837 |
| TNFAIP8L2 | Q6P589 |
| METTL8 | Q9H825 |
| FGF20 | Q9NP95 |
| PNLIPRP1 | P54315 |
| POC5 | Q8NA72 |
| C1QB | P02746 |
| NAP1L5 | Q96NT1 |
| PPP1R14A | Q96A00 |
| CHRM1 | P11229 |
| AHSP | Q9NZD4 |
| TNFSF8 | P32971 |
| TNFRSF9 | Q07011 |
| RBM14 | Q96PK6 |
| DDX21 | Q9NR30 |
| HNRNPA3 | P51991 |
| RPL17 | P18621 |
| SLC45A2 | Q9UMX9 |
| BRPF1 | P55201 |
| TAF1D | Q9H5J8 |
| ADGRV1 | Q8WXG9 |
| TAF15 | Q92804 |
| OASL | Q15646 |
| GTF3A | Q92664 |
| PPIC | P45877 |
| ARID3A | Q99856 |
| GLMN | Q92990 |
| INTS4 | Q96HW7 |
| NRN1 | Q9NPD7 |
| STX12 | Q86Y82 |
| GABRB2 | P47870 |
| EML1 | O00423 |
| ENHO | Q6UWT2 |
| DERL1 | Q9BUN8 |
| RBM3 | P98179 |
| RRAS | P10301 |
| TRPM4 | Q8TD43 |
| INPP5D | Q92835 |
| SLC38A2 | Q96QD8 |
| SIN3B | O75182 |
| CAMK2G | Q13555 |
| RFX5 | P48382 |
| CPB1 | P15086 |
| PAM16 | Q9Y3D7 |
| TIMM23 | O14925 |
| LTA4H | P09960 |
| DRG1 | Q9Y295 |
| TUBA4A | P68366 |
| OR4C3 | Q8NH37 |
| ARGFX | A6NJG6 |
| MYOC | Q99972 |
| DHODH | Q02127 |
| CTRL | P40313 |
| LILRB1 | Q8NHL6 |
| ANO8 | Q9HCE9 |
| OLIG1 | Q8TAK6 |
| EPS15 | P42566 |
| TCEA2 | Q15560 |
| SURF6 | O75683 |
| TKFC | Q3LXA3 |
| BAIAP3 | O94812 |
| USP15 | Q9Y4E8 |
| MED23 | Q9ULK4 |
| NONO | Q15233 |
| CNR2 | P34972 |
| MORF4L2 | Q15014 |
| NCF2 | P19878 |
| RFX7 | Q2KHR2 |
| TPPP3 | Q9BW30 |
| CTSF | Q9UBX1 |
| HOXB1 | P14653 |
| BTBD18 | B2RXH4 |
| PSMB1 | P20618 |
| MDH2 | P40926 |
| ZNF496 | Q96IT1 |
| INSRR | P14616 |
| LMLN | Q96KR4 |
| PANK1 | Q8TE04 |
| RAB21 | Q9UL25 |
| EPS8L1 | Q8TE68 |
| ZNF214 | Q9UL59 |
| H2BW2 | P0C1H6 |
| TIMM13 | Q9Y5L4 |
| MAN2A1 | Q16706 |
| JPH3 | Q8WXH2 |
| MBNL2 | Q5VZF2 |
| MBNL3 | Q9NUK0 |
| YIPF7 | Q8N8F6 |
| DUS3L | Q96G46 |
| EMX2 | Q04743 |
| MCCC2 | Q9HCC0 |
| SHANK3 | Q9BYB0 |
| DRD5 | P21918 |
| INTS8 | Q75QN2 |
| NEFM | P07197 |
| RXFP1 | Q9HBX9 |
| CYP2U1 | Q7Z449 |
| CD48 | P09326 |
| IRGM | A1A4Y4 |
| ZFHX2 | Q9C0A1 |
| BATF2 | Q8N1L9 |
| ECT2 | Q9H8V3 |
| CEP89 | Q96ST8 |
| GTF2B | Q00403 |
| BHMT | Q93088 |
| CA4 | P22748 |
| ECI2 | O75521 |
| CLIC5 | Q9NZA1 |
| FGF16 | O43320 |
| CELF2 | O95319 |
| CCL28 | Q9NRJ3 |
| TIMM17A | Q99595 |
| TIMM17B | O60830 |
| STX5 | Q13190 |
| GYPE | P15421 |
| RBBP5 | Q15291 |
| PSME1 | Q06323 |
| PPP2CB | P62714 |
| CANT1 | Q8WVQ1 |
| SPNS2 | Q8IVW8 |
| SLC20A2 | Q08357 |
| HNRNPUL2 | Q1KMD3 |
| CPSF6 | Q16630 |
| MFHAS1 | Q9Y4C4 |
| AOX1 | Q06278 |
| HMG20B | Q9P0W2 |
| LRPAP1 | P30533 |
| ACKR2 | O00590 |
| TAF1L | Q8IZX4 |
| ADGRG6 | Q86SQ4 |
| SPRR1A | P35321 |
| UGT1A9 | O60656 |
| RAE1 | P78406 |
| DLG4 | P78352 |
| TSN | Q15631 |
| APOO | Q9BUR5 |
| TIMM23B | Q5SRD1 |
| KCTD13 | Q8WZ19 |
| COPS2 | P61201 |
| IFNL4 | K9M1U5 |
| PCDHA7 | Q9UN72 |
| B4GAT1 | O43505 |
| GATAD2B | Q8WXI9 |
| LEFTY1 | O75610 |
| HOXB3 | P14651 |
| GPSM1 | Q86YR5 |
| MCEE | Q96PE7 |
| TAX1BP1 | Q86VP1 |
| RPA3 | P35244 |
| ALDH3A1 | P30838 |
| AFG2A | Q8NB90 |
| GTPBP2 | Q9BX10 |
| REPS1 | Q96D71 |
| PANK3 | Q9H999 |
| CAMTA2 | O94983 |
| HOXB6 | P17509 |
| KCND2 | Q9NZV8 |
| LIN9 | Q5TKA1 |
| GOSR2 | O14653 |
| ADRA2C | P18825 |
| RHOH | Q15669 |
| LINC01587 | Q99440 |
| DGKB | Q9Y6T7 |
| MYO1F | O00160 |
| SEC23IP | Q9Y6Y8 |
| AVPR1A | P37288 |
| BYSL | Q13895 |
| TAF2 | Q6P1X5 |
| OSBPL8 | Q9BZF1 |
| PNPLA4 | P41247 |
| SEL1L | Q9UBV2 |
| RPL32 | P62910 |
| NEK4 | P51957 |
| UGT2B4 | P06133 |
| EBF2 | Q9HAK2 |
| NEUROG2 | Q9H2A3 |
| GDF10 | P55107 |
| COX6A1 | P12074 |
| TIMM10B | Q9Y5J6 |
| ATP2C1 | P98194 |
| ACOT8 | O14734 |
| DDX54 | Q8TDD1 |
| MINK1 | Q8N4C8 |
| ASF1A | Q9Y294 |
| SERPINA12 | Q8IW75 |
| NOLC1 | Q14978 |
| DDX5 | P17844 |
| SLCO2A1 | Q92959 |
| LCP2 | Q13094 |
| CHRNA2 | Q15822 |
| GRK3 | P35626 |
| CHRNA6 | Q15825 |
| CHRNB3 | Q05901 |
| MCHR1 | Q99705 |
| B3GNT5 | Q9BYG0 |
| CHRNA10 | Q9GZZ6 |
| DOK5 | Q9P104 |
| GDF7 | Q7Z4P5 |
| SLC2A12 | Q8TD20 |
| FGFR1OP2 | Q9NVK5 |
| HIVEP1 | P15822 |
| TENT5A | Q96IP4 |
| DNAI7 | Q6TDU7 |
| CITED4 | Q96RK1 |
| SGIP1 | Q9BQI5 |
| NYAP2 | Q9P242 |
| PRRC2A | P48634 |
| SSX2IP | Q9Y2D8 |
| BPGM | P07738 |
| CAMLG | P49069 |
| GABBR1 | Q9UBS5 |
| PAXIP1 | Q6ZW49 |
| NPRL3 | Q12980 |
| LDHB | P07195 |
| SFXN3 | Q9BWM7 |
| SFXN2 | Q96NB2 |
| SFXN5 | Q8TD22 |
| TIMM9 | Q9Y5J7 |
| TIMM21 | Q9BVV7 |
| TOMM5 | Q8N4H5 |
| SLC25A10 | Q9UBX3 |
| TBC1D4 | O60343 |
| WNT3A | P56704 |
| PLEKHA8 | Q96JA3 |
| ACTR2 | P61160 |
| ACADSB | P45954 |
| GGACT | Q9BVM4 |
| STRC | Q7RTU9 |
| SIM2 | Q14190 |
| GJB3 | O75712 |
| GJB4 | Q9NTQ9 |
| ATOX1 | O00244 |
| HIKESHI | Q53FT3 |
| NAPB | Q9H115 |
| MEOX1 | P50221 |
| ATXN2L | Q8WWM7 |
| FAM3C | Q92520 |
| PNPT1 | Q8TCS8 |
| UQCRC1 | P31930 |
| STPG2 | Q8N412 |
| ERLIN2 | O94905 |
| NAT8 | Q9UHE5 |
| EIF4A2 | Q14240 |
| ZNF283 | Q8N7M2 |
| MYOZ1 | Q9NP98 |
| DDX59 | Q5T1V6 |
| SLC20A1 | Q8WUM9 |
| CKMT2 | P17540 |
| PDIA6 | Q15084 |
| MCCC1 | Q96RQ3 |
| MRPS27 | Q92552 |
| CERK | Q8TCT0 |
| MRPL12 | P52815 |
| CCDC120 | Q96HB5 |
| MED4 | Q9NPJ6 |
| TEX14 | Q8IWB6 |
| PCDH15 | Q96QU1 |
| MAPK12 | P53778 |
| C7 | P10643 |
| MLC1 | Q15049 |
| ITSN2 | Q9NZM3 |
| BTAF1 | O14981 |
| APEX2 | Q9UBZ4 |
| KHSRP | Q92945 |
| PCBD1 | P61457 |
| PPP5C | P53041 |
| SHCBP1 | Q8NEM2 |
| BNIP2 | Q12982 |
| NCBP3 | Q53F19 |
| SETDB1 | Q15047 |
| ACAD11 | Q709F0 |
| PLK3 | Q9H4B4 |
| MRPS34 | P82930 |
| NPNT | Q6UXI9 |
| USH1C | Q9Y6N9 |
| ITPR2 | Q14571 |
| UBE4B | O95155 |
| CLDN2 | P57739 |
| TSPAN7 | P41732 |
| TRIP10 | Q15642 |
| B3GAT1 | Q9P2W7 |
| TRIM22 | Q8IYM9 |
| INTS3 | Q68E01 |
| NPLOC4 | Q8TAT6 |
| DIO1 | P49895 |
| KLHDC2 | Q9Y2U9 |
| HMOX2 | P30519 |
| HMGCS2 | P54868 |
| INTS13 | Q9NVM9 |
| FYCO1 | Q9BQS8 |
| RUSF1 | Q96GQ5 |
| TRPM3 | Q9HCF6 |
| GPAT3 | Q53EU6 |
| RAB27B | O00194 |
| GPAT4 | Q86UL3 |
| GPM6B | Q13491 |
| PRPF40A | O75400 |
| LSM6 | P62312 |
| PPP1CA | P62136 |
| STEEP1 | Q9H5V9 |
| AP2A1 | O95782 |
| FZD6 | O60353 |
| BPHL | Q86WA6 |
| HELZ | P42694 |
| CCN6 | O95389 |
| ORC2 | Q13416 |
| RFX1 | P22670 |
| USO1 | O60763 |
| ITIH4 | Q14624 |
| KPNA6 | O60684 |
| ZC3HAV1 | Q7Z2W4 |
| CRY2 | Q49AN0 |
| MAGEA11 | P43364 |
| ARID4A | P29374 |
| VIPR1 | P32241 |
| BHLHE22 | Q8NFJ8 |
| NDUFA12 | Q9UI09 |
| ARGLU1 | Q9NWB6 |
| CMKLR1 | Q99788 |
| CSNK1A1 | P48729 |
| UGT2B15 | P54855 |
| GSTZ1 | O43708 |
| CALCRL | Q16602 |
| RCC1 | P18754 |
| SEC22B | O75396 |
| RAB5C | P51148 |
| DNAJA3 | Q96EY1 |
| STAC2 | Q6ZMT1 |
| ACSF3 | Q4G176 |
| RP9 | Q8TA86 |
| SRRM2 | Q9UQ35 |
| CDC34 | P49427 |
| EP400 | Q96L91 |
| AP4B1 | Q9Y6B7 |
| RDH8 | Q9NYR8 |
| VTI1B | Q9UEU0 |
| PRR12 | Q9ULL5 |
| PCYT1A | P49585 |
| RNF31 | Q96EP0 |
| RTN1 | Q16799 |
| TSGA10 | Q9BZW7 |
| H2BC12L | P57053 |
| TPR | P12270 |
| RBM6 | P78332 |
| PAFAH1B2 | P68402 |
| MMP25 | Q9NPA2 |
| ICAM3 | P32942 |
| USF2 | Q15853 |
| PRR18 | Q8N4B5 |
| DNAJC7 | Q99615 |
| ETAA1 | Q9NY74 |
| ZKSCAN7 | Q9P0L1 |
| PICALM | Q13492 |
| ANAPC10 | Q9UM13 |
| SNRPF | P62306 |
| TRPV2 | Q9Y5S1 |
| NEIL2 | Q969S2 |
| STX10 | O60499 |
| KPNA1 | P52294 |
| EMC1 | Q8N766 |
| PDZD8 | Q8NEN9 |
| UGGT2 | Q9NYU1 |
| CDC5L | Q99459 |
| MAPK11 | Q15759 |
| CENPB | P07199 |
| TMEM147 | Q9BVK8 |
| TRPC5 | Q9UL62 |
| RTN3 | O95197 |
| RTRAF | Q9Y224 |
| RPS6KA5 | O75582 |
| STIM2 | Q9P246 |
| UBE2V2 | Q15819 |
| TRNT1 | Q96Q11 |
| ANAPC4 | Q9UJX5 |
| DDX20 | Q9UHI6 |
| TFAP4 | Q01664 |
| OSBPL2 | Q9H1P3 |
| CUL2 | Q13617 |
| HSD17B10 | Q99714 |
| GSTK1 | Q9Y2Q3 |
| UBE2E1 | P51965 |
| TRIM13 | O60858 |
| OS9 | Q13438 |
| CHMP4B | Q9H444 |
| EDEM1 | Q92611 |
| TMED10 | P49755 |
| GTF2F1 | P35269 |
| H2AC4 | P04908 |
| THRSP | Q92748 |
| SEC16B | Q96JE7 |
| USP4 | Q13107 |
| MCC | P23508 |
| STOM | P27105 |
| ZBTB38 | Q8NAP3 |
| TCF7 | P36402 |
| ZNF131 | P52739 |
| ATP1A2 | P50993 |
| CLCN6 | P51797 |
| OPCML | Q14982 |
| RALGAPA1 | Q6GYQ0 |
| TOPAZ1 | Q8N9V7 |
| COX4I2 | Q96KJ9 |
| BOK | Q9UMX3 |
| DGAT2 | Q96PD7 |
| CYP8B1 | Q9UNU6 |
| CIDEB | Q9UHD4 |
| ACAT2 | Q9BWD1 |
| USP8 | P40818 |
| CASTOR3 | Q8NAP1 |
| MSC | O60682 |
| TMED7 | Q9Y3B3 |
| TICAM2 | Q86XR7 |
| SEMA6C | Q9H3T2 |
| DCTN6 | O00399 |
| ZNRD2 | O60232 |
| CER1 | O95813 |
| SH2B2 | O14492 |
| TCFL5 | Q9UL49 |
| RGPD2 | P0DJD1 |
| LIN28A | Q9H9Z2 |
| HDX | Q7Z353 |
| GADD45B | O75293 |
| NPAS4 | Q8IUM7 |
| AGFG2 | O95081 |
| MAP3K15 | Q6ZN16 |
| TBPL2 | Q6SJ96 |
| CHDH | Q8NE62 |
| PACC1 | Q9H813 |
| NUDT11 | Q96G61 |
| ERN1 | O75460 |
| C6orf47 | O95873 |
| FAM215A | Q9Y5M1 |
| OXA1L | Q15070 |
| PER1 | O15534 |
| PDPK1 | O15530 |
| VTA1 | Q9NP79 |

**Supplemental Table 5.** Linearity and Content Determination of Wubie Fanchun Oral Liquid (n=3).

| Compound | Regression equation | r | Linear range/μg·mL^-1^ | Content determination（mg g^-1^） | RSD% |
| --- | --- | --- | --- | --- | --- |
| 2,3,5,4'-Tetrahydroxystilbene-2-O-β-D-glucoside | y = 1.3166x + 0.7667 | 0.9995 | 4.063~65.00 | 0.501 | 1.565 |
| Asperosaponin VI | y = 0.0566x + 0.3863 | 0.9991 | 31.25~500.0 | 4.116 | 2.156 |


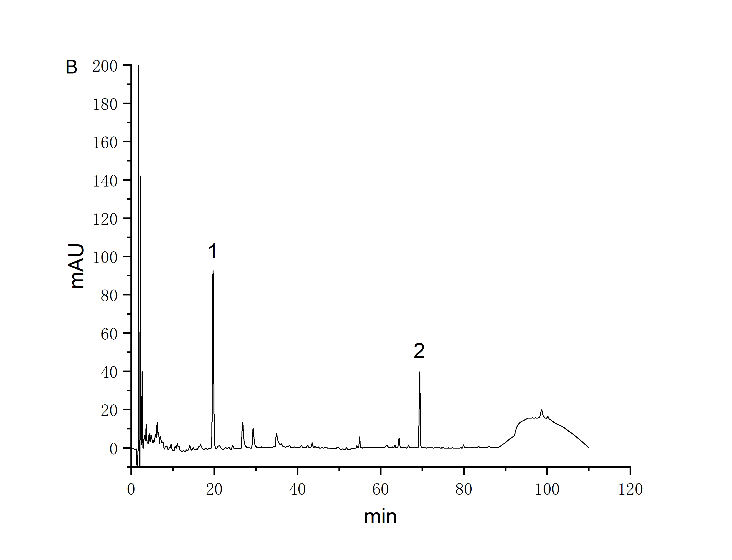


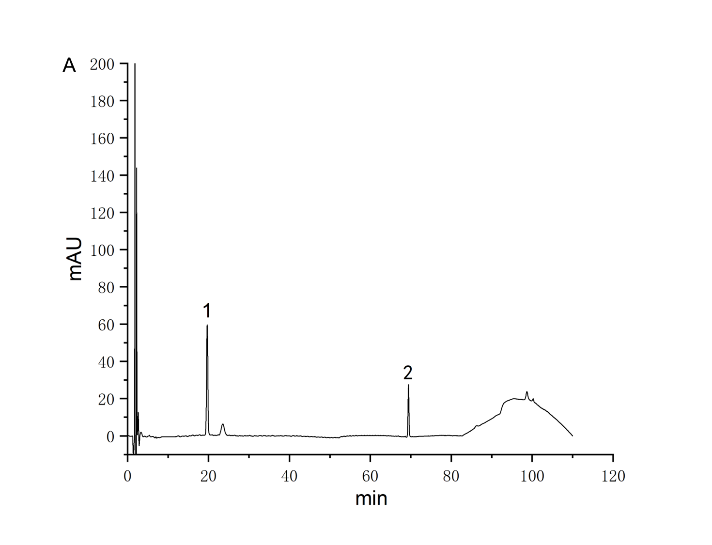


**Supplemental Figure 2.** Reference substance (A) and Wubie Fanchun Oral Liquid (B)

Peak1: 2,3,5,4'-Tetrahydroxystilbene-2-O-β-D-glucoside;

Peak 2: Asperosaponin VI.
